# Effectiveness of de-implementation of low-value healthcare practices: an overview of systematic reviews

**DOI:** 10.1186/s13012-024-01384-6

**Published:** 2024-08-05

**Authors:** Christina Kien, Julia Daxenbichler, Viktoria Titscher, Julia Baenziger, Pauline Klingenstein, Rahel Naef, Irma Klerings, Lauren Clack, Julian Fila, Isolde Sommer

**Affiliations:** 1 Department for Evidence-based Medicine and Evaluation, University for Continuing Education Krems (Danube University Krems), Dr.-Karl-Dorrek Straße 30, 3500 Krems a.d. Donau, Austria; 2 Institute for Implementation Science in Health Care, University of Zurich, Universitätstrasse 84, 8006 Zurich, Switzerland; 3 Centre of Clinical Nursing Science, University Hospital of Zurich, Rämistrasse 100, 8091 Zurich, Switzerland; 4 Department of Infectious Diseases and Hospital Epidemiology, University Hospital of Zurich, Rämistrasse 100, Zurich, 8091 Switzerland

**Keywords:** *de-*implementation, Low-value care, Antibiotic, Laboratory tests, Effectiveness, Overview of reviews

## Abstract

**Background:**

Reducing low-value care (LVC) is crucial to improve the quality of patient care while increasing the efficient use of scarce healthcare resources. Recently, strategies to de-implement LVC have been mapped against the Expert Recommendation for Implementing Change (ERIC) compilation of strategies. However, such strategies’ effectiveness across different healthcare practices has not been addressed. This overview of systematic reviews aimed to investigate the effectiveness of de-implementation initiatives and specific ERIC strategy clusters.

**Methods:**

We searched MEDLINE (Ovid), Epistemonikos.org and Scopus (Elsevier) from 1 January 2010 to 17 April 2023 and used additional search strategies to identify relevant systematic reviews (SRs). Two reviewers independently screened abstracts and full texts against a priori–defined criteria, assessed the SR quality and extracted pre-specified data. We created harvest plots to display the results.

**Results:**

Of 46 included SRs, 27 focused on drug treatments, such as antibiotics or opioids, twelve on laboratory tests or diagnostic imaging and seven on other healthcare practices. In categorising de-implementation strategies, SR authors applied different techniques: creating self-developed strategies (*n* = 12), focussing on specific de-implementation strategies (*n* = 14) and using published taxonomies (*n* = 12). Overall, 15 SRs provided evidence for the effectiveness of de-implementation interventions to reduce antibiotic and opioid utilisation. Reduced utilisation, albeit inconsistently significant, was documented in the use of antipsychotics and benzodiazepines, as well as in laboratory tests and diagnostic imaging. Strategies within the *adapt and tailor to context*, *develop stakeholder interrelationships*, and *change infrastructure and workflow* ERIC clusters led to a consistent reduction in LVC practices.

**Conclusion:**

De-implementation initiatives were effective in reducing medication usage, and inconsistent significant reductions were observed for LVC laboratory tests and imaging. Notably, de-implementation clusters such as *change infrastructure and workflow* and *develop stakeholder interrelationships* emerged as the most encouraging avenues. Additionally, we provided suggestions to enhance SR quality, emphasising adherence to guidelines for synthesising complex interventions, prioritising appropriateness of care outcomes, documenting the development process of de-implementation initiatives and ensuring consistent reporting of applied de-implementation strategies.

**Registration:**

OSF Open Science Framework 5ruzw.

**Supplementary Information:**

The online version contains supplementary material available at 10.1186/s13012-024-01384-6.

Contributions to the literature

- This overview of systematic reviews contributes a synthesis on the effectiveness of de-implementation initiatives and associated de-implementation strategies to reduce low-value care (LVC) for patients.
- It highlights that the de-implementation of some healthcare practices, such as antibiotic or opioid treatments, diagnostic imaging and laboratory testing, has been studied extensively. The evidence base of other LVCs, such as cancer screening and surgery, is less well synthesised.
- *Change infrastructure and workflow* and *develop stakeholder interrelationships* successfully reduced LVC across several healthcare practices.
- This study identifies barriers to synthesising the evidence in SRs and provides recommendations to overcome these.

## Background

Low-value care (LVC) is an umbrella term summarising healthcare practices that provide a patient minimal or no health benefit, where the benefits do not outweigh the associated harms or are relevant for only a small proportion of patients [1]. LVC use is a global phenomenon. The estimations of patients receiving at least one inappropriate healthcare practice annually range from 12 to 15% [2, 3]. Reducing LVC is important since it helps improve the quality of provided services while also supporting the efficient use of scarce financial healthcare resources [4]. Established healthcare practices can be challenging to reduce or eliminate [5].

De-implementation is the systematic process of discontinuing LVC, namely, to remove (i.e. stop the delivery entirely), replace (i.e. stop an inappropriate intervention and start a new, evidence-based intervention targeting similar aims), reduce (i.e. change the practice frequency and/or intensity), or restrict (i.e. specify a particular setting/population) LVC practices [5]. While *implementation strategies* generally refer to the methods or techniques used to facilitate the adoption, implementation, or sustainment of interventions, *de-implementation strategies* refer to those same methods and techniques when applied to reduce LVC practices [6].

Taxonomies have been developed to enable consistent reporting of implementation strategies. Recently, two scoping reviews [6, 7] and one methodological study [8] identified de-implementation strategies applied in healthcare settings, relying on existing taxonomies to categorise those sharing similar attributes. The taxonomies used were the Expert Recommendations for Implementing Change (ERIC) [9–11], the taxonomy developed by the Cochrane Effective Practice and Organization of Care (EPOC) Review Group [12, 13] and the behaviour change technique taxonomy from Michie and colleagues [12, 13]. Common examples of the first two taxonomies are ‘audit and provide feedback’, ‘conduct educational meetings’, and ‘use financial incentives’. However, both scoping reviews highlighted the necessity to adapt and extend existing taxonomies to capture all de-implementation strategy variations. For example, one scoping review added ‘accountability tools’ (which reminded clinicians not to use a certain LVC and held them accountable for still applying it) and ‘communication tools’ (which established a common physician–patient understanding by employing a conversation guide or shared decision-making model to avoid LVC) to their taxonomy [6]. In this article, we differentiate between de-implementation strategy and de-implementation initiative. De-implementation strategy pertains to individual methods and techniques, whereas de-implementation initiative encompasses the collective array of strategies employed, often in diverse combinations.

While several reviews have been conducted in the field, the effectiveness of LVC de-implementation initiatives and strategies has not yet been systematically synthesised across different healthcare practices [14]. Thus, we aimed to address this gap using an overview of systematic reviews (i.e. umbrella review) approach [15].

## Methods

### Study design

We conducted an overview of systematic reviews in accordance with Cochrane guidance [15] and followed the preferred reporting items for overviews of systematic reviews (PRIOR) statement of healthcare interventions [16] (see Additional File 2). We developed a protocol and registered it a priori (https://osf.io/5ruzw).

### Review question and eligibility criteria

We aimed to address the following research questions:

How effective are de-implementation initiatives applying discrete or a combination of different de-implementation strategies in reducing LVC in different healthcare practices? *(a)* How effective are specific discrete de-implementation strategies? *(b)* How effective are multifaceted de-implementation strategies in comparison to discrete strategies?

The inclusion and exclusion criteria are detailed in Additional file 1, eTable 1. We included all paediatric and adult patient populations who might receive LVC. The interventions of interest were de-implementation initiatives and discrete strategies (aimed at reducing LVC practices as defined by systematic review [SR] authors or by citing specific guidelines) across all healthcare fields with a comparison (another group, before–after). The outcomes of interest were appropriateness of care use (i.e. these measures also specified whether a certain practice was indicated) and LVC use (i.e. service utilisation proportions) [7]. Outcomes were reported narratively or quantitatively. We included SRs as defined according to the Cochrane Handbook [17].

### Information sources and literature search

An information specialist designed and conducted database searches in MEDLINE (via the database platform Ovid), Epistemonikos.org and Scopus (Elsevier) from 1 January 2010 to 17 April 2023. We restricted the publication year to 2010 since the first guideline to report SR results was published in 2009, and the conduct and reporting quality has assumedly improved since [18]. The MEDLINE search strategy was developed based on a text analysis of relevant SRs, peer reviewed by a second information specialist using the Peer Review of Electronic Search Strategies (PRESS) checklist [19], and adapted to other databases. The searches were limited to English and German language articles (see Additional File 1, eTable 2). We applied further search methods: hand-searching the Cochrane reviews produced by the EPOC group and relevant journals (e.g. *Implementation Science*, *Implementation Science Communication*) and reference checking overviews of reviews and selected articles [7, 20, 21].

### Literature selection

Based on our inclusion and exclusion criteria, two reviewers independently screened titles and abstracts as well as full-text articles of each reference. Any disagreements were resolved by discussion. We listed all excluded full-text articles and the reason for exclusion (Additional file 1, list of excluded studies). We pilot-tested the abstract and full-text review forms with 50 and 10 selected records, respectively. We used the Covidence software (https://www.covidence.org/) for the study selection process.

### Study quality assessment

Two independent reviewers assessed the included SRs’ quality using a revised version of A MeaSurement Tool to Assess systematic Reviews (AMSTAR 2) [22]. We amended AMSTAR 2’s critical flaws definitions. Instead of the seven items originally defined as critical flaws, we used five items (see Additional File1, eTable 3). We considered the justification for excluding individual studies and assessment of publication bias as a minor rather than critical flaw to achieve a less strict risk of bias (RoB) assessment of the included SRs [23]. Based on our assessment of all AMSTAR 2 items, we determined the overall study quality using the categories high, moderate, low, and critically low confidence. Disagreements between reviewers were resolved through discussion or, if necessary, by involving a third reviewer.

### Data extraction

One reviewer extracted data using a standardised piloted data extraction template with predefined items; another checked the extracted data for errors and incompleteness. We extracted information on the SR details (e.g. publication year, number of included studies according to study design, RoB assessment), the de-implementation strategy details (e.g. de-implementation rationale, de-implementation strategies applied as mentioned by authors, healthcare practices and fields), and SR results (e.g. synthesis type, detailed results for meta-analysis [MA] and vote counting for narrative synthesis, and Grading of Recommendations, Assessment, Development and Evaluation [GRADE] assessment) [24]. We extracted only limited data from SRs with a critically low overall confidence in the results (e.g. number of included studies, healthcare practices, search period, setting).

As mentioned above, one reviewer extracted information on de-implementation strategies as reported in the SRs. This information was checked by a second reviewer. Afterwards, one reviewer coded the de-implementation strategies according to the ERIC compilation of strategies [6, 9–11], incorporating additional strategies, if necessary (i.e. accountability tool, Food and Drug Administration black box warning, policy and regulations, communication tool and international collaboration). Again, this mapping process was checked by a second reviewer and disagreements were discussed. We started the coding process at the level of the specific strategies. However, due to their limited description, the differentiating between strategies was sometimes challenging (e.g., ‘conduct educational meetings’ or ‘conduct ongoing training’ or ‘make training dynamic’). Therefore, we refrained from presenting the results on individual strategies and instead relied on the presentation of ERIC clusters [11]. Based on discussions within the review team, we grouped the additional strategies mentioned by Perry et al. [9] and specific de-implementation strategies identified by Ingvarsson et al. [6] to the existing clusters to maintain the aggregated level of synthesis. For example, we categorized the strategy ‘accountability tool’ to the ERIC cluster of strategies *use evaluative and iterative strategies* to highlight the evaluative function of this strategy. The strategy ‘assess and redesign workflow’ was mapped to the ERIC cluster *change infrastructure*, as other strategies have already highlighted changes within organisational processes. To highlight these changes, we renamed the cluster to *change infrastructure and workflow*. Further categorization details can be found in Additional File 1, eTable 4, which outlines the coding of strategies.

### Data synthesis and analysis

Using a narrative synthesis approach [15], we present the SRs’ results in the text structured according to healthcare practices and consider the SR quality (expressed as confidence levels) as the most important aspect as well as the number of included studies. Additionally, we describe the results of de-implementation strategies within and across healthcare practices. We used summary statistics (i.e. frequencies and proportions) to describe SR characteristics.

We used harvest plots [25] to visualise the effectiveness of the de-implementation initiatives as reported in the included SRs, structured according to healthcare practices (e.g. antibiotic treatments, opioids, laboratory tests). Furthermore, we displayed the effectiveness of discrete de-implementation strategies applied in comparison to multifaceted de-implementation strategies. Each SR is represented as a bar positioned on a matrix, depicting the confidence in the results (y-axis) and the overall effectiveness expressed as a positive change (i.e. reduction of LVC practices), inconsistent positive change or no change (x-axis). The applied decision rules, discussed and finalised between two reviewers, are depicted in Table 1.

**Table 1:** Rules for SR assessments for harvest plot analysis

**Rule no.**	**Rules as applied for general harvest plots**
1	We analysed the SR results separately for the ‘appropriateness of care use (short: appropriateness)’ and ‘low-value care use (utilisation)’ outcomes.
2	If a review contained MA and narrative results, the MA results were prioritised.
3	We categorised the outcome as ‘positive effect’, ‘inconsistent positive effect’, and ‘no change’. A positive effect was coded if the results of a MA were statistically significantly positive or if > 75% of the primary studies provided a statistically significantly positive effect (vote counting). An inconsistent positive effect was coded if 50 to 75% of the primary studies reached a statistically significant result. No change was coded if ≤ 50% of the primary studies reached a statistically significant result.
4	If a review reported more than one MA result of outcomes comprised in one outcome category, and if the results were conflicting, we considered the number of included studies in each MA, and the results were categorised as above.
5	If a review reported no overall MA but subgroup results (e.g. different study designs, different intervention categories), and if the results were conflicting, the results were categorised as above.
**Rule no.**	**Rules applied for harvest plots for discrete de-implementation strategies and the comparison of discrete and multifaceted strategies**
6	In addition to the abovementioned rules, we applied the following: For these analyses, we considered only utilisation outcomes.
7	Harvest plot for discrete/multifaceted strategies: We only considered SRs as relevant for this analysis if they reported on both the effectiveness of discrete and multifaceted strategies.
8	Harvest plot for specific discrete strategies: A SR needed to report the de-implementation strategy results separately (number of studies revealing statistically significant effects) to be included in the analysis. We categorised the specifically mentioned strategies into ERIC clusters. If a SR reported on more than one strategy in the same ERIC cluster, the results were combined.

### Deviations from the protocol

We initially planned to extract and synthesise data on the safety of de-implementation initiatives but, due to limited time resources, we decided to record this information only if these outcomes were reported in the SR. During the full-text pilot screening process, we added the specification to the exclusion criteria that we would not include SRs on the topic of de-prescribing, as it differs contextually from preventing the initiation of a treatment (de-implementation) [7]. Rather than assessing the included SRs’ study quality with the Risk of Bias in Systematic Reviews (ROBIS) tool [26], we used the more up-to-date AMSTAR 2 tool. We additionally extracted information on the GRADE assessment [24].

## Results

### Search results

**Fig. 1:**
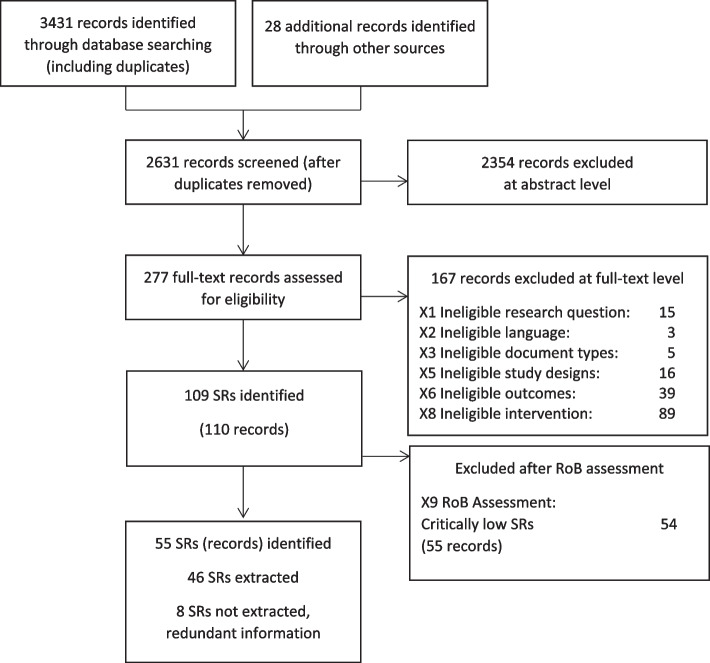
PRISMA Flow diagram

We identified 2631 records after de-duplication stemming from the database search (*n* = 2603) and searches of other sources (*n* = 28). After abstract screening, we assessed 277 full texts and identified 109 SRs (110 articles) meeting our predefined eligibility criteria. We excluded 54 (55 articles) after evaluating their quality as having critically low confidence in their reported results. A description of the excluded SRs is listed in Additional file 1, eTable 5 and eTable 6. To minimise overlap in the included SRs, we did not extract data from a SR if the included primary studies were also included in other SRs (*n* = 3) or if other included SRs provided more up-to-date information (*n* = 5) (Additional file 1, eTable 7). Further SRs did not exactly address the same research question applying the same eligibility criteria. Finally, we included 46 SRs for data synthesis and analysis. Figure 1 shows the details of the study selection process, and Table 2 provides an overview of the included SRs.

**Table 2:** Overview of included SRs

Author Year, [Ref]	Confidence level	Topic	Outcomes	
**Drug treatment: antibiotics**				
Coxeter 2015 [27]	MOD	Shared decision-making for ARI in primary care	Utilisation^a^	
Davey 2017 [28]	HIGH	Improvement of hospital prescribing practice	Appr. ^a^	Utilis. ^a^
deBont 2015 [29]	LOW	Patient information leaflets in general practice	Utilisation^b^	
Doan 2014 [30]	HIGH	Rapid viral diagnosis in ED	Utilisation^c^	
Fleming 2013 [31]	LOW	Reduce inappropriate prescribing in long-term care	Appr. ^c^	Utilis. ^c^
Lane 2018 [32]	LOW	Use of epidemiological data in primary care	Utilisation^a^	
Lim 2020 [33]	LOW	National interventions for responsible usage	Utilisation^a^	
Martinez-Gonzalez 2020 [34]	HIGH	Point-of-Care C-Reactive protein testing in primary care	Utilisation^a^	
Mortazhejri 2020 [35]	HIGH	Patient-oriented interventions for URTI	Utilisation^a^	
Nabovati 2021 [36]	LOW	Information technology interventions for ARI	Utilisation^b^	
Nair 2021 [37]	MOD	Outpatient behavioural intervention in LIC & LMIC	Utilisation^a^	
Nguyen 2019 [38]	MOD	Antimicrobial stewardship in care homes	Appr.^c^	Utilis. ^c^
O’Sullivan 2016 [39]	HIGH	Written information for URTI patients	Utilisation^a^	
Raban 2023 [40]	LOW	Nudge interventions in primary care	Utilisation^a^	
Rajar 2020 [41]	MOD	Antibiotic stewardship in premature infants	Utilisation^a^	
Siachalinga 2022 [42]	LOW	Antimicrobial stewardship for hospital patients	Appr. ^a^	Utilis.^b^
Smedemark 2022 [43]	MOD	Point-of-care-tests in ARI patients in primary care	Utilisation^a^	
Spurling 2017 [44]	HIGH	Delayed antibiotic prescriptions for RTI	Utilisation^b^	
VanDijck 2018 [45]	MOD	Hospital antibiotic stewardship interventions in LIC & LMIC	Utilisation^b^	
Vodicka 2013 [46]	LOW	Reduce antibiotic prescribing in children with RTI	Utilisation^b^	
**Drug treatment: opioids**				
Badreldin 2023 [47]	LOW	Reduce opioid prescribing in postpartum patients	Utilisation^a^	
Daoust 2022 [48]	MOD	Reduce opioid prescribing after ED	Utilisation^a^	
Phinn 2023 [49]	MOD	Organisational interventions on discharge	Utilisation^a^	
Zhang 2020 [50]	MOD	Behavioural interventions post-surgery	Utilisation^a^	
**Drug treatment: antipsychotics**				
Birkenhäger-Gillesse 2018 [51]	LOW	Psychosocial interventions for BPSD	Utilisation^b^	
Mokhar 2018 [52]	LOW	Patient-centred care interventions to reduce inappropriate medication	Utilisation^a^	
ThompsonCoon 2014 [53]	MOD	Reduce inappropriate prescribing in people with dementia resident in care homes	Utilisation^c^	
**Laboratory tests**				
Dunn 2021 [54]	LOW	Clinical Decision Support Alerts on CD Testing	Appr. ^a^	Utilis.^b^
Kobewka 2015 [55]	MOD	Reduce laboratory test utilisation	Utilisation^b^	
Yeshoua 2023 [56]	LOW	Reduce repetitive inpatient test ordering	Utilisation^b^	
Zare 2021 [57]	LOW	CDSS to reduce inpatient test ordering	Utilisation^b^	
Zhelev 2016 [58]	MOD	Reduce thyroid function tests	Appr. ^c^	Utilis.^b^
**Diagnostic imaging**				
Belavy 2022 [59]	MOD	Reduce low-value imaging for low back pain	Utilisation	
Dunne 2022 [60]	MOD	Reduce computed tomography in ED	Utilisation^b^	
Kjelle 2021 [61]	LOW	Reduce low-value imaging	Utilisation^a^	
Zare 2022 [62]	LOW	CDSS for appropriate use of imaging	Appr. ^a^	Utilis. ^a^
**Other tests**				
Foster 2020 [63]	LOW	Audit and feedback for ordering in critical care	Appr.^b^	Utilis.^b^
Takada 2020 [64]	LOW	Reduce low-value medical tests in primary care	Utilisation^b^	
Xie 2022 [65]	MOD	Reduce medication use and test ordering	Utilisation^c^	
**Other interventions**				
Baptista 2018 [66]	LOW	Decision aids for prostate cancer screening	Utilisation^c^	
Chen 2018 [67]	HIGH	Reduce unnecessary caesarean sections	Utilisation^c^	
Ralston 2014 [68]	LOW	Reduce hospitalisation for bronchiolitis	Utilisation^a^	
Rietbergen 2020 [69]	MOD	Reduce low-value nursing procedures	Utilisation^c^	
Sypes 2020 [70]	LOW	Engage patients to reduce clinical care	Utilisation^a^	
Xiong 2018 [71]	MOD	Reduce unnecessary central venous catheter use	Utilisation^c^	
Xu 2021 [72]	LOW	Reduce SUPP in Intensive Care Units	Appropriat.^b^	

*Abbreviations*: *Appr.* appropriateness, *ARI *acute respiratory infections, *BPSD *behavioural and psychological symptoms in dementia, *CD *Clostridioides difficile, *CDSS *clinical decision support systems, *ED *emergency department, *LIC *low-income countries, *LMIC *low- and middle-income countries, *RTI *respiratory tract infection, *SUPP *stress ulcer prophylaxis pharmacotherapy, *URTI *upper respiratory tract infections *Utilis*. utilisation

^a^﻿statistically significant reduction

^b^inconsistent reduction

^c^no change

### SR characteristics

**Fig. 2:**
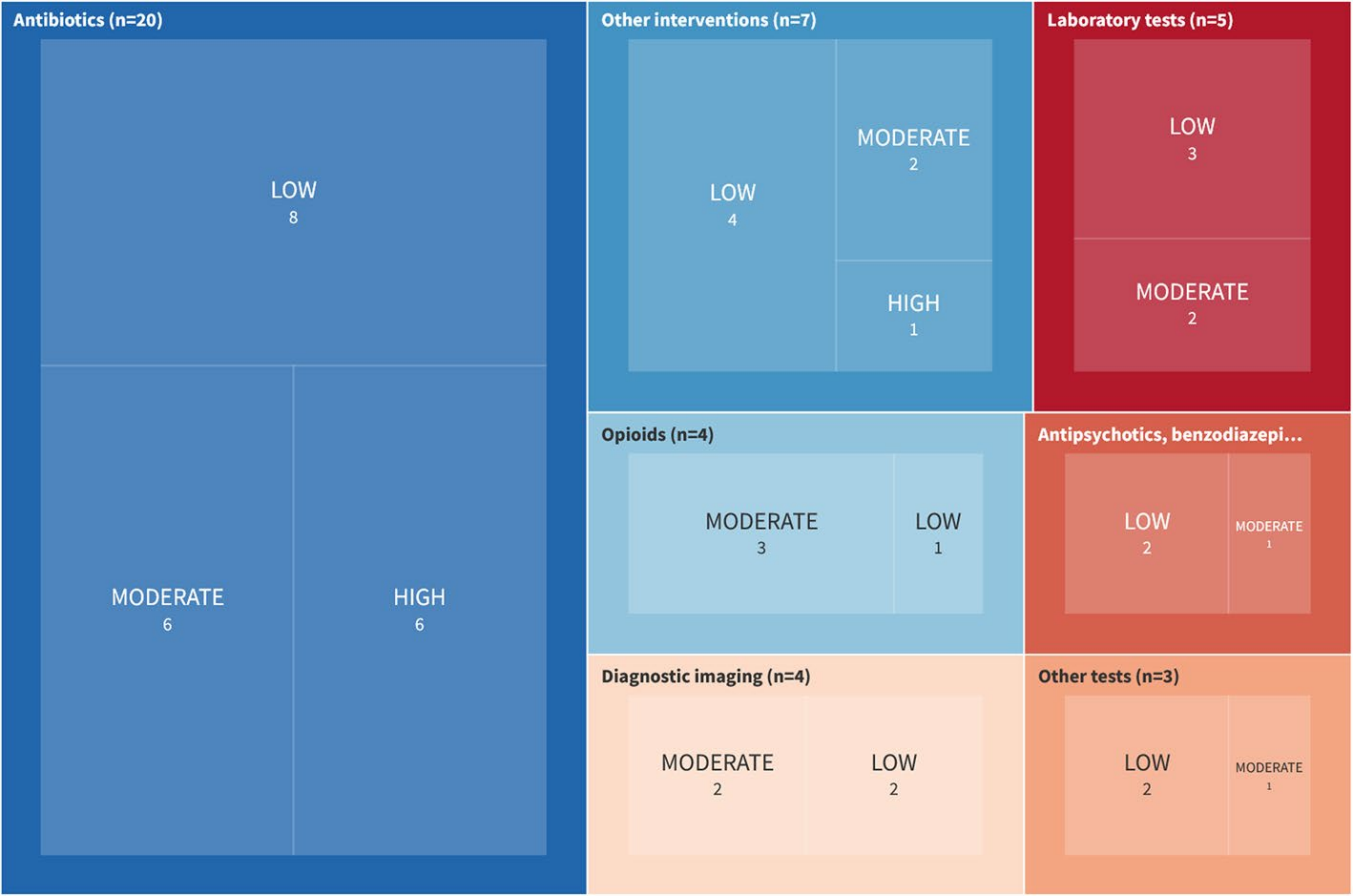
Confidence assessments of the included SRs (see also reference [73]  to explore the online figure)

The 46 included SRs spanned seven distinct healthcare practices: ‘Drug treatments: antibiotics’ (*n* = 20), ‘Laboratory tests’ (*n* = 5), ‘Drug treatments: opioids’ (*n* = 4), ‘Diagnostic imaging’ (*n* = 4), ‘Drug treatments: antipsychotics, benzodiazepines’ (*n* = 3), ‘Mixed diagnostic tests’ (*n* = 3), and ‘Other interventions’ (e.g. utilisation of caesarean sections, central venous catheters [CVCs] or stress ulcer prophylaxis pharmacotherapy, *n* = 7) (see Fig. 2). Among the 46 SRs, 32 synthesised the results narratively; 14 provided at least one MA result. The confidence levels in the SR results varied, with the majority rated as low confidence (*n* = 22), followed by moderate (*n* = 17) and high confidence (*n* = 7). The major reasons for downgrading were: no statement that an a-priori protocol existed (*n* = 12), and the SR did not account for the included primary studies’ RoB when interpreting the results. For a more detailed description, see Additional File 1, eTable3. SRs with high confidence ratings were predominantly found in the ‘Drug treatments: antibiotics’ category (*n* = 6). The certainty of evidence was assessed in seven SRs. The GRADE ratings of the selected outcomes and SRs are listed in Additional File 3.

The primary studies included in the SRs were published between 1974 and 2022. The most extensive timeframe is observed in the ‘Laboratory tests’ category, covering 47 years (1974–2021), followed closely by the ‘Drug treatments: antibiotics’ category, ranging 46 years (1976–2022). The number of included primary studies in the SRs ranged from two to 221 (see Additional File 1_eTable8).

The included SRs encompassed a variety of settings; most focused on secondary/tertiary care (*n* = 17) (see Table 3). The primary care setting was prevalent in ‘Drug treatments: antibiotics’ (11/12 SRs). Among all the SRs, family medicine was the most frequently represented medical field (*n* = 10). The ‘Other interventions’ category contained SRs addressing multiple specialties or SRs with missing information (n = 22). Approximately 50% of the included primary studies were conducted in North America and 26% in Europe, with variations across healthcare practices. The primary de-implementation rationale was ‘Evidence suggests more harms than benefits for the patient or community’ (n = 36). The SRs described the study aims primarily as ‘reducing LVC’ (n = 43) (see Additional File 1_eTable 9).

**Table 3:** Characteristics of the included SRs

SR characteristics	*n*	De-implementation initiative details	*n*	Incl. primary study characteristics	%
***Setting***		***Taxonomies applied***		***Geographic region***	%
Primary care (c.)	12	EPOC	6	North America	50
Secondary/tertiary c.	17	Michie et al.	1	Europe	26
Nursing/long-term c.	2	EPOC and Michie et al.	3	Other	24
Prim., sec./tert. c.	15	No taxonomy used	8		
		Inclusion criteria	14	***Intervention type***	%
***Medical field***		Self-developed	11	Single	56
Anaesthesiology	1	Additional tools used	2	Multifaceted	44
Diagnostic radiology	1				
Emergency medicine	2	***Temporality***			
Family medicine	10	Reported	10		
Internal Medicine	2	NR	36		
Obstetrics and gynaecology	2				
Paediatrics	2	***Dosage***			
Psychiatry	2	Reported	39		
Surgery	1	NR	7		
Urology	1				
Other	22	***TMF***			
		Reported	1		
***Aim***		NR	45		
Reduce	43				
Replace	2	***Barriers/facilitators***			
Restrict	1	Reported	0		
		NR	46		
***Rationale***					
More harms than benefits	36	***Long-term outcomes***			
Little or no benefit	5	Reported	6		
Another treatment is more effective or less harmful	3	NR	40		
Cost-effectiveness	1	***Targets patients***			
NR or unclear	1	Yes	13		
		No	33		

*Abbreviations*: *EPOC *Effective Practice and Organization of Care, *NR *not reported, *SR *systematic review, *TMF *theories, models, frameworks

### De-implementation initiative characteristics

Taxonomies for categorising de-implementation strategies were seldom applied. The EPOC system was most often used (*n* = 6) [7, 13], followed by Michie et al.’s [8, 12] intervention functions (*n* = 1) and a combination of both taxonomies (*n* = 3). Further, 14 SRs did not apply a taxonomy but specified the investigated de-implementation strategies via the inclusion criteria (e.g. SR focused on ‘audit and provide feedback’). Two SRs used additional tools to categorise de-implementation strategies (see Additional File 1_eTable 10).

**Fig. 3:**
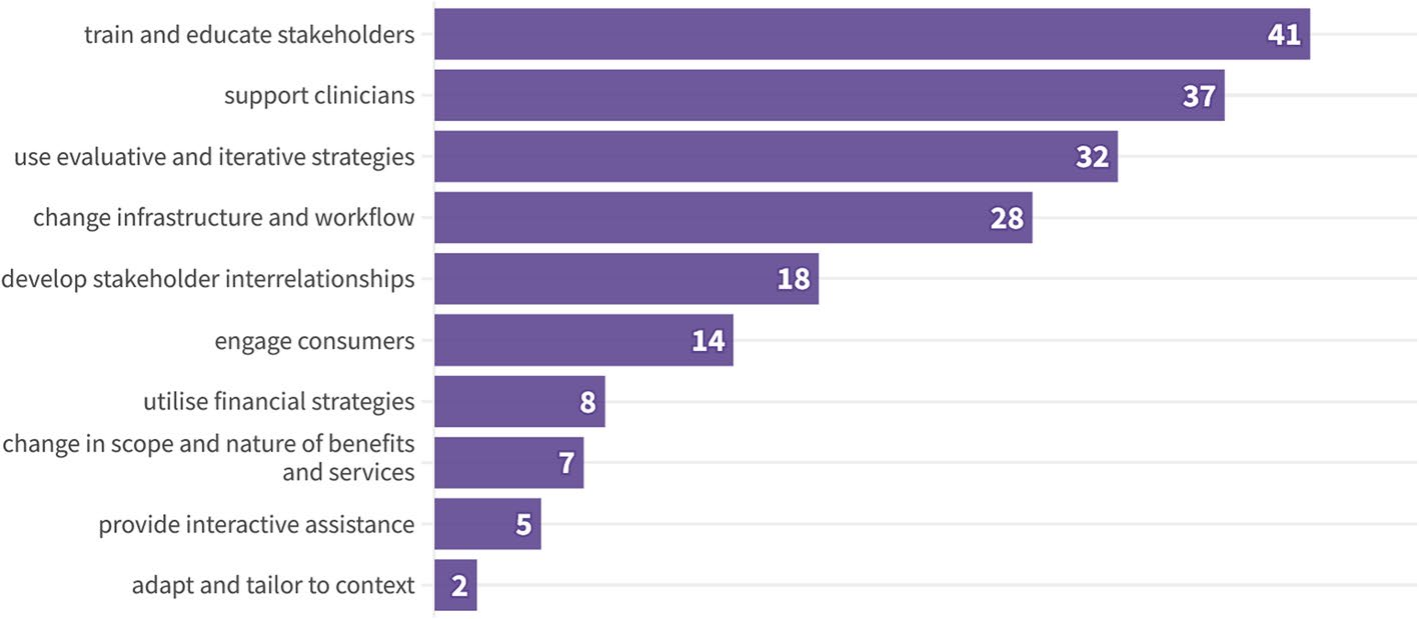
ERIC strategy clusters addressed in SRs (*n* = 46)

Figure 3 depicts the frequency of ERIC strategy clusters resulting from our coding of the strategies included in the SRs. Strategies related to the *train and educate stakeholders* cluster were applied at least once in individual studies in 41 SRs. Other frequently applied strategies reflected the *support clinicians*, *use evaluative and iterative strategies*, and *change infrastructure and workflow* clusters. Notably, the individual SRs examined between one and seven ERIC strategy clusters (median = 4). We identified a category not previously mentioned in the ERIC compilation. *Changes in scope and nature of benefits and services* was used to describe offering dementia patients physical or social exercises to reduce antipsychotics or offering the general population physical therapy to reduce opioid consumption. The ERIC strategy clusters were employed with similar frequency across different healthcare practices (Additional File 1_eFigure 1).

The included SRs seldomly reported details on de-implementation initiatives. Information on temporality (i.e. when was the de-implementation initiative target group addressed) and on duration or intensity (i.e. dosage) was provided in ten and seven SRs, respectively. The initiatives’ development was addressed in one SR, detailing whether the included primary studies reported using guidelines for initiative design and implementation. None of the SRs provided information about tailoring use (i.e. choosing de-implementation strategies based on a contextual assessment of barriers and facilitators). While all SRs reported healthcare providers as the targets of de-implementation initiatives, 13 additionally named patients as targets (see Additional File 1_eTable 11).

### Participant details

The patient population in the included SRs ranged from 1,595 to 2,529,855. Details on age and participants’ (patients or health professionals) gender were often not reported. The observation period ranged from four days to 17 years. Long-term outcomes (> 12 months) were reported in only six SRs (see Additional File 1_eTable 12).

### Effectiveness of de-implementation initiatives according to healthcare practices

#### Drug treatment: antibiotics

**Fig. 4:**
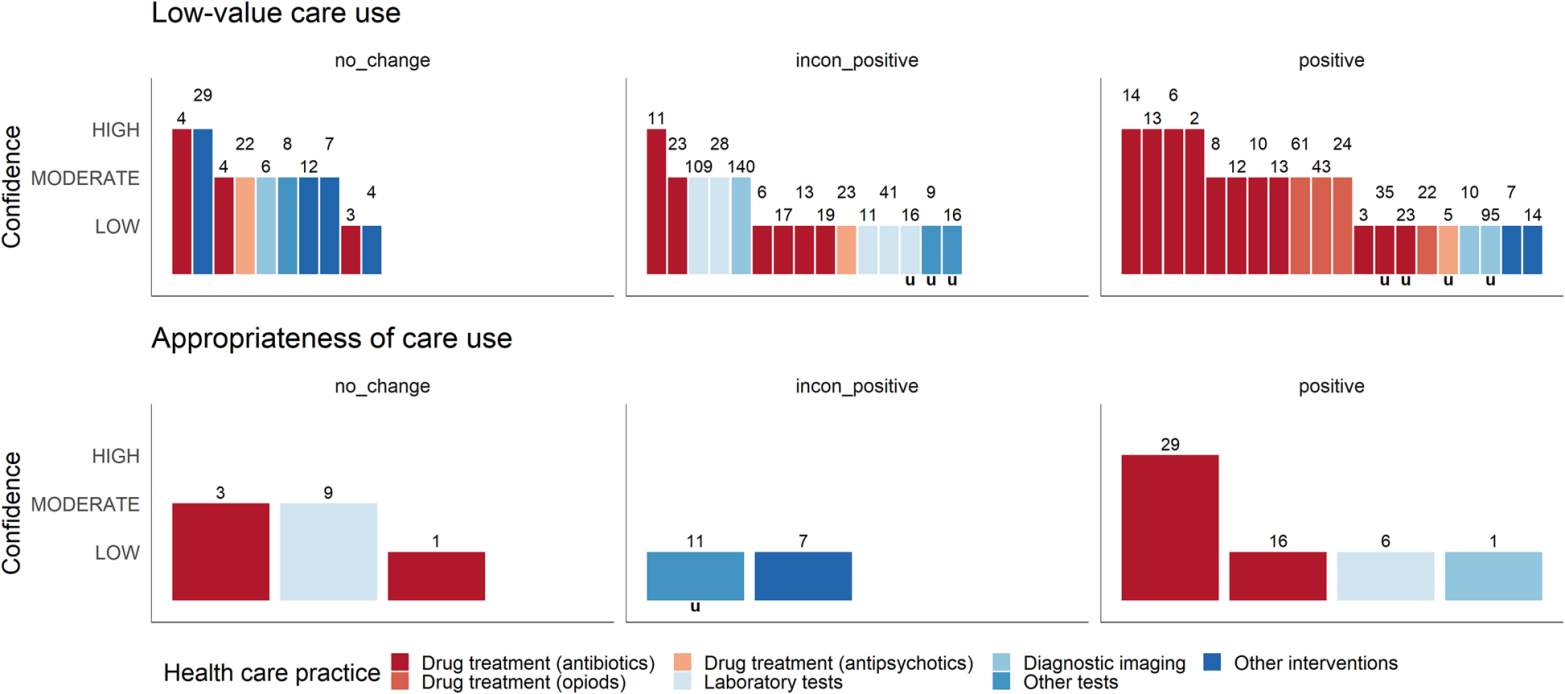
Harvest plot for LVC utilisation and appropriateness (n_SR_=46) **Explanation figure:** This harvest plot represents the ‘low-value care use (utilisation)’ and ‘appropriateness of care use’ outcomes. Each included SR is represented as a bar in the matrix at least once and twice if the SR reported on both outcomes **Bar position:** (see Table 2): Row ‘positive’ (< 75% or MA showing an effect), ‘inconsistent positive’ (< 50–75%), or ‘no change’ (≥ 50% or MA showing no effect) **Bar colour:** Healthcare practice (see legend) **Bar height:** Confidence in the results according to the AMSTAR 2 assessment **Number above the bar:** Number of included primary studies in the SR **Lowercase “u” under a bar:** Relevant information for assessing the primary studies’ statistical significance was not reported in the SRs

We identified 20 SRs investigating the effectiveness of de-implementation initiatives aimed at curtailing antibiotic utilisation; four also reported on the reduction of inappropriate antibiotic prescriptions [28, 31, 38, 42]. Our assessment categorised six of the included SRs as having high confidence in the results [28, 30, 34, 35, 39, 44], six as moderate confidence [27, 37, 38, 41, 43, 45], and eight as low confidence [29, 31–33, 36, 40, 42, 46]. Overall, 11 SRs reported statistically significant positive reductions in antibiotic utilisation [27, 28, 32–35, 37, 39–41, 43]. Five of these studies reported on the certainty of evidence using the GRADE assessment ranging from very low [43], low [27, 39], moderate [27, 39, 43, 44] to high certainty [28] of the evidence For example, a MA of a high-confidence Cochrane review [28] showed a reduction of 1.95 days in antibiotic treatment durations (95% confidence interval [CI]: 2.22 to 1.67; 14 randomised controlled trials [RCTs], high level of certainty). Additionally, six SRs showed inconsistently positive reductions in antibiotic utilisation [29, 36, 42, 44–46], while the remaining three [30, 31, 38] found no statistically significant change in ≥ 50% of the included primary studies) (see Fig. 4). Outcomes pertaining to the appropriateness of antibiotic prescriptions were less frequently explored. Two SRs reported statistically significant positive results [28, 42], and two indicated no change in the appropriateness of antibiotic prescriptions [31, 38].

Thirteen SRs provided data regarding the effectiveness of seven distinct ERIC strategy clusters, presented in detail below [27, 29, 30, 32–37, 39, 43–45].

#### Change infrastructure and workflow

This cluster comprised two discrete de-implementation strategies: delayed versus immediate prescription categorised in ‘assess and redesign workflow’ and the application of point-of-care tests categorised in ‘change physical structure and equipment’. Two MAs showed a reduction in antibiotic utilisation for respiratory infections for delayed versus immediate prescriptions (odds ratio [OR]: 0.04, 95% CI: 0.03 to 0.05 [44] and OR: 0.09, 95% CI: 0.03 to 0.23, respectively [35]). This effect diminished when delayed prescription was compared to no antibiotics [44]. The application of different point-of-care tests, such as the c-reactive protein and procalcitonin test, reduced antibiotic utilisation in URTI treatment in two MAs (c-reactive protein: risk ratio [RR]: 0.79, 95% CI: 0.70 to 0.90, 13 RCTs [39] and RR: 0.77, 95% CI: 0.69 to 0.86, 12 RCTs, and procalcitonin measurements: RR: 0.32, 95% CI: 0.23 to 0.44, 1 RCT) [35] and in one SR’s narrative synthesis [29]. Supplementary tests, including nasopharyngeal swabs, contributed to a non-statistically significant reduction in prescribed antibiotics (RR: 0.89, 95% CI: 0.71 to 1.12) [46].

#### Support clinicians

Three SRs examining the efficacy of computer-supported initiatives in primary care settings (‘remind clinicians’) found inconsistent positive reductions. The implementation of CDSS revealed statistically significant reductions in one moderate-level confidence SR [37]. A less consistent effect was shown in a narrative SR (9/16 studies) [38]. Providing locally relevant real-time infection epidemiological data to clinicians contributed to a decrease in antibiotic utilisation (3/3 studies) [32].

#### Engage consumers

Five SRs providing evidence on strategies comprised in the cluster *engage consumers* demonstrated a reduction, albeit inconsistent, in both antibiotic prescriptions and consumption. In a moderate-confidence MA [27], shared decision-making (‘communication tool’) led to a reduction in antibiotic use compared to usual care (RR: 0.61, 95% CI: 0.55 to 0.68, 8 RCTs). The evidence base is inconclusive if the provision of written information, online interactive sessions or mass media strategies for patients (‘prepare patients/consumers to be active participants’), compared to usual care, reduces antibiotic utilisation [29, 33, 35, 39]. Another low-confidence SR [46] synthesised the effectiveness of initiatives based on their target groups. The findings indicated that initiatives directed at both clinicians and patients (e.g. parents), as well as those exclusively targeting patients, are more often effective (8/10 and 2/3 studies, respectively) than initiatives solely focussing on clinicians (3/6 studies).

#### Other categories of strategies

Using *evaluative and iterative strategies* (‘audit and provide feedback’*)* reduced antibiotic utilisation (3/4 studies) [45]. The evidence base regarding *train and educate stakeholders* [33, 37, 45], *utilise financial strategies* (e.g. restrictions on reimbursement of antibiotic purchases), and *develop stakeholder interrelationships* [33] is inconclusive.

### Drug treatment: opioids

Three moderate-confidence SRs [48–50] and one low-confidence SR [47] pertinent to the reduction in opioid utilisation were identified. The most comprehensive SR [48] with 63 studies showed significant reductions in prescription rate and opioid prescription quantity in patients discharged from the emergency department (interrupted time series [ITS]: standard effect [SE]: -22.61% [95% CI: -30.70% to -14.51%]; ITS: SE: -8.64% [95% CI: -17.48% to 0.20, respectively]). Two other moderate-confidence SRs [49] focussing on organisational initiatives to reduce opioid prescribing for non-cancer pain on hospital discharge and behavioural initiatives to decrease opioid prescribing after surgery [50] mirror these results. A low-confidence SR [47] highlighted that initiatives were effective in reducing inpatient and discharge opioid prescribing for postpartum patients.

### Results specific to ERIC strategy clusters

Three SRs [48–50] provided evidence regarding six ERIC strategy clusters. *Develop stakeholder interrelationships* (in particular, use ‘advisory boards and workgroups’ and ‘conduct local consensus discussions’) resulted in statistically significant opioid prescription reductions [48–50]. Furthermore, the strategy clusters *use evaluative and iterative strategies* (i.e. ‘audit and provide feedback’) [48] and *engage consumers* led to opioid prescription reductions. While ‘educational interventions’ targeting healthcare providers alone resulted in inconsistent positive reductions of opioid prescriptions [49], the combination of ‘educational interventions’ and the ‘distribution of guidelines’ proved to be effective [48, 50]. The evidence concerning the effectiveness of changes to default quantities in ‘electronic medical records’ (*change infrastructure and workflow*) was inconclusive [48–50]. Using physical therapy for pain reduction (*changes in scope and nature of benefits and services*) resulted in no statistically significant change in reducing opioids [48].

### Drug treatment: antipsychotics, antidepressants, benzodiazepines

One moderate-confidence SR [53], along with two low-confidence SRs [51, 52], reported on the effectiveness of initiatives aimed at reducing the utilisation of psychoactive drugs. These SRs yielded positive results; however, these findings were not consistently favourable. Psychosocial interventions on managing behavioural and psychological symptoms in dementia led to a reduction in the proportion of patients utilising antipsychotic medication (RR 0.71, 95% CI: 0.59 to 0.88, 9 RCTs) but not antidepressants, when compared to the usual care group [51]. Only an inconsistent reduction in antipsychotic medication utilisation was revealed in care home residents diagnosed with dementia (8/22 studies) [53]. Conversely, a consistent decrease in benzodiazepine and Z-drug utilisation was found across all five included studies [52].

### Results specific to ERIC strategy clusters

Two SRs [51, 53] provided evidence regarding four ERIC strategy clusters. Initiatives aiming at a sustainable *cultural and workflow change* as defined by the primary studies’ authors reduced antipsychotic utilisation in dementia patients (RR: 0.65, 95% CI: 0.57 to 0.73, 6 studies) [51]. Other strategy clusters, such as *train and educate stakeholders* [51, 53], *use evaluative and iterative strategies* (mainly ‘audit and provide feedback’) [53], and *changes in scope and nature of benefits and services* (offering social and exercise interventions for dementia patients) [51] were not shown to be effective.

### Laboratory tests

We identified two moderate-confidence SRs [55, 58] and three low-confidence SRs [54, 56, 57] on the effectiveness of initiatives to reduce the use of either specific (e.g. thyroid function tests) or unrestricted laboratory tests (e.g. various blood tests, urine and stool cultures, pap smear tests). The inpatient hospital [54, 56, 57] and primary care settings were targeted [55, 58]. All reviews reported on test utilisation and two also on test utilisation appropriateness [54, 58].

Overall, the included SRs revealed an inconsistent reduction in low-value laboratory tests. The most comprehensive SR with 109 studies revealed a median relative reduction of 22.2% in laboratory test utilisation (interquartile range [IQR]: 10.1–36.7%) in the primary care setting [55]. An inconsistent reduction (16/27 studies) was found in thyroid function test ordering in primary care [58]. Similarly, in three low-confidence SRs, inconsistent reductions in the utilisation of daily complete blood count and metabolic tests (26/41 studies) [58], laboratory testing applying CDSS (10/16 ) [56] and Clostridioides difficile testing in addition to CDSS were also shown in the hospital setting (6/11) [57].

### Results specific to ERIC strategy clusters

Four SRs [54–57] provided evidence on four different ERIC strategy clusters. *Evaluative and iterative strategies* (‘audit and provide feedback’) resulted in a median relative reduction of 23.2% in laboratory test utilisation (IQR: 13.8 to 34.5) and showed statistically significant reductions in 24 of 41 studies (59%) [55]. Yeshoua et al.’s review [56] reported that the ‘electronical medical record change’, ‘audit and provide feedback’, and ‘cost display’ strategies (*evaluative and iterative strategies*) reduced laboratory test utilisation in 39 of 41 studies (95%), yet, the results’ statistical significance was not reported. ‘Applying CDSS’ subsumed under the *support clinicians* cluster led to statistically significant reductions in more than 50% of the studies in two SRs (6/11, 55% [54], with a median relative reduction of 14.6% [IQR: 3.95 to 28.35; 10/16, 63%] [57]). *Infrastructure and workflow changes* enabled a median relative reduction of 19.6% (IQR: 10.4–36.1%) and a statistically significant reduction in 36 out of 54 studies (67%) [55]. These results were corroborated by another SR (9/9 studies, 100%) missing statistical significance reporting [56]. *Train and educate stakeholders* contributed to a relative reduction of 31.2% in laboratory test utilisation (IQR: 18.1–47.5%; 33/51 [65%]) [55]. These reductions were not observed in another review [56].

### Diagnostic imaging

We identified two moderate- [59, 60] and two low-confidence SRs [61, 62] reporting on outcome utilisation, with one [62] also addressing appropriateness. Among others, the diagnostic imaging procedures considered for de-implementation were transthoracic echocardiography, computed tomography (CT), X-ray, and magnetic resonance imaging (MRI) focussing on specific indications such as low back pain, or the general utilisation of imaging procedures.

Overall, the four included SRs disclosed conflicting evidence, albeit showing a trend towards reduction. A MA revealed evidence of no effect in improving guideline-recommended imaging referrals for low back pain versus no intervention or a passive dissemination of guidelines (OR: 0.87,95% CI: 0.72 to 1.05; 6 RCTs; low certainty evidence) [59]. In contrast, CT scan utilisation in the emergency department was reduced (88/140; 63%; very low certainty evidence) [60]. These results are mirrored by a comprehensive SR with 95 studies focussing on low-value imaging in primary, secondary and tertiary care [61], and by a SR focussing on the reduction of imaging for central nervous system injuries by applying CDSS [62].

### Results specific to ERIC strategy clusters

Three SRs [60–62] reported on all ERIC strategy clusters. *Evaluative and iterative* strategies (e.g. ‘audit and provide feedback’, ‘develop and organise quality monitoring systems’ and ‘accountability tools’) reduced inappropriate diagnostic imaging utilisation as evidenced in two SRs (10/13, 77% [60]; and 24/37, 65% [61] with unreported significance levels). Applying ‘data warehouse techniques’ to implement health information exchange (cluster: *adapt and tailor to context*) reduced diagnostic imaging utilisation as shown by two SRs (4/5, 80% [60] and 5/5, 100% with unreported significance levels [61]). *Change infrastructure and workflow* (e.g. ‘assess and redesign workflows’, ‘change physical structure and equipment’) curtailed diagnostic imaging utilisation (25/30, 83%) [60]. Offering an alternate test to CT in the emergency department (*changes in scope and nature of benefits and services*) resulted in a statistically significant reduction in its utilisation (4/4, 100%, range absolute reductions: 3.9–43.2%) [60].

Overall, we identified conflicting evidence concerning the ‘application of CDSS’ categorised under *support clinicians*. Implementing CDSS effectively reduced the imaging utilisation for brain injuries (OR: 0.82, 95% CI: 0.79 to 0.85, 5 studies) [62] and resulted in curtailing low-value imaging in primary, secondary and tertiary care (7/12, 58%) [61]. This strategy seldomly led to changes in CT utilisation in the emergency department (10/23, 43%) [60]. Three other strategy clusters were not proven to be effective: *train and educate stakeholders* [60, 61] (5/14, 36% and 1/5, 20%), *utilise financial strategies* (0/2) [61], and *engage consumers* (0/1) [61].

### Other tests (imaging, laboratory tests, physiological tests)

Under this section, we describe the results of three SRs investigating multiple LVC practices, such as imaging, testing or transfusion ordering, within one SR [63–65], which prevents them from being assigned to a distinct LVC practice. One moderate-confidence SR [65] yielded that the utilisation of clinical dashboards (*use evaluative and iterative strategies*) compared to usual care had limited effectiveness in reducing laboratory test utilisation and medication prescriptions (3/8, 38%). One low-confidence SR [63] found that multifaceted strategies including an ‘audit and provide feedback’ component (*use evaluative and iterative strategies*) inconsistently reduced laboratory test utilisation or transfusion ordering in critical care (5/9, 56%). These results are mirrored by a low-confidence SR [64], which revealed a median relative reduction of 17% (IQR: 12–24%, *n* = 8) in low-value medical test utilisation (e.g. x-rays or laboratory tests) in primary care. De-implementation initiatives applying ‘reminders for clinicians’ (*support clinicians*) (*n* = 4) or ‘audit and provide feedback’ (*n* = 7) showed larger median relative reductions than initiatives without ‘reminders for clinicians’ (*n* = 6) or without ‘audit and provide feedback’ components (*n* = 3) [64]. The included SRs did not provide further specific results for ERIC strategy clusters.

#### Other interventions

Seven SRs evaluated the efficacy of diverse de-implementation initiatives unable to be categorised into previously defined groups, as these SRs report on distinct LVC practices or target groups. Sypes et al.’s SR [70] demonstrated that de-implementation interventions involving consumer–physician interactions were notably effective (RR 0.74; 95% CI: 0.66 to 0.84) in different LVC practices, such as medication prescriptions, laboratory tests and surgery, compared to no intervention. Further, a SR [68] reported on the reduced utilisation of bronchodilators (risk difference [RD] 0.16, 95% CI: 0.11 to 0.21). Another SR [72] highlighted a reduction in the incidence of inappropriate stress ulcer prophylaxis pharmacotherapy in five out of seven studies (71%), demonstrating a median absolute reduction of 40.2% between pre- and post-intervention (IQR: 71.9–30.8%).

Inconclusive evidence exists regarding the de-implementation of LVC practices such as caesarean sections, central venous catheters (CVCs) or prostate cancer screening. The reduction in medically unnecessary caesarean sections was effective in 10 out of the 29 (34%) included primary studies [67]. The utilisation of CVCs was reported to be reduced in seven out of 14 studies (50%) [71]. A moderate-confidence SR [69] investigated the effectiveness of initiatives to reduce low-value nursing procedures and showed a non-statistically significant reduction in physical restraint use and antipsychotic prescribing in 12 included studies (RR: 0.95, 95% CI: 0.8 to 1.13). The implementation of decision aids for prostate cancer screening did not result in significant changes in screening participation compared to no intervention [66]. The included SRs did not provide further specific results for ERIC strategy clusters.

### Effectiveness of de-implementation strategies defined as ERIC strategy clusters

Of the included SRs, data from 28 SRs [27, 29, 30, 32–37, 39, 43–45, 48–51, 53–57, 60–63, 65, 66] were used for the specific analysis of de-implementation strategies. Information from six SRs [28, 40, 52, 64, 67, 68] could not be synthesised because the authors’ categorisation scheme was incompatible with the ERIC compilation, or the necessary data to assess the results’ statistical significance were inaccessible. Twelve SRs did not provide data on specific de-implementation strategies.

**Fig. 5:**
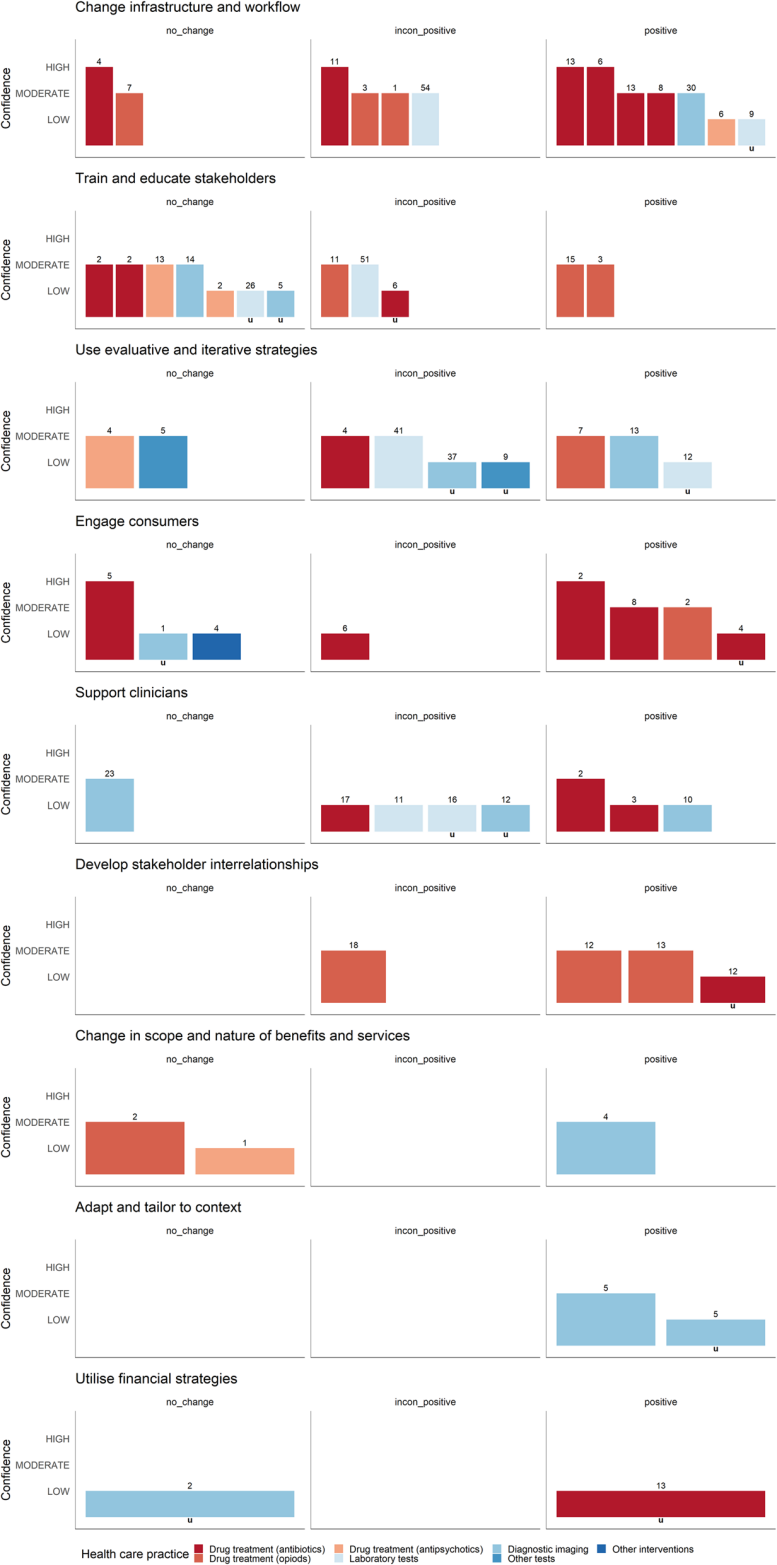
Harvest plots for ERIC strategy clusters (n_SR_=28) **Explanation figure:** This harvest plots represents the ‘low-value care use (utilisation)’ outcome **Bar position:** (see Table 2): Row ‘positive’ (< 75% or MA showing an effect), ‘inconsistent positive’ (< 50–75%) or ‘no change’ (≥ 50% or MA showing no effect) **Bar colour:** Healthcare practice (see legend) **Bar height:** Confidence in the results according to the AMSTAR 2 assessment **Number above the bar:** Number of included primary studies in the SR **Lowercase ‘u’ under a bar:** Relevant information for assessing the primary studies’ statistical significance was not reported in the SRs

Data were most frequently available for the *change infrastructure and workflow*, *train and educate stakeholders*, and *use evaluative and iterative strategies* clusters (see Fig. 5). Strategies included in the *adapt and tailor to context*, *develop stakeholder interrelationships*, and *change infrastructure and workflow* clusters led to consistent reductions in different LVC practices in 100% (2/2), 75% (3/4) and 69% (9/13) of the included SRs, respectively. When also considering inconsistent positive reductions (50% to < 75% of the included studies were statistically significant), all ERIC strategy clusters except utilise *financial strategies* (1/2), *train and educate stakeholders* (6/12), and *changes in scope and nature of benefits and services* (2/3) enabled the reduction of LVC practices.

#### Effectiveness of discrete versus multifaceted strategies

**Fig. 6:**
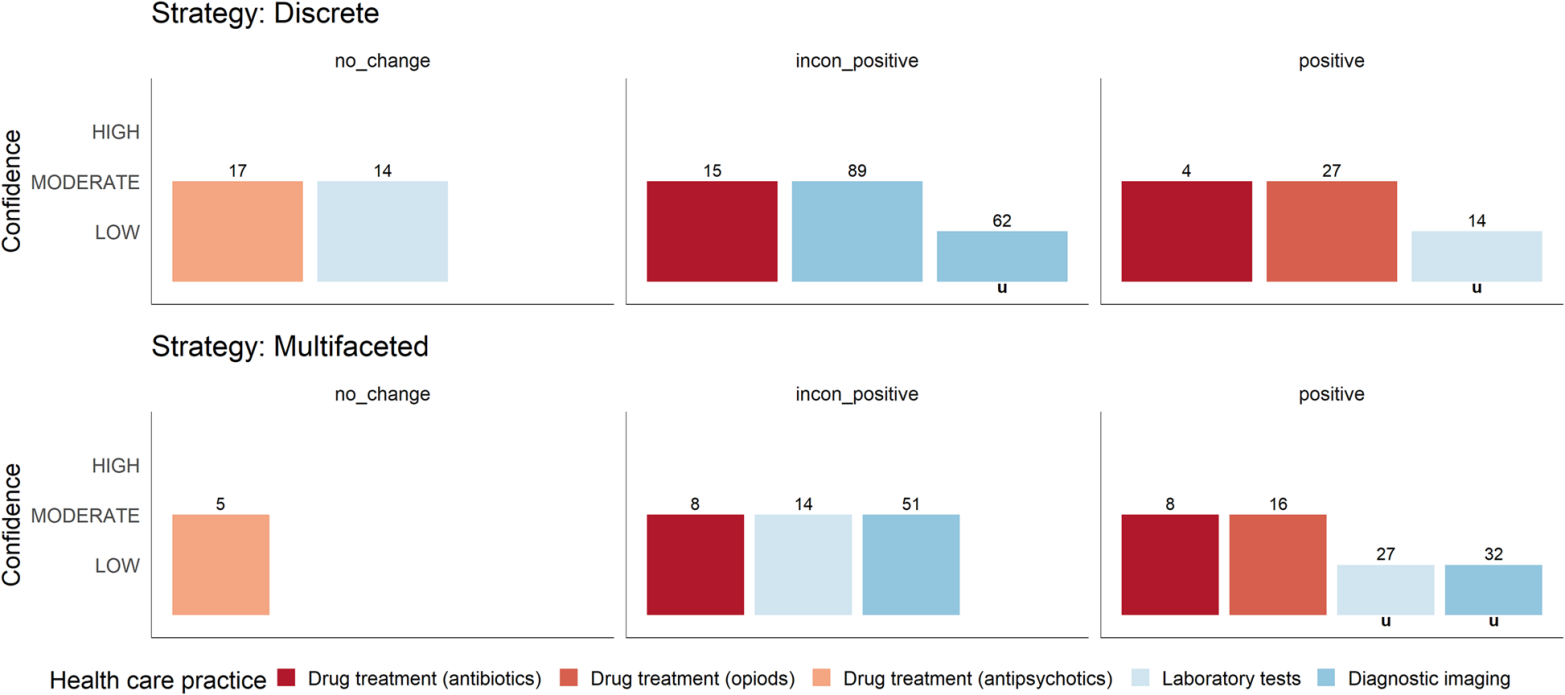
Harvest plot for comparison of single versus multifaceted strategies (n_SR_=8) **Explanation figure:**  This harvest plot represents the ‘low-value care use (utilisation)’ outcome **Bar position:**  (see Table 2): Row ‘positive’ (< 75% or MA showing an effect), ‘inconsistent positive’ (< 50–75%) or ‘no change’ (≥ 50% or MA showing no effect) **Bar colour:** Healthcare practice (see legend) **Bar height:** Confidence in the results according to the AMSTAR 2 assessment **Number above the bar:** Number of included primary studies in the SR **Lowercase ‘u’ under a bar:** Relevant information for assessing the primary studies’ statistical significance was not reported in the SRs

**Fig. 7:**
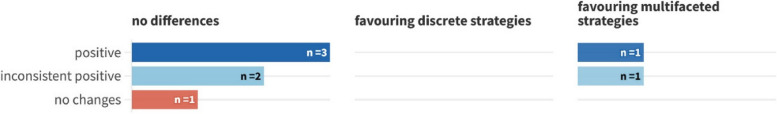
Effectiveness of discrete versus multifaceted strategies (n_SR_=8)

Overall, about 56% of the included primary studies applied discrete strategies. Only eight SRs [37, 45, 49, 53, 56, 58, 60, 61] reported results on comparisons between discrete and multifaceted strategies usable for analysis (Figs. 6 and 7). The included SRs pertained to the following healthcare practices: ‘drug treatment: antibiotics’ (*n* = 2), ‘drug treatment: opioids’ (*n* = 1), ‘drug treatment: antipsychotics’ (*n* = 1), laboratory tests (*n* = 2) and diagnostic imaging (*n* = 2). Within six SRs, the effectiveness was comparable between discrete and multifaceted strategies [37, 45, 49, 53, 56, 60]. Notably, in two SRs, the deployment of multifaceted strategies was more frequently associated with statistically significant positive outcomes [58, 61] than discrete strategies. Additionally, another SR [64] showed that strategies with multiple targets and a combination of strategies were more effective than those with a single target and discrete strategies.

## Discussion

To our best knowledge, this overview of systematic reviews is the first to synthesise the effects of recent SRs describing de-implementation initiatives across several healthcare practices and settings. The 46 included SRs predominantly addressed the reduction of pharmaceutical utilisation and diagnostic procedures, among which antibiotic prescriptions were most frequently examined. Overall, SRs provided evidence for the effectiveness of de-implementation initiatives to reduce antibiotic and opioid utilisation. Reductions in utilisation, albeit not consistent in all the included primary studies, were documented in the utilisation of antipsychotics and benzodiazepines, as well as in laboratory tests and diagnostic imaging. When examining specific de-implementation strategy clusters according to the ERIC compilation, *change infrastructure and workflow*, *adapt and tailor to context* (‘use data warehouse techniques’), and *develop stakeholder interrelationships* consistently reduced LVC practices. A trend towards a positive effect on utilisation, although inconsistent within and across SRs, was identified for all ERIC strategy clusters except *utilise financial strategies*, *train and educate stakeholders*, and *changes in scope and nature of benefits and services*. A trend showed that multifaceted de-implementation initiatives were slightly more favourable than discrete strategies.

 While the focus of the de-implementation literature on reducing medications—particularly antibiotics—laboratory tests and diagnostic imaging were also found in other scoping reviews and in one SR of RCTs [6, 7, 74], we identified SRs also focused on medically unnecessary caesarean Sect. [67], CVC utilisation [71], low-value nursing procedures and decision aids for prostate cancer screening [66]. The most recent SR, published after our search was conducted, demonstrated that over two-thirds of the included RCTs revealed a reduction in LVC practices (75/109 RCTs, 69%) [74]. Contrary to this overview of systematic reviews, no differences between specific de-implementation strategies were identified. This could be explained by diverging outcome presentations. While we applied dichotomous outcomes in our analysis, Heus et al. [74] provided the median relative reductions in LVC use and the IQR.

The results of this overview of systematic reviews revealed that the *change infrastructure and workflow* cluster of strategies reduced LVC practices in nine of 13 SRs. This cluster includes strategies such as ‘change physical structure and equipment’ (e.g. point-of care tests for prescribing antibiotics, opening a dedicated paediatric emergency department or change in record systems) and ‘assess and redesign workflow’ (e.g. case management and care plans, staffing models and staff increase and delayed prescription). Restructuring physical environments seems to make a behaviour change more probable and therefore contributes to LVC practice reduction [75]. From a behavioural change perspective, using ‘point-of-care tests’ to differentiate between a viral or a bacterial infection instead of prescribing antibiotics could also be seen as a behaviour substitution technique as discussed in the behaviour change taxonomy developed by Michie et al. [12]. This would highlight that replacing a treatment/test with a new healthcare practice could be more effective than simply reducing the utilisation of a certain LVC practice [5, 8]. We also recognised the necessity of incorporating an additional strategy into the ERIC compilation, specifically addressing *changes in scope and nature of benefits and services*, which describes replacing a LVC practice with another treatment. This strategy was inductively coded from Thompson Coon et al.’s SR [53] on the effectiveness of interventions to reduce inappropriate prescribing of antipsychotics and stemmed from an earlier version of the EPOC taxonomy [76]. We preferred the terminology of the EPOC taxonomy for two reasons. First, the EPOC taxonomy emphasizes structural changes, which aligns with the inductive coding of the data. Second, the focus of the behaviour change taxonomy on individual behaviour was less suitable for our purposes. Specific results for this discrete strategy were only reported in three SRs. One SR provided evidence for the strategy’s effectiveness in providing information for healthcare providers on alternative imaging procedures for CT in the emergency department [60]. Two other SRs revealed no reductions in LVC practices when offering dementia patients social and exercise interventions instead of prescribing antipsychotics or physical therapy for pain reduction instead of prescribing opioids [48, 51].

Furthermore, the need to consider consumers’/patients’ expectations of certain treatments was also observed in our results. *Engage consumers* (including ‘communication tools’, ‘distribute educational materials’) was used in at least 14 included SRs, and strategy-specific results were reported in eight SRs, providing effectiveness in four SRs. The assessment of the effectiveness of *engage consumers* as a discrete strategy is often complicated due to its integration within multifaceted de-implementation initiatives. Drawing insights from antibiotic utilisation reduction, it becomes evident that implementing delayed prescribing practices leads to a reduction in antibiotic prescriptions. This successful approach holds potential applicability in other healthcare domains, such as imaging procedures for low back pain, where patient requests often drive the demand for such procedures [77].

It is important to acknowledge that, beyond the selection of de-implementation strategies, various methodological factors within a study (e.g. study design), the design of the de-implementation initiative (i.e. length and intensity), and the absolute occurrence of a low-value practice, as well as the context, may significantly impact its effectiveness [74, 78]. This highlights the need for more concise reporting of these details in SRs.

However, SRs seldomly extracted data on the planning aspects of de-implementation initiatives (e.g. theory application, development process, tailoring methodologies), which impairs their results’ applicability [79]. While certain SRs examined aspects of de-implementation initiatives, their inclusion in our overview of systematic reviews was precluded due to inadequate emphasis on the effectiveness assessment [80, 81]. The limited attention given to effectiveness assessment in these SRs may stem from constraints such as space limitations inherent in journal publications, limited research grants or another aim. However, these SRs revealed that primary studies also often lacked that information. Therefore, to improve future SR conduct and reporting, the following suggestions should be considered along with suggestions for the reporting of primary studies [7]. The synthesis process should follow guidelines highlighting a complex intervention perspective (e.g. inclusion of a logic model to inform a synthesis, the limitations and strengths of certain study designs, and considering different results presentation forms) [82, 83]. SR reporting should include the development process of the de-implementation initiative and more details on the applied de-implementation strategies, such as dosage, temporality and fidelity and when such information was reported in the primary literature. This could foster the replication of studies and the investigation of the effectiveness as heterogeneity can be explored. Future SRs should also synthesise ‘appropriateness of care’. The use of LVC can only be considered an indirect measure of desired changes, as it does not adhere to guideline recommendations. To further knowledge on the effectiveness of different de-implementation strategies, a uniform reporting of these strategies as well as building on existing taxonomies would be needed, e.g. see Thompson Coon et al. [53]. A GRADE assessment of the relevant outcomes should also be added to enable an assessment of the evidence level.

Future research could focus on formulating synthesis methodologies that integrate efficacy requirements with a comprehensive depiction and analysis of de-implementation strategies. This could offer recommendations regarding the most appropriate discrete strategies or combinations thereof for addressing specific LVC practices, considering pertinent barriers to and facilitators for de- de-implementation [84]. Additionally, to facilitate the comparison of SR outcomes using various taxonomies for categorising de-implementation strategies, forthcoming methodological inquiries could explore the synergies between the ERIC and EPOC taxonomies [85].

### Strengths and limitations

A strength of our overview of systematic reviews was that we considered the effectiveness of de-implementation studies across different healthcare practices, therefore highlighting successful strategies potentially applicable to other healthcare practices. However, some limitations should be noted. First, though we used reference list checking and a preliminary search in different databases to inform and improve the main search strategy, we may have missed some SRs in our search due to different indexing and terms used in diverse health fields. Second, if SR results were presented narratively, we had to rely on vote counting based on the results’ statistical significance due to missing information in the included SRs. Not considering trends in the effect estimates or effect estimates in general may have influenced the results and overestimated the differences between different de-implementation strategies [74]. Third, when categorising the effect, we did not differentiate between no change or a negative effect (i.e. increase in the LVC practice investigated). Although the effect was sometimes reported on the individual primary study level [55, 71], it did not occur on the SR level. Fourth, due to limited resources and a lack of software availability we were unable to assess the overlap of included primary studies. Although we cannot exclude the possibility of some overlap, it may be minimal, as most SRs addressed different research questions and subgroups. Additionally, we aimed to minimise that effect by excluding SRs that included primary studies entailed in other SRs. Fifth, we were unable to code the de-implementation strategies at the ERIC strategies due to missing detailed descriptions in the studies. Therefore, we coded at the ERIC clusters. However, the cluster-level analyses diminished the differences of certain strategies as ‘audit and provide feedback’ and ‘develop and organise quality monitoring systems’ (e.g. peer feedback, accountability tool) are comprised in one cluster (i.e. *use evaluative and iterative strategies*). To highlight the relevant detailed information, we sometimes also added the specific strategies when describing the results.

### Limitations of the evidence base

The SRs included primary studies with different study designs; some focused solely on RCTs (especially within the antibiotics reduction field), but most included less methodologically sound study designs, such as uncontrolled before–after studies. Kobewka et al. [55] found that the median relative reductions were slightly smaller in studies investigating the reduction of laboratory test utilisation, if a concurrent control group was used (median 16.5, IQR: 5.8 to 27.0) in comparison to no concurrent control group (median 24.9, IQR: 14.0 to 47.5).

## Conclusions

The 46 SRs included in this overview of systematic reviews provided evidence for the effectiveness of de-implementation initiatives in medication utilisation. Inconsistent results were found for the reduction of laboratory tests and diagnostic imaging. *Change infrastructure and workflow* and *develop stakeholder interrelationships* were identified as the most promising de-implementation strategy categories. Suggestions for improving the SR conduct include following guidelines for the synthesis of complex interventions, focussing on the appropriateness of care outcomes, reporting on the development process and consistently reporting on the de-implementation strategies applied.

### Supplementary Information

Additional file 1. Including several tables and figures

Additional file 2. PRIOR Statement

Additional file 3. Detailed results of the included SRs

## Data Availability

The datasets used and/or analysed during the current study are available from the corresponding author on reasonable request. Most data are available as additional materials.

## Abbreviations

AMSTAR: A MeaSurement Tool to Assess systematic Reviews
CDSS: clinical decision support system
CI: confidence interval
e.g.: exempli gratia
CVCs: central venous catheters
EPOC: Effective Practice and Organisation of Care
ERIC: Expert Recommendations for Implementing Change
GRADE: Grading of Recommendations, Assessment, Development, and Evaluations
i.e.: id est
IQR: interquartile range
ITS: interrupted time series
LVC: low-value care
MA: meta-analysis
OR: odds ratio
PRESS: Peer Review of Electronic Search Strategies
PRIOR: Preferred Reporting Items for Overviews of Reviews
PRISMA: Preferred Reporting Items for Systematic Reviews and Meta-Analyses
RCT: randomised controlled trial
RD: risk difference
RoB: risk of bias
ROBIS: Risk of Bias In Systematic Reviews
RR: relative risk
SR: systematic review
SE: standard effect
