# Supplementary material for: Effectiveness of de-implementation of low-value healthcare practices: an overview of systematic reviews
Source: Implement Sci. 2024 Aug 5;19:56. doi: 10.1186/s13012-024-01384-6 (PMC11299416; doi:10.1186/s13012-024-01384-6)

Additional File 1

Effectiveness of de-implementation of low-value health care practices: an overview of systematic reviews

**Content**

[eTable 1: Eligibility criteria for the overview of reviews of de-implementation of LVC 2](#_Toc158967943)

[eTable 2: Search strategies overview 3](#_Toc158967944)

[List of excluded studies at full-text levels 11](#_Toc158967945)

[eTable 3: Assessment of the confidence in the results (according to AMSTAR 2) 26](#_Toc158967946)

[eTable 4: Mapping mentioned de-implementation strategies onto ERIC 29](#_Toc158967947)

[eTable 5: Description of SRs rated as critically low confidence 35](#_Toc158967948)

[eTable 6: Assessment of the confidence in the results (according to AMSTAR 2), only critically low SRs 45](#_Toc158967949)

[eTable 7: Description SRs not extracted 49](#_Toc158967950)

[eTable 8: Characteristics of included SRs 53](#_Toc158967951)

[eTable 9: Setting and healthcare practice targeted 87](#_Toc158967952)

[eTable 10: De-implementation strategies reported 97](#_Toc158967953)

[eTable 11: Details of de-implementation strategies reported 120](#_Toc158967954)

[eTable 12: Study population in included SR 127](#_Toc158967955)

[eFigure 1: Frequency of higher-level ERIC de-implementation categories within the medical intervention categories 134](#_Toc158967956)

# eTable 1: Eligibility criteria for the overview of reviews of de-implementation of LVC

|  | **Inclusion criteria** | **Exclusion criteria** |
| --- | --- | --- |
| **Population** | - paediatric or adult patients | - No exclusion criteria defined |
| **Intervention** | - De-implementation interventions across all physiological, mental and social health specialties of LVC practices to reduce LVP practices | - Studies assessing the need for de-implementation - Barriers and facilitators - Prevalence of LVC |
| **Control** | - Placebo - Treatment as usual - Other active interventions - Comparisons over time (before-after studies, interrupted time series) | - Studies without a comparison |
| **Outcomes** | - Appropriateness of intervention use (overuse, underuse, misuse, other, intention to reduce the use of LVC) - Utilization/ordering of LVC | - Prevalence of LVC - No outcomes reported - Cost-effectiveness outcomes |
| **Study designs** | - systematic *effectiveness* reviews according to Cochrane Handbook | - all other study designs |

Abbreviations: LVC: low-value care

# eTable 2: Search strategies overview

| Database and host | Date searched | Hits |
| --- | --- | --- |
| Ovid MEDLINE(R) ALL 1946 to April 14, 2023 | 17.04.2023 | 1285 |
| Scopus (Elsevier) | 17.04.2023 | 292 |
| Epistemonikos.org | 17.04.2023 | 1854 |

| **Total (before deduplication)** | **3431** |
| --- | --- |
| **Total (after deduplication)** | **2603** |

## Ovid MEDLINE(R) ALL 1946 to April 14, 2023

17.04.2023

| Concept | # | Searches | Results |
| --- | --- | --- | --- |
| A. de-implementation | 1 | Inappropriate Prescribing/pc [Prevention & Control] | 1581 |
|  | 2 | exp Medical Overuse/pc [Prevention & Control] | 693 |
|  | 3 | low-value care/ | 57 |
|  | 4 | deprescriptions/ | 969 |
|  | 5 | Potentially Inappropriate Medication List/ | 966 |
|  | 6 | (de-implement* or deimplement* or de-prescri* or deprescri* or dis-invest* or disinvest* or deadopt* or de-adopt* or disadopt* or dis-adopt*).ti,bt,ab,kf. | 3244 |
|  | 7 | Choosing Wisely.ti,bt,ab,kf. | 1139 |
|  | 8 | Health Technology Reassessment.ti,bt,ab,kf. | 31 |
|  | 9 | (low-value or inappropriate or unnecessary or overmedication or over-medication or overtreat* or over-treat* or overuse or underuse or redundant).ti,bt,kf. | 21290 |
|  | 10 | ((prescri* or ordering or usage or utili?ation) adj6 (reduc* or decreas*)).ti,bt,kf. | 2483 |
|  | 11 | ((incentiv* or improv* or prescri* or management) adj6 appropriate).ti,bt,kf. | 911 |
|  | 12 | (chang* and (care or healthcare or physician? or nurse? or clinical or clinician? or therapist? or provider?) and (pattern? or pathway? or practice?)).ti,bt,kf. | 3238 |
|  | 13 | (low-value or inappropriate or unnecessary or redundan* or obsole*).ab. /freq=3 | 7902 |
|  | 14 | or/1-13 | 38044 |
| B. reducing unnecessary care | 15 | ((improv* or reduc* or decreas* or stop* or discontinu* or incentiv* or reassess* or re-assess*) and (prescribing or prescription or imaging or screening or testing or pattern? or pathway? or treat* or therap* or practice?)).ti,bt,kf. | 128026 |
|  | 16 | (low-value or inappropriate or unnecessary or redundan* or obsole*).ti,bt,ab,kf. | 192314 |
|  | 17 | 15 and 16 | 2239 |
| C. healthcare practice | 18 | *Drug Prescriptions/ | 17440 |
|  | 19 | exp *Mass Screening/ | 75599 |
|  | 20 | *Diagnostic Tests, Routine/ | 8502 |
|  | 21 | exp *Surgical Procedures, Operative/ | 2257249 |
|  | 22 | (prescribing or prescription or ordering or usage or utili?ation or imaging or screening or testing or surg*).ti,bt,kf. | 1784705 |
|  | 23 | or/18-22 | 3628895 |
| D. decision-making | 24 | Decision Making/ | 104228 |
|  | 25 | implementation science/ | 1250 |
|  | 26 | Choice Behavior/ | 34854 |
|  | 27 | practice patterns, pharmacists'/ or practice patterns, dentists'/ or practice patterns, nurses'/ or practice patterns, physicians'/ | 71971 |
|  | 28 | exp Resource Allocation/ or exp health resources/ | 46876 |
|  | 29 | (pathway? or practice? or decision*).ti,bt,kf. | 698813 |
|  | 30 | (pathway? or practice? or decision*).ab. /freq=2 | 880005 |
|  | 31 | (resource? adj2 (allocation or reallocation)).ti,ab,kf,bt. | 15968 |
|  | 32 | or/24-31 | 1464823 |
| E. quality improvement | 33 | Quality Improvement/ | 32226 |
|  | 34 | Inappropriate Prescribing/ | 4537 |
|  | 35 | exp Medical Overuse/ | 14892 |
|  | 36 | (low-value or inappropriate or unnecessary or redundan*).ti,bt,ab,kf. | 186992 |
|  | 37 | (improv* or reduc* or decreas* or incentiv* or stop* or discontinu*).ti,bt,kf. | 913794 |
|  | 38 | or/33-37 | 1117909 |
| C+D+E | 39 | 23 and 32 and 38 | 14233 |
| A or B or (C+D+E) | 40 | 14 or 17 or 39 | 50968 |
| SR-Filter | 41 | (((systematic* and review?) or Systematic overview* or ((Cochrane or systemic or scoping or mapping or Umbrella) adj review*) or ((Cochrane or systemic or scoping or mapping or Umbrella) adj literature review*) or "review of reviews" or "overview of reviews" or meta-review or (integrat* adj (review or overview)) or meta-synthes?s or metasynthes?s or "quantitative review" or "quantitative synthesis" or "research synthesis" or meta-ethnography or "Systematic literature search" or "Systematic literature research" or meta-analys?s or metaanalys?s or "meta-analytic review" or "meta-analytical review").ti,kf,bt. or meta-analysis.pt. or Network Meta-Analysis/ or ((search* or medline or pubmed or embase or Cochrane or scopus or "web of science" or "sources of information" or "data sources" or "following databases") and ("study selection" or "selection criteria" or "eligibility criteria" or "inclusion criteria" or "exclusion criteria")).tw. or "systematic review".pt.) not ((letter or editorial or comment or "case reports" or "historical article").pt. or report.ti. or protocol.ti. or protocols.ti. or withdrawn.ti. or "retraction of publication".pt. or exp "retraction of publication as topic"/ or "retracted publication".pt. or reply.ti. or "published erratum".pt.) | 397081 |
| SR-Results | 42 | 40 and 41 | 1385 |
| limited to language | 43 | (english or german).lg. | 31665834 |
|  | 44 | 42 and 43 | 1368 |
| limited to 2010-present | 45 | limit 44 to yr="2010 -Current" | 1285 |

## Scopus (Elsevier)

17.04.2023

| History Count | Search Terms | Results |
| --- | --- | --- |
| 1 | TITLE-ABS-KEY ( ( de-implement*  OR  deimplement*  OR  de-prescri*  OR  deprescri*  OR  dis-invest*  OR  disinvest*  OR  deadopt*  OR  de-adopt*  OR  disadopt*  OR  dis-adopt* ) ) | 5,789 document results |
| 3 | TITLE-ABS-KEY ( {Choosing Wisely}  OR  {Health Technology Reassessment} ) | 1,230 document results |
| 4 | TITLE ( low-value  OR  inappropriate  OR  unnecessary  OR  overmedication  OR  over-medication  OR  overtreat*  OR  over-treat*  OR  overuse  OR  underuse  OR  redundant ) | 34,019 document results |
| 5 | TITLE ( ( prescri*  OR  ordering  OR  usage  OR  utili*ation )  W/6  ( reduc*  OR  decreas* ) ) | 4,233 document results |
| 6 | TITLE ( ( incentiv*  OR  improv*  OR  prescri*  OR  management )  W/6  appropriate ) | 1,515 document results |
| 7 | TITLE ( chang*  AND  ( care  OR  healthcare  OR  physician*  OR  nurse*  OR  clinical  OR  clinician*  OR  therapist*  OR  provider* )  AND  ( pattern*  OR  pathway*  OR  practice* ) ) | 2,791 document results |
| 8 | TITLE ( ( improv*  OR  reduc*  OR  decreas*  OR  stop*  OR  discontinu*  OR  incentiv*  OR  reassess*  OR  re-assess* )  AND  ( prescribing  OR  prescription  OR  imaging  OR  screening  OR  testing  OR  pattern*  OR  pathway*  OR  treat*  OR  therap*  OR  practice* ) )  AND  TITLE-ABS-KEY ( low-value  OR  inappropriate  OR  unnecessary  OR  redundan*  OR  obsole* ) | 2,508 document results |
| 9 | TITLE ( ( prescribing  OR  prescription  OR  ordering  OR  usage  OR  utili*ation  OR  imaging  OR  screening  OR  testing  OR  surg* )  AND  ( pathway*  OR  practice*  OR  decision* ) )  AND  TITLE-ABS-KEY ( low-value  OR  inappropriate  OR  unnecessary  OR  redundan*  OR  improv*  OR  reduc*  OR  decreas*  OR  incentiv*  OR  stop*  OR  discontinu* ) | 12,662 document results |
| 10 | TITLE ( prescribing  OR  prescription  OR  ordering  OR  usage  OR  utili*ation  OR  imaging  OR  screening  OR  testing  OR  surg* )  AND  ( pathway*  OR  practice*  OR  decision* )  AND  TITLE-ABS-KEY ( resource*  W/2  ( allocation  OR  reallocation ) )  AND  TITLE-ABS-KEY ( low-value  OR  inappropriate  OR  unnecessary  OR  redundan*  OR  improv*  OR  reduc*  OR  decreas*  OR  incentiv*  OR  stop*  OR  discontinu* ) | 932 document results |
| 11 | ( TITLE ( prescribing  OR  prescription  OR  ordering  OR  usage  OR  utili*ation  OR  imaging  OR  screening  OR  testing  OR  surg* )  AND  ( pathway*  OR  practice*  OR  decision* )  AND  TITLE-ABS-KEY ( resource*  W/2  ( allocation  OR  reallocation ) )  AND  TITLE-ABS-KEY ( low-value  OR  inappropriate  OR  unnecessary  OR  redundan*  OR  improv*  OR  reduc*  OR  decreas*  OR  incentiv*  OR  stop*  OR  discontinu* ) )  OR  ( TITLE ( ( prescribing  OR  prescription  OR  ordering  OR  usage  OR  utili*ation  OR  imaging  OR  screening  OR  testing  OR  surg* )  AND  ( pathway*  OR  practice*  OR  decision* ) )  AND  TITLE-ABS-KEY ( low-value  OR  inappropriate  OR  unnecessary  OR  redundan*  OR  improv*  OR  reduc*  OR  decreas*  OR  incentiv*  OR  stop*  OR  discontinu* ) )  OR  ( TITLE ( ( improv*  OR  reduc*  OR  decreas*  OR  stop*  OR  discontinu*  OR  incentiv*  OR  reassess*  OR  re-assess* )  AND  ( prescribing  OR  prescription  OR  imaging  OR  screening  OR  testing  OR  pattern*  OR  pathway*  OR  treat*  OR  therap*  OR  practice* ) )  AND  TITLE-ABS-KEY ( low-value  OR  inappropriate  OR  unnecessary  OR  redundan*  OR  obsole* ) )  OR  ( TITLE ( chang*  AND  ( care  OR  healthcare  OR  physician*  OR  nurse*  OR  clinical  OR  clinician*  OR  therapist*  OR  provider* )  AND  ( pattern*  OR  pathway*  OR  practice* ) ) )  OR  ( TITLE ( ( incentiv*  OR  improv*  OR  prescri*  OR  management )  W/6  appropriate ) )  OR  ( TITLE ( ( prescri*  OR  ordering  OR  usage  OR  utili*ation )  W/6  ( reduc*  OR  decreas* ) ) )  OR  ( TITLE ( low-value  OR  inappropriate  OR  unnecessary  OR  overmedication  OR  over-medication  OR  overtreat*  OR  over-treat*  OR  overuse  OR  underuse  OR  redundant ) )  OR  ( TITLE-ABS-KEY ( {Choosing Wisely}  OR  {Health Technology Reassessment} ) )  OR  ( TITLE-ABS-KEY ( ( de-implement*  OR  deimplement*  OR  de-prescri*  OR  deprescri*  OR  dis-invest*  OR  disinvest*  OR  deadopt*  OR  de-adopt*  OR  disadopt*  OR  dis-adopt* ) ) ) | 63,498 document results |
| 12 | INDEX ( medline ) | 30,024,022 document results |
| 13 | ( ( TITLE ( prescribing  OR  prescription  OR  ordering  OR  usage  OR  utili*ation  OR  imaging  OR  screening  OR  testing  OR  surg* )  AND  ( pathway*  OR  practice*  OR  decision* )  AND  TITLE-ABS-KEY ( resource*  W/2  ( allocation  OR  reallocation ) )  AND  TITLE-ABS-KEY ( low-value  OR  inappropriate  OR  unnecessary  OR  redundan*  OR  improv*  OR  reduc*  OR  decreas*  OR  incentiv*  OR  stop*  OR  discontinu* ) )  OR  ( TITLE ( ( prescribing  OR  prescription  OR  ordering  OR  usage  OR  utili*ation  OR  imaging  OR  screening  OR  testing  OR  surg* )  AND  ( pathway*  OR  practice*  OR  decision* ) )  AND  TITLE-ABS-KEY ( low-value  OR  inappropriate  OR  unnecessary  OR  redundan*  OR  improv*  OR  reduc*  OR  decreas*  OR  incentiv*  OR  stop*  OR  discontinu* ) )  OR  ( TITLE ( ( improv*  OR  reduc*  OR  decreas*  OR  stop*  OR  discontinu*  OR  incentiv*  OR  reassess*  OR  re-assess* )  AND  ( prescribing  OR  prescription  OR  imaging  OR  screening  OR  testing  OR  pattern*  OR  pathway*  OR  treat*  OR  therap*  OR  practice* ) )  AND  TITLE-ABS-KEY ( low-value  OR  inappropriate  OR  unnecessary  OR  redundan*  OR  obsole* ) )  OR  ( TITLE ( chang*  AND  ( care  OR  healthcare  OR  physician*  OR  nurse*  OR  clinical  OR  clinician*  OR  therapist*  OR  provider* )  AND  ( pattern*  OR  pathway*  OR  practice* ) ) )  OR  ( TITLE ( ( incentiv*  OR  improv*  OR  prescri*  OR  management )  W/6  appropriate ) )  OR  ( TITLE ( ( prescri*  OR  ordering  OR  usage  OR  utili*ation )  W/6  ( reduc*  OR  decreas* ) ) )  OR  ( TITLE ( low-value  OR  inappropriate  OR  unnecessary  OR  overmedication  OR  over-medication  OR  overtreat*  OR  over-treat*  OR  overuse  OR  underuse  OR  redundant ) )  OR  ( TITLE-ABS-KEY ( {Choosing Wisely}  OR  {Health Technology Reassessment} ) )  OR  ( TITLE-ABS-KEY ( ( de-implement*  OR  deimplement*  OR  de-prescri*  OR  deprescri*  OR  dis-invest*  OR  disinvest*  OR  deadopt*  OR  de-adopt*  OR  disadopt*  OR  dis-adopt* ) ) ) )  AND NOT  ( INDEX ( medline ) ) | 29,524 document results |
| 14 | SUBJAREA ( phar  OR  medi  OR  nurs  OR  vete  OR  dent  OR  heal  OR  mult  OR  psyc  OR  soci ) | 41,378,335 document results |
| 15 | ( ( ( TITLE ( prescribing  OR  prescription  OR  ordering  OR  usage  OR  utili*ation  OR  imaging  OR  screening  OR  testing  OR  surg* )  AND  ( pathway*  OR  practice*  OR  decision* )  AND  TITLE-ABS-KEY ( resource*  W/2  ( allocation  OR  reallocation ) )  AND  TITLE-ABS-KEY ( low-value  OR  inappropriate  OR  unnecessary  OR  redundan*  OR  improv*  OR  reduc*  OR  decreas*  OR  incentiv*  OR  stop*  OR  discontinu* ) )  OR  ( TITLE ( ( prescribing  OR  prescription  OR  ordering  OR  usage  OR  utili*ation  OR  imaging  OR  screening  OR  testing  OR  surg* )  AND  ( pathway*  OR  practice*  OR  decision* ) )  AND  TITLE-ABS-KEY ( low-value  OR  inappropriate  OR  unnecessary  OR  redundan*  OR  improv*  OR  reduc*  OR  decreas*  OR  incentiv*  OR  stop*  OR  discontinu* ) )  OR  ( TITLE ( ( improv*  OR  reduc*  OR  decreas*  OR  stop*  OR  discontinu*  OR  incentiv*  OR  reassess*  OR  re-assess* )  AND  ( prescribing  OR  prescription  OR  imaging  OR  screening  OR  testing  OR  pattern*  OR  pathway*  OR  treat*  OR  therap*  OR  practice* ) )  AND  TITLE-ABS-KEY ( low-value  OR  inappropriate  OR  unnecessary  OR  redundan*  OR  obsole* ) )  OR  ( TITLE ( chang*  AND  ( care  OR  healthcare  OR  physician*  OR  nurse*  OR  clinical  OR  clinician*  OR  therapist*  OR  provider* )  AND  ( pattern*  OR  pathway*  OR  practice* ) ) )  OR  ( TITLE ( ( incentiv*  OR  improv*  OR  prescri*  OR  management )  W/6  appropriate ) )  OR  ( TITLE ( ( prescri*  OR  ordering  OR  usage  OR  utili*ation )  W/6  ( reduc*  OR  decreas* ) ) )  OR  ( TITLE ( low-value  OR  inappropriate  OR  unnecessary  OR  overmedication  OR  over-medication  OR  overtreat*  OR  over-treat*  OR  overuse  OR  underuse  OR  redundant ) )  OR  ( TITLE-ABS-KEY ( {Choosing Wisely}  OR  {Health Technology Reassessment} ) )  OR  ( TITLE-ABS-KEY ( ( de-implement*  OR  deimplement*  OR  de-prescri*  OR  deprescri*  OR  dis-invest*  OR  disinvest*  OR  deadopt*  OR  de-adopt*  OR  disadopt*  OR  dis-adopt* ) ) ) )  AND NOT  ( INDEX ( medline ) ) )  AND  ( SUBJAREA ( phar  OR  medi  OR  nurs  OR  vete  OR  dent  OR  heal  OR  mult  OR  psyc  OR  soci ) ) | 13,087 document results |
| 16 | TITLE ( ( systematic*  AND  review* )  OR  "Systematic overview*"  OR  ( ( cochrane  OR  systemic  OR  scoping  OR  mapping  OR  umbrella )  W/1  review* )  OR  ( ( cochrane  OR  systemic  OR  scoping  OR  mapping  OR  umbrella )  W/1  "literature review*" )  OR  "review of reviews"  OR  "overview of reviews"  OR  meta-review  OR  ( integrat*  W/1  ( review  OR  overview ) )  OR  meta-synthes*s  OR  metasynthes*s  OR  "quantitative review"  OR  "quantitative synthesis"  OR  "research synthesis"  OR  meta-ethnography  OR  "Systematic literature search"  OR  "Systematic literature research"  OR  meta-analys*s  OR  metaanalys*s  OR  "meta-analytic review"  OR  "meta-analytical review" )  OR  KEY ( ( systematic*  AND  review* )  OR  "Systematic overview*"  OR  ( ( cochrane  OR  systemic  OR  scoping  OR  mapping  OR  umbrella )  W/1  review* )  OR  ( ( cochrane  OR  systemic  OR  scoping  OR  mapping  OR  umbrella )  W/1  "literature review*" )  OR  "review of reviews"  OR  "overview of reviews"  OR  meta-review  OR  ( integrat*  W/1  ( review  OR  overview ) )  OR  meta-synthes*s  OR  metasynthes*s  OR  "quantitative review"  OR  "quantitative synthesis"  OR  "research synthesis"  OR  meta-ethnography  OR  "Systematic literature search"  OR  "Systematic literature research"  OR  meta-analys*s  OR  metaanalys*s  OR  "meta-analytic review"  OR  "meta-analytical review" )  OR  TITLE-ABS-KEY ( ( search*  OR  medline  OR  pubmed  OR  embase  OR  cochrane  OR  scopus  OR  "web of science"  OR  "sources of information"  OR  "data sources"  OR  "following databases" )  AND  ( "study selection"  OR  "selection criteria"  OR  "eligibility criteria"  OR  "inclusion criteria"  OR  "exclusion criteria" ) ) | 641,758 document results |
| 17 | ( ( ( ( TITLE ( prescribing  OR  prescription  OR  ordering  OR  usage  OR  utili*ation  OR  imaging  OR  screening  OR  testing  OR  surg* )  AND  ( pathway*  OR  practice*  OR  decision* )  AND  TITLE-ABS-KEY ( resource*  W/2  ( allocation  OR  reallocation ) )  AND  TITLE-ABS-KEY ( low-value  OR  inappropriate  OR  unnecessary  OR  redundan*  OR  improv*  OR  reduc*  OR  decreas*  OR  incentiv*  OR  stop*  OR  discontinu* ) )  OR  ( TITLE ( ( prescribing  OR  prescription  OR  ordering  OR  usage  OR  utili*ation  OR  imaging  OR  screening  OR  testing  OR  surg* )  AND  ( pathway*  OR  practice*  OR  decision* ) )  AND  TITLE-ABS-KEY ( low-value  OR  inappropriate  OR  unnecessary  OR  redundan*  OR  improv*  OR  reduc*  OR  decreas*  OR  incentiv*  OR  stop*  OR  discontinu* ) )  OR  ( TITLE ( ( improv*  OR  reduc*  OR  decreas*  OR  stop*  OR  discontinu*  OR  incentiv*  OR  reassess*  OR  re-assess* )  AND  ( prescribing  OR  prescription  OR  imaging  OR  screening  OR  testing  OR  pattern*  OR  pathway*  OR  treat*  OR  therap*  OR  practice* ) )  AND  TITLE-ABS-KEY ( low-value  OR  inappropriate  OR  unnecessary  OR  redundan*  OR  obsole* ) )  OR  ( TITLE ( chang*  AND  ( care  OR  healthcare  OR  physician*  OR  nurse*  OR  clinical  OR  clinician*  OR  therapist*  OR  provider* )  AND  ( pattern*  OR  pathway*  OR  practice* ) ) )  OR  ( TITLE ( ( incentiv*  OR  improv*  OR  prescri*  OR  management )  W/6  appropriate ) )  OR  ( TITLE ( ( prescri*  OR  ordering  OR  usage  OR  utili*ation )  W/6  ( reduc*  OR  decreas* ) ) )  OR  ( TITLE ( low-value  OR  inappropriate  OR  unnecessary  OR  overmedication  OR  over-medication  OR  overtreat*  OR  over-treat*  OR  overuse  OR  underuse  OR  redundant ) )  OR  ( TITLE-ABS-KEY ( {Choosing Wisely}  OR  {Health Technology Reassessment} ) )  OR  ( TITLE-ABS-KEY ( ( de-implement*  OR  deimplement*  OR  de-prescri*  OR  deprescri*  OR  dis-invest*  OR  disinvest*  OR  deadopt*  OR  de-adopt*  OR  disadopt*  OR  dis-adopt* ) ) ) )  AND NOT  ( INDEX ( medline ) ) )  AND  ( SUBJAREA ( phar  OR  medi  OR  nurs  OR  vete  OR  dent  OR  heal  OR  mult  OR  psyc  OR  soci ) ) )  AND  ( TITLE ( ( systematic*  AND  review* )  OR  "Systematic overview*"  OR  ( ( cochrane  OR  systemic  OR  scoping  OR  mapping  OR  umbrella )  W/1  review* )  OR  ( ( cochrane  OR  systemic  OR  scoping  OR  mapping  OR  umbrella )  W/1  "literature review*" )  OR  "review of reviews"  OR  "overview of reviews"  OR  meta-review  OR  ( integrat*  W/1  ( review  OR  overview ) )  OR  meta-synthes*s  OR  metasynthes*s  OR  "quantitative review"  OR  "quantitative synthesis"  OR  "research synthesis"  OR  meta-ethnography  OR  "Systematic literature search"  OR  "Systematic literature research"  OR  meta-analys*s  OR  metaanalys*s  OR  "meta-analytic review"  OR  "meta-analytical review" )  OR  KEY ( ( systematic*  AND  review* )  OR  "Systematic overview*"  OR  ( ( cochrane  OR  systemic  OR  scoping  OR  mapping  OR  umbrella )  W/1  review* )  OR  ( ( cochrane  OR  systemic  OR  scoping  OR  mapping  OR  umbrella )  W/1  "literature review*" )  OR  "review of reviews"  OR  "overview of reviews"  OR  meta-review  OR  ( integrat*  W/1  ( review  OR  overview ) )  OR  meta-synthes*s  OR  metasynthes*s  OR  "quantitative review"  OR  "quantitative synthesis"  OR  "research synthesis"  OR  meta-ethnography  OR  "Systematic literature search"  OR  "Systematic literature research"  OR  meta-analys*s  OR  metaanalys*s  OR  "meta-analytic review"  OR  "meta-analytical review" )  OR  TITLE-ABS-KEY ( ( search*  OR  medline  OR  pubmed  OR  embase  OR  cochrane  OR  scopus  OR  "web of science"  OR  "sources of information"  OR  "data sources"  OR  "following databases" )  AND  ( "study selection"  OR  "selection criteria"  OR  "eligibility criteria"  OR  "inclusion criteria"  OR  "exclusion criteria" ) ) )  AND PUBYEAR > 2009 | 292 document results |

## Epistemonikos.org

17.04.2023

| Search 1 | Results |
| --- | --- |
| "de-implementation" OR deimplement* OR "de-prescription" OR "de-prescribing" OR deprescri* OR "dis-invest" OR "dis-investment" OR disinvest* OR deadopt* OR "de-adoption" OR disadopt* OR "dis-adoption" OR "Choosing Wisely" OR "Health Technology Reassessment" | 4640 |
| Filter: Systematic Review | 1138 |
| Filter: Broad Synthesis | 49 |
|  |  |
| Search 2 |  |
| title:("low-value" OR inappropriate OR unnecessary OR overmedication OR "over-medication" OR overtreat* OR "over-treatment" OR overuse OR underuse OR redundant OR ((prescri* OR ordering OR usage OR utilization OR utilisation) AND (reduc* OR decreas*)) OR ((incentiv* OR improv* OR prescri* OR management) AND appropriate) OR (chang* AND (care OR healthcare OR physician* OR nurse* OR clinical OR clinician* OR therapist* OR provider*) AND (pattern* OR pathway* OR practice*))) | 2844 |
| Filter: Systematic Review | 535 |
| Filter: Broad Synthesis | 36 |
|  |  |
| Search 3 |  |
| title:((improv* OR reduc* OR decreas* OR stop* OR discontinu* OR incentiv* OR reassess* OR "re-assess" OR "re-assessment") AND (prescribing OR prescription OR imaging OR screening OR testing OR pattern* OR pathway* OR treat* OR therap* OR practice*)) AND (title:("low-value" OR inappropriate OR unnecessary OR redundan* OR obsole*) OR abstract:("low-value" OR inappropriate OR unnecessary OR redundan* OR obsole*)) | 397 |
| Filter: Systematic Review | 83 |
| Filter: Broad Synthesis | 4 |
|  |  |
| Search 4 |  |
| title:(prescribing OR prescription OR ordering OR usage OR utilization OR utilisation OR imaging OR screening OR testing OR surg*) AND title:(pathway* OR practice* OR decision*) AND (title:("low-value" OR inappropriate OR unnecessary OR redundan* OR improv* OR reduc* OR decreas* OR incentiv* OR stop* OR discontinu*) OR abstract:("low-value" OR inappropriate OR unnecessary OR redundan* OR improv* OR reduc* OR decreas* OR incentiv* OR stop* OR discontinu*)) | 2301 |
| Filter: Systematic Review | 399 |
| Filter: Broad Synthesis | 36 |
|  |  |
| Total (including duplicates) | 2280 |
| Limited to 2010-present | 1854 |

# List of excluded studies at full-text levels

## Ineligible research question (15)

1. Agarwal R, Mazurenko O, Menachemi N. High-Deductible Health Plans Reduce Health Care Cost And Utilization, Including Use Of Needed Preventive Services. Health affairs (Project Hope). 2017;36(10):1762-1768. doi:10.1377/hlthaff.2017.0610

2. Baxi SS, Kale M, Keyhani S, et al. Overuse of Health Care Services in the Management of Cancer: A Systematic Review. Review; Med Care. 07 2017;55(7):723-733. doi:10.1097/MLR.0000000000000734

3. Flokas ME, Andreatos N, Alevizakos M, Kalbasi A, Onur P, Mylonakis E. Inappropriate Management of Asymptomatic Patients With Positive Urine Cultures: A Systematic Review and Meta-analysis. Open forum infect. 2017;4(4):ofx207. doi:10.1093/ofid/ofx207

4. Hanlon JT, Schmader KE. The medication appropriateness index at 20: where it started, where it has been, and where it may be going. Drugs Aging. 2013;30(11):893-900. doi:10.1007/s40266-013-0118-4

5. Herledan C, Cerfon MA, Baudouin A, et al. Impact of pharmaceutical care interventions on multidisciplinary care of older patients with cancer: A systematic review. J Geriatr Oncol. Feb 20 2023:101450. doi:10.1016/j.jgo.2023.101450

6. Hoyle DJ, Bindoff IK, Clinnick LM, Peterson GM, Westbury JL. Clinical and Economic Outcomes of Interventions to Reduce Antipsychotic and Benzodiazepine Use Within Nursing Homes: A Systematic Review. Drugs Aging. 2018;35(2):123-134. doi:10.1007/s40266-018-0518-6

7. King S, Tancredi D, Lenoir-Wijnkoop I, et al. Does probiotic consumption reduce antibiotic utilization for common acute infections? A systematic review and meta-analysis. Eur J Public Health. 06 01 2019;29(3):494-499. doi:10.1093/eurpub/cky185

8. Kopterides P, Siempos, II, Tsangaris I, Tsantes A, Armaganidis A. Procalcitonin-guided algorithms of antibiotic therapy in the intensive care unit: A systematic review and meta-analysis of randomized controlled trials. Crit Care Med. 2010;38(11):2229-41. doi:10.1097/CCM.0b013e3181f17bf9

9. Laberge M, Sirois C, Lunghi C, et al. Economic Evaluations of Interventions to Optimize Medication Use in Older Adults with Polypharmacy and Multimorbidity: A Systematic Review. Clin Interv Aging. 2021;16:767-779. doi:10.2147/CIA.S304074

10. Pepper DJ, Sun J, Rhee C, et al. Procalcitonin-Guided Antibiotic Discontinuation and Mortality in Critically Ill Adults: A Systematic Review and Meta-analysis. Chest. 06 2019;155(6):1109-1118. doi:10.1016/j.chest.2018.12.029

11. Reeve E, Gnjidic D, Long J, Hilmer S. A systematic review of the emerging definition of 'deprescribing' with network analysis: implications for future research and clinical practice. Br J Clin Pharmacol. Dec 2015;80(6):1254-68.

12. Schiavo G, Forgerini M, Lucchetta RC, Mastroianni PC. A comprehensive look at explicit screening tools for potentially inappropriate medication: A systematic scoping review. Australas J Ageing. Sep 2022;41(3):357-382. doi:10.1111/ajag.13046

13. Schuetz P, Muller B, Christ-Crain M, et al. Procalcitonin to initiate or discontinue antibiotics in acute respiratory tract infections. Evidence-based child health: a Cochrane review journal. 2013;8(4):1297-371. doi:10.1002/ebch.1927

14. Wilsdon TD, Hendrix I, Thynne TR, Mangoni AA. Effectiveness of Interventions to Deprescribe Inappropriate Proton Pump Inhibitors in Older Adults. Drugs Aging. 04 2017;34(4):265-287. doi:10.1007/s40266-017-0442-1

15. Wolfman W, Thurston J, Yeung G, Glanc P. Guideline No. 404: Initial Investigation and Management of Benign Ovarian Masses. Practice Guideline. J Obstet Gynaecol Can. 08 2020;42(8):1040-1050.e1. doi:10.1016/j.jogc.2020.01.014

## Ineligible language (3)

1. Forsetlund L, Eike MC, Gjerberg E, Vist G. Effect of Interventions to Reduce Potentially Inappropriate Use of Medicines in Nursing Homes: A Systematic Review of Randomised Controlled Trials. NIPH Systematic Reviews: Executive Summaries. 2010.

2. Kuo YC, Tan ECH. Low-value care: International development experiences and the inspiration for Taiwan. Article. Taiwain J Public Health. 2021;40(2):151-165. doi:10.6288/TJPH.202104_40(2).109142

3. Zavala-Gonzalez MA, Cabrera-Pivaral CE, Orozco-Valerio MJ. Effectiveness of interventions for drug prescribing improvement in primary health care. Pharmacoepidemiology and Drug Safety. 2015;24(SUPPL. 1):63. doi:10.1002/pds.3838

## Ineligible document types (5)

1. Baxi SS, Kale M, Roman BR, et al. Systematic review of overuse in oncology. J Clin Oncol. 2016;

2. Lang I, Rogers M, Abbott R, et al. How can we reduce inappropriate prescribing of antipsychotic medications for people with dementia in residential care settings? a systematic review of implementation strategies. Alzheimer's and Dementia. 2014:P737.

3. Loganathan M, Singh S, Bottle A, et al. P26 Interventions to improve prescribing quality in care homes: a systematic review. J Epidemiol Community Health. 2010;64

4. Patterson SM, Bradley MC, Kerse N, Cardwell CR, Hughes CM. Interventions to improve the appropriate use of polypharmacy for older people: A cochrane systematic review. Pharmacoepidemiology and Drug Safety. 2013:685-686. doi:10.1002/pds.3428

5. Steinberg DI. 2017 - Review: Interventions improve hospital antibiotic prescribing and reduce hospital stay but do not affect mortality. ACP Journal Club. 2017;166(10):10-10.

## Ineligible study designs (16)

1. Cliff BQ, Avancena ALV, Hirth RA, Lee SD. The Impact of Choosing Wisely Interventions on Low-Value Medical Services: A Systematic Review. Milbank Q. 12 2021;99(4):1024-1058. doi:10.1111/1468-0009.12531

2. Clyne B, Bradley MC, Hughes C, Fahey T, Lapane KL. Electronic prescribing and other forms of technology to reduce inappropriate medication use and polypharmacy in older people: a review of current evidence. Clinics in geriatric medicine. 2012;28(2):301-22. doi:10.1016/j.cger.2012.01.009

3. Elmståhl S, Linder H. Polypharmacy and Inappropriate Drug Use among Older People-a Systematic Review. Healthy Aging & Clinical Care in the Elderly. 2013;(5):1-8. doi:10.4137/HACCE.S11173

4. Feazel LM, Malhotra A, Perencevich EN, Kaboli P, Diekema DJ, Schweizer ML. Effect of antibiotic stewardship programmes on Clostridium difficile incidence: a systematic review and meta-analysis. J Antimicrob Chemother. Jul 2014;69(7):1748-54. doi:10.1093/jac/dku046

5. Ho MJ, Venci J. Improving the success of mailed letter intervention programs to influence prescribing behaviors: a review. J Manage Care Pharm. Oct 2012;18(8):627-49.

6. Marcum ZA, Handler SM, Wright R, Hanlon JT. Interventions to improve suboptimal prescribing in nursing homes: A narrative review. Am J Geriatr Pharmacother. Jun 2010;8(3):183-200. doi:10.1016/j.amjopharm.2010.05.004

7. Morgan DJ, Dhruva SS, Coon ER, Wright SM, Korenstein D. 2017 Update on Medical Overuse: A Systematic Review. JAMA Intern Med. 01 01 2018;178(1):110-115. doi:10.1001/jamainternmed.2017.4361

8. Morgan DJ, Dhruva SS, Coon ER, Wright SM, Korenstein D. 2019 Update on Medical Overuse: A Review. JAMA Intern Med. 2019;179(11):1568-1574. doi:10.1001/jamainternmed.2019.3842

9. Nasr Z, Paravattil B, Wilby KJ. The impact of antimicrobial stewardship strategies on antibiotic appropriateness and prescribing behaviours in selected countries in the Middle East: a systematic review. East Mediterr Health J. Aug 20 2017;23(6):430-440.

10. Naunton M, Peterson GM, Deeks LS, Young H, Kosari S. We have had a gutful: The need for deprescribing proton pump inhibitors. J Clin Pharm Ther. 2018;43(1):65-72. doi:10.1111/jcpt.12613

11. Pettit SM, Mikhail D, Feuerstein M. Systematic review of interventions that improve provider compliance to imaging guidelines for prostate cancer. Review. Can Urol Assoc J. Sep 2022;16(9):E490-E495. doi:10.5489/cuaj.7638

12. Reen GK, Bailey J, Maughan DL, Vincent C. Systematic review of interventions to improve constant observation on adult inpatient psychiatric wards. Int J Ment Health Nurs. Jun 2020;29(3):372-386. doi:10.1111/inm.12696

13. Tonkin-Crine S, Yardley L, Little P. Antibiotic prescribing for acute respiratory tract infections in primary care: a systematic review and meta-ethnography. J Antimicrob Chemother. Oct 2011;66(10):2215-23. doi:10.1093/jac/dkr279

14. Torloni MR, Brizuela V, Betran AP. Mass media campaigns to reduce unnecessary caesarean sections: a systematic review. BMJ glob. 2020;5(2):e001935. doi:10.1136/bmjgh-2019-001935

15. Vest JR, Jung HY, Ostrovsky A, Das LT, McGinty GB. Image Sharing Technologies and Reduction of Imaging Utilization: A Systematic Review and Meta-analysis. J. Dec 2015;12(12 Pt B):1371-1379.e3. doi:10.1016/j.jacr.2015.09.014

16. Wang T, Baskin AS, Dossett LA. Deimplementation of the Choosing Wisely Recommendations for Low-Value Breast Cancer Surgery: A Systematic Review. JAMA Surg. 08 01 2020;155(8):759-770. doi:10.1001/jamasurg.2020.0322

## Ineligible outcomes (39)

1. Albarqouni L, Palagama S, Chai J, et al. Overuse of medications in low- and middle-income countries: a scoping review. Bull World Health Organ. Jan 01 2023;101(1):36-61D. doi:10.2471/BLT.22.288293

2. Asamoah-Boaheng M, Badejo OA, Bell LV, et al. Interventions to Influence Opioid Prescribing Practices for Chronic Noncancer Pain: A Systematic Review and Meta-Analysis. Am J Prev Med. 2021;60(1):e15-e26. doi:10.1016/j.amepre.2020.07.012

3. Bain A, Hasan SS, Babar ZU. Interventions to improve insulin prescribing practice for people with diabetes in hospital: a systematic review. Diabet Med. 08 2019;36(8):948-960. doi:10.1111/dme.13982

4. Bourcier E, Korb-Savoldelli V, Hejblum G, Fernandez C, Hindlet P. A systematic review of regulatory and educational interventions to reduce the burden associated with the prescriptions of sedative-hypnotics in adults treated for sleep disorders. PLoS ONE. 2018;13(1):e0191211. doi:10.1371/journal.pone.0191211

5. Cadogan CA, Dharamshi R, Fitzgerald S, Corish CA, Castro PD, Ryan C. A systematic scoping review of interventions to improve appropriate prescribing of oral nutritional supplements in primary care. Clin Nutr. 03 2020;39(3):654-663. doi:10.1016/j.clnu.2019.03.003

6. Conombo B, Guertin JR, Tardif PA, et al. Economic Evaluation of In-Hospital Clinical Practices in Acute Injury Care: A Systematic Review. Value Health. 05 2022;25(5):844-854. doi:10.1016/j.jval.2021.10.018

7. Cresswell K, Mozaffar H, Shah S, Sheikh A. Approaches to promoting the appropriate use of antibiotics through hospital electronic prescribing systems: a scoping review. The International journal of pharmacy practice. 2017;25(1):5-17. doi:10.1111/ijpp.12274

8. D'Hulster E, De Burghgraeve T, Luyten J, Verbakel JY. Cost-effectiveness of point-of-care interventions to tackle inappropriate prescribing of antibiotics in high- and middle-income countries: a systematic review. J Antimicrob Chemother. Apr 03 2023;78(4):893-912. doi:10.1093/jac/dkad021

9. Delvaux N, Van Thienen K, Heselmans A, de Velde SV, Ramaekers D, Aertgeerts B. The Effects of Computerized Clinical Decision Support Systems on Laboratory Test Ordering: A Systematic Review. Arch Pathol Lab Med. Apr 2017;141(4):585-595. doi:10.5858/arpa.2016-0115-RA

10. Ellen ME, Wilson MG, Velez M, et al. Addressing overuse of health services in health systems: a critical interpretive synthesis. Health Res Policy Syst. Jun 15 2018;16(1):48. doi:10.1186/s12961-018-0325-x

11. Elshaug AG, Watt AM, Mundy L, Willis CD. Over 150 potentially low-value health care practices: an Australian study. The Medical journal of Australia. 2012;197(10):556-60.

12. Frazer A, Rowland J, Mudge A, Barras M, Martin J, Donovan P. Systematic review of interventions to improve safety and quality of anticoagulant prescribing for therapeutic indications for hospital inpatients. Eur J Clin Pharmacol. Dec 2019;75(12):1645-1657. doi:10.1007/s00228-019-02752-8

13. Gangannagaripalli J, Porter I, Davey A, et al. STOPP/START interventions to improve medicines management for people aged 65 years and over: a realist synthesis. Health Services and Delivery Research. 2021.

14. Hall A, Richmond H, Pike A, et al. What behaviour change techniques have been used to improve adherence to evidence-based low back pain imaging? Implementation science : IS. 2021;16(1):68. doi:10.1186/s13012-021-01136-w

15. Hild S, Johanet M, Valenza A, et al. Quality of decision aids developed for women at average risk of breast cancer eligible for mammographic screening: Systematic review and assessment according to the international patient decision aid standards instrument. Cancer. 2020;126(12):2765-2774. doi:10.1002/cncr.32858

16. Ilic D, Jammal W, Chiarelli P, et al. Assessing the effectiveness of decision AIDS for decision making in prostate cancer testing: A systematic review. Article. Psychooncology. 2015;24(10):1303-1315. doi:10.1002/pon.3815

17. Ivlev I, Hickman EN, McDonagh MS, Eden KB. Use of patient decision aids increased younger women's reluctance to begin screening mammography: a systematic review and meta-analysis. J Gen Intern Med. 2017;32(7):1-10. doi:10.1007/s11606-017-4027-9

18. Johansson T, Abuzahra ME, Keller S, et al. Impact of strategies to reduce polypharmacy on clinically relevant endpoints: a systematic review and meta-analysis. Br J Clin Pharmacol. 08 2016;82(2):532-48. doi:10.1111/bcp.12959

19. Karanika S, Paudel S, Grigoras C, Kalbasi A, Mylonakis E. Systematic Review and Meta-analysis of Clinical and Economic Outcomes from the Implementation of Hospital-Based Antimicrobial Stewardship Programs. Antimicrob Agents Chemother. Aug 2016;60(8):4840-52. doi:10.1128/aac.00825-16

20. Kooda K, Canterbury E, Bellolio F. Impact of Pharmacist-Led Antimicrobial Stewardship on Appropriate Antibiotic Prescribing in the Emergency Department: A Systematic Review and Meta-Analysis. Ann Emerg Med. 04 2022;79(4):374-387. doi:10.1016/j.annemergmed.2021.11.031

21. Lee JQ, Ying K, Lun P, et al. Intervention elements to reduce inappropriate prescribing for older adults with multimorbidity receiving outpatient care: a scoping review. BMJ Open. 08 20 2020;10(8):e039543. doi:10.1136/bmjopen-2020-039543

22. Lin L, Alam P, Fearon E, Hargreaves JR. Public target interventions to reduce the inappropriate use of medicines or medical procedures: a systematic review. Implement Sci. 10 20 2020;15(1):90. doi:10.1186/s13012-020-01018-7

23. Low LF, Fletcher J, Goodenough B, et al. A Systematic Review of Interventions to Change Staff Care Practices in Order to Improve Resident Outcomes in Nursing Homes. PLoS ONE. 2015;10(11):e0140711. doi:10.1371/journal.pone.0140711

24. Main C, Moxham T, Wyatt JC, Kay J, Anderson R, Stein K. Computerised decision support systems in order communication for diagnostic, screening or monitoring test ordering: systematic reviews of the effects and cost-effectiveness of systems. Health Technol Assess. 2010;14(48):1-227. doi:10.3310/hta14480

25. Murphy M, Bennett K, Ryan S, Hughes CM, Lavan AH, Cadogan CA. A systematic scoping review of interventions to optimise medication prescribing and adherence in older adults with cancer. Res Social Adm Pharm. 03 2022;18(3):2392-2402. doi:10.1016/j.sapharm.2021.04.011

26. Nagarajah S, Powis ML, Fazelzad R, Krzyzanowska MK, Kukreti V. Implementation and Impact of Choosing Wisely Recommendations in Oncology. JCO Oncol Pract. 10 2022;18(10):703-712. doi:10.1200/OP.22.00130

27. Page AT, Clifford RM, Potter K, Schwartz D, Etherton-Beer CD. The feasibility and effect of deprescribing in older adults on mortality and health: a systematic review and meta-analysis. Br J Clin Pharmacol. 09 2016;82(3):583-623. doi:10.1111/bcp.12975

28. Pitiriga V, Vrioni G, Saroglou G, Tsakris A. The Impact of Antibiotic Stewardship Programs in Combating Quinolone Resistance: A Systematic Review and Recommendations for More Efficient Interventions. Adv Ther. Apr 2017;34(4):854-865. doi:10.1007/s12325-017-0514-y

29. Riikonen JM, Guyatt GH, Kilpeläinen TP, et al. Decision Aids for Prostate Cancer Screening Choice: A Systematic Review and Meta-analysis. JAMA Intern Med. 2019;179(8):1072-1082. doi:10.1001/jamainternmed.2019.0763

30. Robinson PD, Dupuis LL, Tomlinson G, Phillips B, Greenberg M, Sung L. Strategies facilitating practice change in pediatric cancer: a systematic review. Int J Qual Health Care. Sep 2016;28(4):426-32. doi:10.1093/intqhc/mzw052

31. Roshanov PS, Misra S, Gerstein HC, et al. Computerized clinical decision support systems for chronic disease management: a decision-maker-researcher partnership systematic review. Implement Sci. Aug 3 2011;6:92. doi:10.1186/1748-5908-6-92

32. Saini S, Leung V, Si E, et al. Documenting the indication for antimicrobial prescribing: a scoping review. BMJ Qual Saf. May 12 2022;12:12. doi:10.1136/bmjqs-2021-014582

33. Schuetz P, Wirz Y, Sager R, et al. Procalcitonin to initiate or discontinue antibiotics in acute respiratory tract infections. Cochrane Database Syst Rev. Oct 12 2017;10(10):Cd007498. doi:10.1002/14651858.CD007498.pub3

34. Smith SM, Soubhi H, Fortin M, Hudon C, O'Dowd T. Managing patients with multimorbidity: systematic review of interventions in primary care and community settings. BMJ (Clinical research ed). 2012;345:e5205. doi:10.1136/bmj.e5205

35. Sypes EE, de Grood C, Clement FM, et al. Understanding the public's role in reducing low-value care: a scoping review. Implement Sci. 04 07 2020;15(1):20. doi:10.1186/s13012-020-00986-0

36. Tan EC, Stewart K, Elliott RA, George J. Pharmacist services provided in general practice clinics: a systematic review and meta-analysis. Research in social & administrative pharmacy: RSAP. 2014;10(4):608-22. doi:10.1016/j.sapharm.2013.08.006

37. Volpe A, Finelli A, Gill IS, et al. Rationale for percutaneous biopsy and histologic characterisation of renal tumours. Eur Urol. Sep 2012;62(3):491-504. doi:10.1016/j.eururo.2012.05.009

38. Wubishet BL, Merlo G, Ghahreman-Falconer N, Hall L, Comans T. Economic evaluation of antimicrobial stewardship in primary care: a systematic review and quality assessment. J Antimicrob Chemother. 08 25 2022;77(9):2373-2388. doi:10.1093/jac/dkac185

39. Yu L, Li P, Yang S, et al. Web-based decision aids to support breast cancer screening decisions: systematic review and meta-analysis. Journal of comparative effectiveness research. 2020;9(14):985-1002. doi:10.2217/cer-2020-0052

## Ineligible intervention (89)

1. Alenezi A, Yahyouche A, Paudyal V. Interventions to optimize prescribed medicines and reduce their misuse in chronic non-malignant pain: a systematic review. Eur J Clin Pharmacol. Apr 2021;77(4):467-490. doi:10.1007/s00228-020-03026-4

2. Alldred DP, Kennedy MC, Hughes C, Chen TF, Miller P. Interventions to optimise prescribing for older people in care homes. Cochrane Database Syst Rev. Feb 12 2016;2:CD009095. doi:10.1002/14651858.CD009095.pub3

3. Avery N, McNeilage AG, Stanaway F, et al. Efficacy of interventions to reduce long term opioid treatment for chronic non-cancer pain: systematic review and meta-analysis. Bmj. 04 04 2022;377:e066375. doi:10.1136/bmj-2021-066375

4. Awadalla R, Gnjidic D, Patanwala A, Sakiris M, Penm J. The Effectiveness of Stewardship Interventions to Reduce the Prescribing of Extended-Release Opioids for Acute Pain: A Systematic Review. Pain Med. 10 01 2020;21(10):2401-2411. doi:10.1093/pm/pnaa139

5. Bayoumi I, Balas MA, Handler SM, Dolovich L, Hutchison B, Holbrook A. The effectiveness of computerized drug-lab alerts: a systematic review and meta-analysis. Int J Med Inf. 2014;83(6):406-15. doi:10.1016/j.ijmedinf.2014.03.003

6. Beaudoin FL, Banerjee GN, Mello MJ. State-level and system-level opioid prescribing policies: The impact on provider practices and overdose deaths, a systematic review. J Opioid Manag. May-Jun 2016;12(2):109-18. doi:10.5055/jom.2016.0322

7. Bloomfield H, Linsky A, Bolduc J, et al. Deprescribing for Older Veterans: A Systematic Review. VA Evidence-based Synthesis Program Reports. 2019.

8. Bloomfield HE, Greer N, Linsky AM, et al. Deprescribing for Community-Dwelling Older Adults: a Systematic Review and Meta-analysis. J Gen Intern Med. 11 2020;35(11):3323-3332. doi:10.1007/s11606-020-06089-2

9. Boghossian TA, Rashid FJ, Thompson W, et al. Deprescribing versus continuation of chronic proton pump inhibitor use in adults. Cochrane Database Syst Rev. Mar 16 2017;3:CD011969. doi:10.1002/14651858.CD011969.pub2

10. Borab ZM, Lanni MA, Tecce MG, Pannucci CJ, Fischer JP. Use of Computerized Clinical Decision Support Systems to Prevent Venous Thromboembolism in Surgical Patients: A Systematic Review and Meta-analysis. JAMA Surg. 2017;152(7):638-645. doi:10.1001/jamasurg.2017.0131

11. Brennan N, Mattick K. A systematic review of educational interventions to change behaviour of prescribers in hospital settings, with a particular emphasis on new prescribers. Br J Clin Pharmacol. 2013;75(2):359-72. doi:10.1111/j.1365-2125.2012.04397.x

12. Burry L, Turner J, Morgenthaler T, et al. Addressing Barriers to Reducing Prescribing and Implementing Deprescribing of Sedative-Hypnotics in Primary Care. Ann Pharmacother. Apr 2022;56(4):463-474. doi:10.1177/10600280211033022

13. Buzancic I, Kummer I, Drzaic M, Ortner Hadziabdic M. Community-based pharmacists' role in deprescribing: A systematic review. Br J Clin Pharmacol. 02 2022;88(2):452-463. doi:10.1111/bcp.14947

14. Cardona M, Stehlik P, Fawzy P, et al. Effectiveness and sustainability of deprescribing for hospitalized older patients near end of life: a systematic review. Expert Opin Drug Saf. Jan 2021;20(1):81-91. doi:10.1080/14740338.2021.1853704

15. Carter M, Abutheraa N, Ivers N, et al. Audit and feedback interventions involving pharmacists to influence prescribing behaviour in general practice: a systematic review and meta-analysis. Fam Pract. 2023;doi:10.1093/fampra/cmac150

16. Clarkson L, Hart L, Lam AK, Khoo TK. Reducing inappropriate polypharmacy for older patients at specialist outpatient clinics: a systematic review. Curr Med Res Opin. 04 2023;39(4):545-554. doi:10.1080/03007995.2023.2185390

17. Clyne B, Fitzgerald C, Quinlan A, et al. Interventions to Address Potentially Inappropriate Prescribing in Community-Dwelling Older Adults: A Systematic Review of Randomized Controlled Trials. J Am Geriatr Soc. 06 2016;64(6):1210-22. doi:10.1111/jgs.14133

18. Cooper JA, Cadogan CA, Patterson SM, et al. Interventions to improve the appropriate use of polypharmacy in older people: a Cochrane systematic review. BMJ Open. Dec 09 2015;5(12):e009235. doi:10.1136/bmjopen-2015-009235

19. Crayton E, Richardson M, Fuller C, et al. Interventions to improve appropriate antibiotic prescribing in long-term care facilities: a systematic review. BMC geriatr. 07 09 2020;20(1):237. doi:10.1186/s12877-020-01564-1

20. Croke A, Cardwell K, Clyne B, Moriarty F, McCullagh L, Smith SM. The effectiveness and cost of integrating pharmacists within general practice to optimize prescribing and health outcomes in primary care patients with polypharmacy: a systematic review. BMC Prim Care. 02 06 2023;24(1):41. doi:10.1186/s12875-022-01952-z

21. Dalton K, O'Brien G, O'Mahony D, Byrne S. Computerised interventions designed to reduce potentially inappropriate prescribing in hospitalised older adults: a systematic review and meta-analysis. Age Ageing. 09 01 2018;47(5):670-678. doi:10.1093/ageing/afy086

22. De Oliveira GS, Castro-Alves LJ, Kendall MC, McCarthy R. Effectiveness of Pharmacist Intervention to Reduce Medication Errors and Health-Care Resources Utilization After Transitions of Care: A Meta-analysis of Randomized Controlled Trials. Journal of patient safety. 2017;doi:10.1097/PTS.0000000000000283

23. Derzon JH, Clarke N, Alford A, Gross I, Shander A, Thurer R. Reducing red blood cell transfusion in orthopedic and cardiac surgeries with Antifibrinolytics: A laboratory medicine best practice systematic review and meta-analysis. Clin Biochem. Sep 2019;71:1-13. doi:10.1016/j.clinbiochem.2019.06.015

24. Earl TR, Katapodis ND, Schneiderman SR, Shoemaker-Hunt SJ. Using Deprescribing Practices and the Screening Tool of Older Persons' Potentially Inappropriate Prescriptions Criteria to Reduce Harm and Preventable Adverse Drug Events in Older Adults. Journal of Patient Safety. 2020:23-35.

25. Faulkner L, Hughes CM, Barry HE. Interventions to improve medicines optimisation in older people with frailty in primary care: a systematic review. Int J Pharm Pract. Aug 09 2022;30(4):297-304. doi:10.1093/ijpp/riac036

26. Field RA, Fritz Z, Baker A, Grove A, Perkins GD. Systematic review of interventions to improve appropriate use and outcomes associated with do-not-attempt-cardiopulmonary-resuscitation decisions. Resuscitation. Nov 2014;85(11):1418-31. doi:10.1016/j.resuscitation.2014.08.024

27. Forsetlund L, Eike MC, Gjerberg E, Vist GE. Effect of interventions to reduce potentially inappropriate use of drugs in nursing homes: a systematic review of randomised controlled trials. BMC geriatr. Apr 17 2011;11:16. doi:10.1186/1471-2318-11-16

28. French SD, Green S, Buchbinder R, Barnes H. Interventions for improving the appropriate use of imaging in people with musculoskeletal conditions. Cochrane Database Syst Rev. Jan 20 2010;(1):CD006094. doi:10.1002/14651858.CD006094.pub2

29. Furlan AD, Carnide N, Irvin E, et al. A systematic review of strategies to improve appropriate use of opioids and to reduce opioid use disorder and deaths from prescription opioids. Can J Pain. 2018;2(1):218-235. doi:10.1080/24740527.2018.1479842

30. Gillaizeau F, Chan E, Trinquart L, et al. Computerized advice on drug dosage to improve prescribing practice. Cochrane Database Syst Rev. Nov 12 2013;(11):CD002894. doi:10.1002/14651858.CD002894.pub3

31. Gorman A, Rankin A, Hughes C, O'Dwyer M, Ryan C. Theoretically derived interventions aimed at improving appropriate polypharmacy in primary care: A systematic review. Explor Res Clin Soc Pharm. Sep 2022;7:100166. doi:10.1016/j.rcsop.2022.100166

32. Hansen CR, O'Mahony D, Kearney PM, et al. Identification of behaviour change techniques in deprescribing interventions: a systematic review and meta-analysis. Br J Clin Pharmacol. 12 2018;84(12):2716-2728. doi:10.1111/bcp.13742

33. Hernandez-Prats C, Lopez-Pintor E, Lumbreras B. Pharmacist-led intervention on the reduction of inappropriate medication use in patients with heart failure: A systematic review of randomized trials and non-randomized intervention studies. Res Social Adm Pharm. 05 2022;18(5):2748-2756. doi:10.1016/j.sapharm.2021.06.023

34. Hill-Taylor B, Walsh KA, Stewart S, Hayden J, Byrne S, Sketris IS. Effectiveness of the STOPP/START (Screening Tool of Older Persons' potentially inappropriate Prescriptions/Screening Tool to Alert doctors to the Right Treatment) criteria: systematic review and meta-analysis of randomized controlled studies. J Clin Pharm Ther. Apr 2016;41(2):158-69. doi:10.1111/jcpt.12372

35. Iankowitz N, Dowden M, Palomino S, Uzokwe H, Worral P. The effectiveness of computer system tools on potentially inappropriate medications ordered at discharge for adults older than 65 years of age: a systematic review. JBI Libr Syst Rev. 2012;10(13):798-831.

36. Jutkowitz E, Brasure M, Fuchs E, et al. Care-Delivery Interventions to Manage Agitation and Aggression in Dementia Nursing Home and Assisted Living Residents: A Systematic Review and Meta-analysis. J Am Geriatr Soc. Mar 2016;64(3):477-88. doi:10.1111/jgs.13936

37. Kamarudin G, Penm J, Chaar B, Moles R. Educational interventions to improve prescribing competency: A systematic review. BMJ Open. 2013;3(8)e003291. doi:10.1136/bmjopen-2013-003291

38. Kausner Y, Rover C, Heinz J, et al. Reducing antibiotic use in uncomplicated urinary tract infections in adult women: a systematic review and individual participant data meta-analysis. Clin Microbiol Infect. Dec 2022;28(12):1558-1566. doi:10.1016/j.cmi.2022.06.017

39. Kua CH, Mak VSL, Huey Lee SW. Health Outcomes of Deprescribing Interventions Among Older Residents in Nursing Homes: A Systematic Review and Meta-analysis. J Am Med Dir Assoc. 03 2019;20(3):362-372.e11. doi:10.1016/j.jamda.2018.10.026

40. Kurczewska-Michalak M, Lewek P, Jankowska-Polanska B, et al. Polypharmacy Management in the Older Adults: A Scoping Review of Available Interventions. Front Pharmacol. 2021;12:734045. doi:10.3389/fphar.2021.734045

41. Lainer M, Mann E, Sönnichsen A. Information technology interventions to improve medication safety in primary care: a systematic review. International journal for quality in health care: journal of the International Society for Quality in Health Care / ISQua. 2013 2013;25(5):590-598. doi:10.1093/intqhc/mzt043

42. Lee IH, Bloor K, Hewitt C, Maynard A. International experience in controlling pharmaceutical expenditure: influencing patients and providers and regulating industry - a systematic review. Journal of health services research & policy. 2015;20(1):52-59. doi:10.1177/1355819614545675

43. Li H, Luo YF, Blackwell TS, Xie CM. Meta-analysis and systematic review of procalcitonin-guided therapy in respiratory tract infections. Antimicrobial agents and chemotherapy. 2011;55(12):5900-6. doi:10.1128/AAC.00335-11

44. Marasinghe KM. Computerised clinical decision support systems to improve medication safety in long-term care homes: a systematic review. BMJ Open. May 12 2015;5(5):e006539. doi:10.1136/bmjopen-2014-006539

45. Mathieson S, Maher CG, Ferreira GE, et al. Deprescribing Opioids in Chronic Non-cancer Pain: Systematic Review of Randomised Trials. Drugs. Oct 2020;80(15):1563-1576. doi:10.1007/s40265-020-01368-y

46. McKibbon KA, Lokker C, Handler SM, et al. Enabling medication management through health information technology (Health IT). Evidence report/technology assessment. 2011;(201):1-951.

47. McKibbon KA, Lokker C, Handler SM, et al. The effectiveness of integrated health information technologies across the phases of medication management: a systematic review of randomized controlled trials. Journal of the American Medical Informatics Association: JAMIA. 2012;19(1):22-30. doi:10.1136/amiajnl-2011-000304

48. Meid AD, Lampert A, Burnett A, Seidling HM, Haefeli WE. The impact of pharmaceutical care interventions for medication underuse in older people: a systematic review and meta-analysis. Br J Clin Pharmacol. Oct 2015;80(4):768-76. doi:10.1111/bcp.12657

49. Monteiro L, Maricoto T, Solha I, Ribeiro-Vaz I, Martins C, Monteiro-Soares M. Reducing Potentially Inappropriate Prescriptions for Older Patients Using Computerized Decision Support Tools: Systematic Review. J Med Internet Res. 11 14 2019;21(11):e15385. doi:10.2196/15385

50. Moride Y, Lemieux-Uresandi D, Castillon G, et al. A Systematic Review of Interventions and Programs Targeting Appropriate Prescribing of Opioids. Pain physician. 05 2019;22(3):229-240.

51. Mucherino S, Casula M, Galimberti F, et al. The Effectiveness of Interventions to Evaluate and Reduce Healthcare Costs of Potentially Inappropriate Prescriptions among the Older Adults: A Systematic Review. Int J Environ Res Public Health. 05 31 2022;19(11):31. doi:10.3390/ijerph19116724

52. Nakham A, Myint PK, Bond CM, Newlands R, Loke YK, Cruickshank M. Interventions to Reduce Anticholinergic Burden in Adults Aged 65 and Older: A Systematic Review. J Am Med Dir Assoc. 02 2020;21(2):172-180.e5. doi:10.1016/j.jamda.2019.06.001

53. Neville HL, Granter C, Adibi P, Belliveau J, Isenor JE, Bowles SK. Interventions to reduce benzodiazepine and sedative-hypnotic drug use in acute care hospitals: A scoping review. Res Social Adm Pharm. 05 2022;18(5):2874-2886. doi:10.1016/j.sapharm.2021.07.004

54. Nickerson K, Lieschke G, Rajappa H, Smith A, Inder KJ. A scoping review of outpatient interventions to support the reduction of prescription opioid medication for chronic non cancer pain. J Clin Nurs. Dec 2022;31(23-24):3368-3389. doi:10.1111/jocn.16235

55. Nkansah N, Mostovetsky O, Yu C, et al. Effect of outpatient pharmacists' non‐dispensing roles on patient outcomes and prescribing patterns. Cochrane database of systematic reviews (Online). 2010;7(7):CD000336. doi:10.1002/14651858.CD000336.pub2

56. Oktora MP, Kerr KP, Hak E, Denig P. Rates, determinants and success of implementing deprescribing in people with type 2 diabetes: A scoping review. Diabet Med. 02 2021;38(2):e14408. doi:10.1111/dme.14408

57. Orelio CC, Heus P, Kroese-van Dieren JJ, Spijker R, van Munster BC, Hooft L. Reducing Inappropriate Proton Pump Inhibitors Use for Stress Ulcer Prophylaxis in Hospitalized Patients: Systematic Review of De-Implementation Studies. J Gen Intern Med. 07 2021;36(7):2065-2073. doi:10.1007/s11606-020-06425-6

58. Ospina MB, Taenzer P, Rashiq S, et al. A systematic review of the effectiveness of knowledge translation interventions for chronic noncancer pain management. Pain research & management. 2013;18(6):e129-41. doi:10.1155/2013/120784

59. Ostini R, Jackson C, Hegney D, Tett SE. How is medication prescribing ceased? A systematic review. Med Care. 2011;49(1):24-36. doi:10.1097/MLR.0b013e3181ef9a7e

60. Page N, Baysari MT, Westbrook JI. A systematic review of the effectiveness of interruptive medication prescribing alerts in hospital CPOE systems to change prescriber behavior and improve patient safety. Int J Med Inf. 09 2017;105:22-30. doi:10.1016/j.ijmedinf.2017.05.011

61. Parker J, Gupta S, Torkington J, Dolwani S. Multidisciplinary decision-making strategies may reduce the need for secondary surgery in complex colonic polyps - A systematic review and pooled analysis. Colorectal Dis. 12 2021;23(12):3101-3112. doi:10.1111/codi.15901

62. Polisena J, Clifford T, Elshaug AG, Mitton C, Russell E, Skidmore B. Case studies that illustrate disinvestment and resource allocation decision-making processes in health care: a systematic review. Int J Technol Assess Health Care. Apr 2013;29(2):174-84. doi:10.1017/S0266462313000068

63. Rankin A, Cadogan CA, Patterson SM, et al. Interventions to improve the appropriate use of polypharmacy for older people. Cochrane Database Syst Rev. 09 03 2018;9:CD008165. doi:10.1002/14651858.CD008165.pub4

64. Reeve E, Ong M, Wu A, Jansen J, Petrovic M, Gnjidic D. A systematic review of interventions to deprescribe benzodiazepines and other hypnotics among older people. Eur J Clin Pharmacol. Aug 2017;73(8):927-935. doi:10.1007/s00228-017-2257-8

65. Rhodes E, Wilson M, Robinson A, Hayden JA, Asbridge M. The effectiveness of prescription drug monitoring programs at reducing opioid-related harms and consequences: a systematic review. BMC Health Serv Res. Nov 01 2019;19(1):784. doi:10.1186/s12913-019-4642-8

66. Ribeiro PRS, Schlindwein AD. Benzodiazepine deprescription strategies in chronic users: a systematic review. Fam Pract. 09 25 2021;38(5):684-693. doi:10.1093/fampra/cmab017

67. Riordan DO, Walsh KA, Galvin R, Sinnott C, Kearney PM, Byrne S. The effect of pharmacist-led interventions in optimising prescribing in older adults in primary care: A systematic review. SAGE Open Med. 2016;4:2050312116652568. doi:10.1177/2050312116652568

68. Robertson J, Walkom E, Pearson SA, Hains I, Williamsone M, Newby D. The impact of pharmacy computerised clinical decision support on prescribing, clinical and patient outcomes: a systematic review of the literature. The International journal of pharmacy practice. 2010;18(2):69-87. doi:10.1211/ijpp/18.02.0002

69. Rodrigues DA, Placido AI, Mateos-Campos R, Figueiras A, Herdeiro MT, Roque F. Effectiveness of Interventions to Reduce Potentially Inappropriate Medication in Older Patients: A Systematic Review. Front Pharmacol. 2021;12:777655. doi:10.3389/fphar.2021.777655

70. Romano S, Figueira D, Teixeira I, Perelman J. Deprescribing Interventions among Community-Dwelling Older Adults: A Systematic Review of Economic Evaluations. Pharmacoeconomics. 03 2022;40(3):269-295. doi:10.1007/s40273-021-01120-8

71. Roshanov PS, You JJ, Dhaliwal J, et al. Can computerized clinical decision support systems improve practitioners' diagnostic test ordering behavior? A decision-maker-researcher partnership systematic review. Implement Sci. Aug 03 2011;6:88. doi:10.1186/1748-5908-6-88

72. Saeed D, Carter G, Parsons C. Interventions to improve medicines optimisation in frail older patients in secondary and acute care settings: a systematic review of randomised controlled trials and non-randomised studies. Int J Clin Pharm. Feb 2022;44(1):15-26. doi:10.1007/s11096-021-01354-8

73. Salahudeen MS, Alfahmi A, Farooq A, et al. Effectiveness of Interventions to Improve the Anticholinergic Prescribing Practice in Older Adults: A Systematic Review. J. Clin. Med. Jan 28 2022;11(3):28. doi:10.3390/jcm11030714

74. Schuetz P, Chiappa V, Briel M, Greenwald JL. Procalcitonin algorithms for antibiotic therapy decisions: a systematic review of randomized controlled trials and recommendations for clinical algorithms. Archives of internal medicine. 2011;171(15):1322-31. doi:10.1001/archinternmed.2011.318

75. Shafiq N, Gautam V, Pandey AK, et al. A meta-analysis to assess usefulness of procalcitonin-guided antibiotic usage for decision making. The Indian journal of medical research. 2017;146(5):576-584. doi:10.4103/ijmr.IJMR_613_15

76. Shawaqfeh B, Hughes CM, McGuinness B, Barry HE. A systematic review of interventions to reduce anticholinergic burden in older people with dementia in primary care. Int J Geriatr Psychiatry. Apr 27 2022;37(6):27. doi:10.1002/gps.5722

77. Sheehan R, Strydom A, Brown E, Marston L, Hassiotis A. Association of Focused Medication Review With Optimization of Psychotropic Drug Prescribing: A Systematic Review and Meta-analysis. JAMA netw. 2018;1(6):e183750-e183750. doi:10.1001/jamanetworkopen.2018.3750

78. Shrestha S, Poudel A, Steadman K, Nissen L. Outcomes of deprescribing interventions in older patients with life-limiting illness and limited life expectancy: A systematic review. Br J Clin Pharmacol. 10 2020;86(10):1931-1945. doi:10.1111/bcp.14113

79. Silvestri MT, Bongiovanni TR, Glover JG, Gross CP. Impact of price display on provider ordering: A systematic review. J Hosp Med. Jan 2016;11(1):65-76. doi:10.1002/jhm.2500

80. Singh-Franco D, Mastropietro DR, Metzner M, et al. Impact of pharmacy-supported interventions on proportion of patients receiving non-indicated acid suppressive therapy upon discharge: A systematic review and meta-analysis. PLoS ONE. 2020;15(12):e0243134. doi:10.1371/journal.pone.0243134

81. Soni NJ, Samson DJ, Galaydick JL, et al. Procalcitonin-guided antibiotic therapy: a systematic review and meta-analysis. Journal of hospital medicine: an official publication of the Society of Hospital Medicine. 2013;8(9):530-40. doi:10.1002/jhm.2067

82. Stultz JS, Nahata MC. Computerized clinical decision support for medication prescribing and utilization in pediatrics. Journal of the American Medical Informatics Association: JAMIA. 2012;19(6):942-53. doi:10.1136/amiajnl-2011-000798

83. Tawadrous D, Shariff SZ, Haynes RB, Iansavichus AV, Jain AK, Garg AX. Use of clinical decision support systems for kidney-related drug prescribing: a systematic review. Am J Kidney Dis. 2011;58(6):903-14. doi:10.1053/j.ajkd.2011.07.022

84. Tesfaye WH, Castelino RL, Wimmer BC, Zaidi STR. Inappropriate prescribing in chronic kidney disease: A systematic review of prevalence, associated clinical outcomes and impact of interventions. Int J Clin Pract. Jul 2017;71(7)doi:10.1111/ijcp.12960

85. Thaker A, Navadeh S, Gonzales H, Malekinejad M. Effectiveness of Policies on Reducing Exposure to Ionizing Radiation From Medical Imaging: A Systematic Review. J Am Coll Radiol. Dec 2015;12(12 Pt B):1434-45. doi:10.1016/j.jacr.2015.06.033

86. Thillainadesan J, Gnjidic D, Green S, Hilmer SN. Impact of Deprescribing Interventions in Older Hospitalised Patients on Prescribing and Clinical Outcomes: A Systematic Review of Randomised Trials. Drugs Aging. Apr 2018;35(4):303-319. doi:10.1007/s40266-018-0536-4

87. Tjia J, Velten SJ, Parsons C, Valluri S, Briesacher BA. Studies to reduce unnecessary medication use in frail older adults: a systematic review. Drugs Aging. May 2013;30(5):285-307. doi:10.1007/s40266-013-0064-1

88. Tzortziou Brown V, Underwood M, Mohamed N, Westwood O, Morrissey D. Professional interventions for general practitioners on the management of musculoskeletal conditions. Cochrane Database Syst Rev. 2016;5(5):CD007495. doi:10.1002/14651858.CD007495.pub2

89. Walsh KA, O'Riordan D, Kearney PM, Timmons S, Byrne S. Improving the appropriateness of prescribing in older patients: a systematic review and meta-analysis of pharmacists' interventions in secondary care. Age Ageing. Mar 2016;45(2):201-9. doi:10.1093/ageing/afv190

# eTable 3: Assessment of the confidence in the results (according to AMSTAR 2)

| **Author Year** | **1. PICO?** | **2. Protocol?** | **3. STUDY DESIGN?** | **4. SEARCH?** | **5. DUPLIC. SELECTION?** | **6. DUPL. EXTRACTION?** | **7. LIST EXCL. STUDIES?** | **8. DESCRIPTION?** | **9.a. ROB RCTs?** | **9.b. ROB NRSI?** | **10. FUNDING?** | **11.a. MA RCTs?** | **11.b. MA NRSI?** | **12. IMPACT ROB MA?** | **13. IMPACT ROB?** | **14. HETEROGENEITY?** | **15. PUBLICATION BIAS?** | **16. CONFLICT?** | **Overall confidence** |
| --- | --- | --- | --- | --- | --- | --- | --- | --- | --- | --- | --- | --- | --- | --- | --- | --- | --- | --- | --- |
| **Included SRs and extracted** | | | | | | | | | | | | | | | | | | | |
| Badreldin 2023 | Y | Y | N | P-Y | Y | Y | N | P-Y | Y | Y | N | N-MA | N-MA | N-MA | N | N | N-MA | Y | LOW |
| Baptista 2018 | Y | N | N | P-Y | Y | Y | Y | P-Y | Y | RCT | N | Y | N-MA | Y | Y | Y | Y | Y | LOW |
| Belavy 2022 | Y | P-Y | Y | P-Y | Y | Y | Y | Y | Y | RCT | Y | Y | N-MA | N | Y | Y | Y | Y | MOD |
| Birkenhäger-Gillesse 2018 | N | N | N | P-Y | Y | Y | N | P-Y | Y | Y | N | N-MA | N-MA | N-MA | Y | Y | N-MA | Y | LOW |
| Chen 2018 | Y | Y | Y | P-Y | Y | Y | Y | Y | Y | Y | Y | N-MA | N-MA | N-MA | Y | Y | N-MA | Y | HIGH |
| Coxeter 2015 | N | P-Y | N | P-Y | Y | Y | Y | Y | Y | RCT | Y | Y | N-MA | Y | Y | Y | N-MA | Y | MOD |
| Daoust 2022 | Y | P-Y | N | P-Y | Y | Y | N | N | Y | Y | N | N-MA | Y | Y | Y | Y | Y | Y | MOD |
| Davey 2017 | Y | Y | Y | Y | Y | Y | Y | Y | Y | Y | Y | Y | Y | Y | Y | Y | Y | Y | HIGH |
| deBont 2015 | N | N | N | P-Y | Y | N | N | Y | Y | RCT | N | N-MA | N-MA | N-MA | Y | N | N-MA | Y | LOW |
| Doan 2014 | Y | Y | N | P-Y | Y | Y | Y | Y | Y | RCT | N | Y | N-MA | Y | Y | Y | N-MA | Y | HIGH |
| Dunn 2021 | N | P-Y | N | P-Y | Y | Y | Y | N | NRSI | Y | N | N-MA | N-MA | N-MA | N | Y | N-MA | Y | LOW |
| Dunne 2022 | Y | Y | N | P-Y | Y | Y | N | N | Y | Y | N | N-MA | N-MA | N-MA | Y | Y | Y | Y | MOD |
| Fleming 2013 | Y | N | N | P-Y | Y | N | Y | P-Y | Y | RCT | N | N-MA | N-MA | N-MA | Y | Y | N-MA | Y | LOW |
| Foster 2020 | Y | Y | Y | P-Y | Y | Y | Y | P-Y | N | P-Y | Y | N-MA | N-MA | N-MA | Y | Y | N-MA | Y | LOW |
| Kjelle 2021 | N | P-Y | N | P-Y | Y | Y | Y | N | Y | Y | N | N-MA | N-MA | N-MA | N | Y | N-MA | Y | LOW |
| Kobewka 2015 | Y | P-Y | N | P-Y | Y | N | N | N | Y | Y | N | N-MA | N-MA | N-MA | Y | Y | N-MA | Y | MOD |
| Lane 2018 | Y | Y | N | P-Y | N | Y | N | Y | Y | Y | N | N-MA | N-MA | N-MA | N | N | N-MA | Y | LOW |
| Lim 2020 | Y | Y | Y | P-Y | Y | Y | N | P-Y | NRSI | Y | N | N-MA | N-MA | N-MA | N | Y | N-MA | Y | LOW |
| MartinezGonzalez 2020 | Y | P-Y | N | P-Y | Y | Y | Y | Y | Y | RCT | Y | Y | N-MA | Y | Y | Y | Y | Y | HIGH |
| Mokhar 2018 | Y | P-Y | N | P-Y | Y | Y | N | P-Y | Y | RCT | N | N-MA | N-MA | N-MA | N | N | N-MA | Y | LOW |
| Mortazhejri 2020 | Y | Y | N | P-Y | Y | Y | Y | P-Y | Y | Y | Y | Y | Y | Y | Y | Y | Y | Y | HIGH |
| Nabovati 2021 | Y | N | N | P-Y | Y | Y | N | Y | P-Y | RCT | N | N-MA | N-MA | N-MA | Y | N | N-MA | Y | LOW |
| Nair 2021 | Y | Y | Y | P-Y | Y | Y | N | P-Y | Y | N | N | N-MA | N-MA | N-MA | Y | N | N-MA | N | MOD |
| Nguyen 2019 | Y | Y | N | P-Y | Y | Y | N | P-Y | Y | RCT | N | N-MA | N-MA | N-MA | Y | Y | N-MA | Y | MOD |
| O'Sullivan 2016 | Y | P-Y | N | Y | Y | Y | Y | Y | Y | RCT | Y | N-MA | N-MA | N-MA | Y | Y | N-MA | Y | HIGH |
| Phinn 2023 | Y | P-Y | Y | P-Y | Y | N | N | N | Y | Y | N | N-MA | N-MA | N-MA | Y | N | N-MA | Y | MOD |
| Raban 2023 | N | N | Y | P-Y | Y | Y | N | N | Y | Y | N | N-MA | N-MA | N-MA | Y | N | N-MA | Y | LOW |
| Rajar 2020 | Y | P-Y | N | P-Y | Y | N | Y | P-Y | Y | Y | N | N-MA | N-MA | N-MA | Y | Y | N-MA | Y | MOD |
| Ralston 2014 | Y | P-Y | N | P-Y | Y | Y | N | P-Y | NRSI | N | N | N-MA | Y | N | Y | Y | N | Y | LOW |
| Rietbergen 2020 | Y | Y | N | P-Y | Y | Y | N | N | Y | Y | N | Y | N-MA | N | Y | Y | Y | Y | MOD |
| Siachalinga 2022 | Y | N | N | P-Y | Y | N | N | P-Y | Y | Y | N | N-MA | Y | Y | Y | Y | N | Y | LOW |
| Smedemark 2022 | Y | P-Y | N | Y | Y | Y | Y | Y | Y | RCT | Y | Y | N-MA | Y | Y | Y | Y | Y | MOD |
| Spurling 2017 | Y | Y | N | Y | Y | Y | Y | Y | Y | RCT | Y | Y | N-MA | Y | Y | Y | Y | Y | HIGH |
| Sypes 2020 | N | N | Y | P-Y | Y | Y | N | P-Y | Y | Y | N | Y | Y | Y | Y | Y | Y | Y | HIGH |
| Takada 2020 | N | N | N | P-Y | Y | Y | N | N | Y | RCT | N | N-MA | N-MA | N-MA | N | Y | N-MA | Y | LOW |
| ThompsonCoon 2014 | Y | P-Y | N | P-Y | Y | Y | N | Y | Y | Y | N | N-MA | N-MA | N-MA | Y | Y | N-MA | Y | MOD |
| VanDijck 2018 | Y | P-Y | N | Y | Y | Y | N | P-Y | Y | Y | N | N-MA | N-MA | N-MA | Y | Y | N-MA | Y | MOD |
| Vodicka 2013 | Y | N | N | P-Y | N | Y | N | Y | Y | P-Y | N | N-MA | N-MA | N-MA | Y | N | N-MA | Y | LOW |
| Xie 2022 | Y | P-Y | Y | P-Y | Y | Y | N | Y | Y | RCT | N | N-MA | N-MA | N-MA | Y | Y | N-MA | Y | MOD |
| Xiong 2018 | Y | Y | N | P-Y | Y | Y | N | N | Y | Y | N | N-MA | N-MA | N-MA | Y | N | N-MA | Y | MOD |
| Xu 2021 | Y | P-Y | N | P-Y | Y | Y | N | P-Y | NRSI | Y | N | N-MA | N-MA | N-MA | N | Y | N-MA | Y | LOW |
| Yeshoua 2023 | Y | Y | N | P-Y | Y | Y | N | N | N | Y | N | N-MA | N-MA | N-MA | Y | Y | N-MA | Y | LOW |
| Zare 2021 | N | N | Y | P-Y | Y | Y | N | P-Y | Y | P-Y | N | N-MA | N-MA | N-MA | Y | Y | N-MA | Y | LOW |
| Zare 2022 | N | N | Y | P-Y | Y | Y | N | P-Y | P-Y | P-Y | N | N-MA | N-MA | N-MA | Y | Y | N-MA | Y | LOW |
| Zhang 2020 | N | P-Y | N | P-Y | Y | Y | N | N | Y | Y | N | N-MA | N-MA | N-MA | Y | N | N-MA | Y | MOD |
| Zhelev 2016 | Y | P-Y | N | P-Y | Y | N | N | N | P-Y | P-Y | N | N-MA | N-MA | N-MA | Y | Y | N-MA | Y | MOD |
| **Included SRs but not extracted** | | | | | | | | | | | | | | | | | | | |
| Andrews 2012 | Y | P-Y | N | P-Y | N | Y | N | Y | Y | RCT | Y | Y | N-MA | Y | Y | Y | N | Y | MOD |
| Holstiege 2015 | Y | N | Y | P-Y | Y | Y | P-Y | Y | Y | P-Y | N | N-MA | N-MA | N-MA | Y | Y | N-MA | Y | LOW |
| Jenkins 2015 | Y | Y | Y | P-Y | Y | Y | N | P-Y | Y | Y | N | N-MA | N-MA | N-MA | Y | Y | N-MA | Y | MOD |
| Liu 2018 | Y | Y | N | Y | Y | Y | N | P-Y | NRSI | P-Y | N | N-MA | N-MA | N-MA | Y | Y | N-MA | N | LOW |
| MartinezGonzalez 2018 | Y | P-Y | Y | P-Y | Y | Y | N | P-Y | Y | RCT | N | N-MA | N-MA | N-MA | N | N | N-MA | Y | LOW |
| McDonagh 2018 | Y | P-Y | N | P-Y | N | Y | N | Y | Y | Y | N | N-MA | N-MA | N-MA | N | Y | N-MA | Y | LOW |
| Richter 2012 | Y | Y | N | Y | Y | Y | Y | P-Y | Y | RCT | N | N-MA | N-MA | N-MA | Y | Y | N-MA | Y | MOD |
| Verbakel 2019 | N | Y | N | P-Y | Y | Y | Y | Y | Y | Y | N | Y | Y | Y | Y | Y | Y | Y | MOD |
| Zhang 2022 | Y | N | Y | P-Y | N | Y | N | Y | Y | RCT | Y | Y | N-MA | Y | Y | Y | N | Y | LOW |

Notes. Yellow highlighted are items indicating critical flaws of the SR;

Abbreviations. MA: meta-analysis; N: no; N-MA: no meta-analysis conducted; NRSI: non-randomized study of the effect of interventions; P-Y: partially yes; RCT: randomized controlled trial; RoB: risk of bias; Y: yes;

# eTable 4: Mapping mentioned de-implementation strategies onto ERIC

| **Higher level categories** | **ERIC** | **Inductive codes Ingvarsson et al.** | **Inductive codes REMOVE** |
| --- | --- | --- | --- |
| **Train and educate stake-holders** | Conduct ongoing training |  |  |
|  | Provide ongoing consultation |  |  |
|  | Develop educational materials | Staff information, Provider information (by FDA), patient information |  |
|  | Make training dynamic | Staff training |  |
|  | Distribute educational materials | Guideline, Local guideline | Guideline |
|  | Use train-the-trainer strategies | Train the trainer staff trainer |  |
|  | Conduct educational meetings | Lecture (staff), Patient education |  |
|  | Conduct educational outreach visits | Academic detailing, Case based staff training, Clinical round, Consultation, Consultation (ward rounds) | Academic detailing, |
|  | Create a learning collaborative | Network |  |
|  | Shadow other experts |  |  |
|  | Work with educational institutions |  | awareness campaign led by first year medical students |
|  | *Create online learning communities* (Perry et al.) |  | online education |
| **Use evaluative and iterative strategies** | Assess for readiness and identify barriers and facilitators | Assess barriers and facilitators | Survey |
|  | Audit and provide feedback | Quality improvement contest, Goal setting and rewards, Assessment and feedback, Real time feedback, Audit and feedback, Targeted feedback (with peer comparison, benchmarking) | Cost display, incentive/team competition |
|  | Purposively reexamine the implementation |  |  |
|  | Develop and implement tools for quality monitoring | Pharmacist documentation, | Oxycodone quality improvement strategy |
|  | Develop and organize quality monitoring systems | Electronic alert system, Medication review by pharmacist, Medication review, Effectiveness feedback, Peer feedback | Prescription review, review of medical records, peer feedback, ward rounds by pharmacist, peer-to-peer review, oxycodone quality improvement strategy, |
|  | Develop a formal implementation blueprint |  |  |
|  | Conduct local needs assessment | Assess prevalence of LVC, pre-assessment of prescription practices |  |
|  | Stage implementation scale up | Feasibility |  |
|  | Obtain and use patients/ consumers and family feedback |  |  |
|  | Conduct cyclical small tests of change | PDSA |  |
|  | *ACCOUNTABILITY TOOL* (added by Ingvarsson et al.) | restrictive – compulsory order form, restrictive - expert approval and removal; - expert approval required plus review and make change, by review and make change targeted at ordering of CBC and CRP tests |  |
| **Support clinicians** | Facilitate relay of clinical data to providers | Diagnostic test | locally relevant, real-time infection epidemiological data |
|  | Remind clinicians | Clinical Decision Support (CDS), Reminders | reminders - circumstantial and physical triggered by prescribing antibiotics, reminders, decision support systems, dissemination of questionnaire about guidelines |
|  | Develop resource sharing agreements |  |  |
|  | Revise professional roles | Changes in staffing |  |
|  | Create new clinical teams | Multidisciplinary teams |  |
|  |  | *POLICY AND REGULATIONS* (added by Ingvarsson et al.) | Dissemination of antibiotic policy, restriction on purchase of antibiotics without a prescription |
| **Develop stakeholder inter-relationships** | Identify and prepare champions | Change champions |  |
|  | Organize clinician implementation team meetings | Peer support |  |
|  | Recruit, designate and train for leadership |  |  |
|  | Inform local opinion leaders |  | Local opinion leaders |
|  | Build a coalition |  |  |
|  | Obtain formal commitment | Commitment tool |  |
|  | Identify early adopters |  |  |
|  | Conduct local consensus discussions | Consensus meeting | consensus-process for guideline development, |
|  | Capture and share local knowledge |  |  |
|  | Use advisory boards and workgroups | Involve staff |  |
|  | Use an implementation advisor |  |  |
|  | Model and simulate change |  |  |
|  | Visit other sites |  |  |
|  | Involve executive boards |  |  |
|  | Develop an implementation glossary |  |  |
|  | Develop academic partnerships |  |  |
|  | Promote network weaving |  |  |
| **Change infra-structure** | Mandate change | Leadership engagement |  |
|  | Change record systems | Changes in electronic health record | Electronic medical record quantity changes |
|  | Change physical structure and equipment | Changes in test ordering system, Changes in prescription process, Facilitating of testing, Facilitating of alternative practice, Restriction in availability, adaption of equipment | rapid testing with decision support algorithm; restriction by removal and by therapeutic substitution, out-of-hours blood culture incubator, Restriction by removal, Opening a dedicated pediatric ED |
|  | Create or change credentialing and/or licensure standards |  |  |
|  | Change service sites |  |  |
|  | Change accreditation or membership requirements |  |  |
|  | Start a dissemination organization | National drug information center |  |
|  | Change liability laws |  |  |
|  | *Assess and redesign workflow* (Perry et al.) | Coordination with primary care, Patient follow up, Clinical pathway, Changes in work process | rapid reporting of microbiology results, delayed antibiotic prescribing, triage and avoidance of harmful practices,  case management and care plans, Staffing models and staff increase |
| **Utilize financial strategies** | Fund and contract for the clinical innovation |  |  |
|  | Access new founding |  |  |
|  | Place innovation on fee for service lists/formularies |  |  |
|  | Alter incentive/allowance structures | Financial incentives, Cost reduction of diagnostic tests | Financial incentives |
|  | Make billing easier |  |  |
|  | Alter patient/consumer fees | Reduced cost for patients, increased costs for patients |  |
|  | Use other payment schemes | Changes in reimbursement | Develop financial disincentives on a national level |
|  | Develop disincentives | Financial disincentives |  |
|  | Use capitated payments |  |  |
| **Adapt and tailor to context** | Tailor strategies | Tailor strategies |  |
|  | Promote adaptability |  |  |
|  | Build a coalition |  |  |
|  | Use data experts |  |  |
|  | Use data warehousing techniques | Health Information Exchange |  |
| **Provide interactive assistance** | Facilitation |  |  |
|  | Provide local technical assistance |  |  |
|  | Centralize technical assistance |  |  |
|  | Provide clinical supervision | Tailored consultation, On demand consultation | one-to-one case-based discussion |
| **Engage consumers** | Involve patients/consumers and family members |  |  |
|  | Intervene with patients/consumers to enhance uptake and adherence |  |  |
|  | Prepare patients/consumers to be active participants |  | Educational meetings with patients |
|  | Increase demand |  |  |
|  | Use mass media | Public education |  |
|  |  | *COMMUNICATION TOOL* (added by Ingvarsson et al.) | Educational material for patients to increase discussion with physicians, Education through pamphlets and counselling scripts |
| **Not categorized into higher level category** | *Engage community resources* (Perry et al.) |  |  |
|  |  | FDA BLACK BOX WARNING (added by Ingvarsson et al.) |  |
|  |  | INTERNATIONAL COLLABORATION (added by Ingvarsson et al.) |  |
|  |  |  | **Change in scope and nature of benefits and services** (daily physical activity program, using other treatments) |

# eTable 5: Description of SRs rated as critically low confidence

| **Author, Year** | **Search Period (related to database search)** | **Medical Intervention Category** | **Medical Intervention** | **Medical Specialties Category** | **Medical Specialties** | **Setting** |
| --- | --- | --- | --- | --- | --- | --- |
| **Diagnostic Imaging** | | | | | | |
| Chaudhuri 2016 | 2000 - January 15, 2015 | Diagnostic imaging | Cardiac imaging (CCT, SPECT MPI, STE, TTE) | Internal medicine | Cardiology | secondary/tertiary care inpatient/outpatient |
| Deblois 2018 | 1998 - March 28, 2017 | Diagnostic imaging | Imaging for Pulmonary Embolism | Emergency medicine | NA | tertiary care inpatient |
| Desai 2018 | 2008 - 2017 | Diagnostic imaging | C-spine imaging in adults presenting to the ED with neck trauma | Emergency medicine | NA | tertiary care (ED) inpatient |
| Edwards 2021 | inception - June, 2020 | Diagnostic imaging | Thyroid ultrasound | Other | Endocrinology, Diagnostic radiology | secondary/tertiary care inpatient/outpatient: NR |
| Goldzweig 2015 | 1995 - September, 2014 | Diagnostic imaging | Various radiologic imaging procedures (e.g., MRI, CT, pulmonary CT angiography) | Other | Radiology, Internal medicine | primary and secondary/tertiary care inpatient/outpatient |
| Goldzweig 2015 | 1995 - September, 2014 | Diagnostic imaging | Various radiologic imaging procedures (e.g., MRI, CT, pulmonary CT angiography) | Other | Radiology, Internal medicine | primary and secondary/tertiary care inpatient/outpatient |
| Winchester 2021 | 2005 - March, 2018 | Diagnostic imaging | cardiology care (e.g., echocardiograms) | Internal medicine | Cardiology | secondary/tertiary care inpatient/outpatient |
| **Drug treatments (antibiotics)** | | | | | | |
| Baysari 2016 | inception - March, 2015 | Drug treatments (antibiotics) | antimicrobial prescribing in hospitals | Other | Divers (e.g., Emergency medicine, Intensive care, Pediatrics, Family medicine, Surgery) | secondary/tertiary care inpatient |
| Bbosa 2014 | 1985 - 2010 | Drug treatments (antibiotics) | antibiotic prescribing in developing countries | Other | Any individual who handles officially and unofficially antibiotics as a way of prescribing and dispensing | primary and secondary/tertiary care  outpatient/inpatient |
| Boonacker 2010 | NR - February 26, 2009 | Drug treatments (antibiotics) | antibiotic prescribing in children with upper respiratory tract infections | Other | Pediatrics, Family medicine, Emergency medicine | Primary care, one tertiary care (ED)  outpatient |
| Carracedo-Martinez 2019 | 1996 - 2017 | Drug treatments (antibiotics) | antibiotic prescribing | Family medicine | NA | primary and secondary/tertiary care inpatient/outpatient |
| Charani 2011 | January, 1999 - April, 2011 | Drug treatments (antibiotics) | antibiotic prescribing in acute care | Other | Family medicine, Surgery, Anesthesia, | secondary/tertiary care  inpatient/outpatient: NR |
| Cox 2022 | NR - December 2021 | Drug treatments (antibiotics) | antibiotic Prescribing for Urinary Tract Infections | Family medicine | NA | primary care, one primary health clinic outpatient |
| Cuevas 2021 | 1990 - 2019 | Drug treatments (antibiotics) | antibiotic use in low- and middle-income countries | Family medicine | NA | primary and secondary/tertiary care inpatient/outpatient |
| Daniels 2021 | inception - 2020 | Drug treatments (antibiotics) | antibiotic prescribing | Other | NR | secondary/tertiary care inpatient |
| Dona 2020 | January 1, 2007 - November 21, 2018 | Drug treatments (antibiotics) | antibiotic prescribing in children aged 0-18 years | Pediatrics | NA | primary and secondary/tertiary care inpatient/outpatient |
| Hu 2016 | 1980 - December, 2015 | Drug treatments (antibiotics) | antibiotic prescribing for children with upper respiratory infections | Other | Family medicine, Internal medicine, Pediatrics | primary and secondary/tertiary care inpatient/outpatient |
| Ivanovska 2013 | 1990 - 2010 | Drug treatments (antibiotics) | Antibiotic Prescribing in Upper Middle-Income Countries | Family medicine | NA | primary care, two outpatient department  outpatient |
| Kaki 2011 | 1996 - 2010 | Drug treatments (antibiotics) | antibiotic prescribing in critical care | Critical Care | Surgery, Trauma, Neonatal, Pediatrics, medical-surgical | Tertiary care (ICU)  inpatient |
| Mahmood 2022 | 2012 - 2020 | Drug treatments (antibiotics) | inappropriate antibiotic prescribing in the Gulf region | Other | Internal medicine, Surgery, Critical Care, Oncology, Pediatrics, Emergency medicine, Hematology, Dentistry | Tertiary care  inpatient |
| Rocha 2022 | NR - February 21, 2022 | Drug treatments (antibiotics) | antibiotics prescribing in primary care | Family medicine | NA | primary care outpatient |
| Roque 2014 | January, 2001 - December, 2011 | Drug treatments (antibiotics) | antibiotics prescribing | Other | Family medicine, Pediatrics, other physicians, caregivers and pharmacy | primary and secondary/tertiary care inpatient/outpatient |
| Sadeq 2022 | January, 2010 - April, 2022 | drug treatments (antibiotics) | antibiotics prescribing in hospitals | Other | NR | secondary/tertiary care inpatient/outpatient |
| Thoolen 2012 | 1993 - 2008 | Drug treatments (antibiotics) | antibiotic prescribing in respiratory tract infections | Family medicine | NA | Primary care outpatient |
| vanderVelden 2012 | 1190 - 2009 | Drug treatments (antibiotics) | antibiotic prescribing for respiratory tract infections | Family medicine | NA | primary care inpatient/outpatient: NR |
| Wagner 2014 | 2000 - November, 2013 | Drug treatments (antibiotics) | antibiotic prescribing | Other | Various medical specialties (Intensive care, Surgery, General medical wards) | tertiary care inpatient |
| Wilkinson 2018 | 2000 - 2017 | Drug treatments (antibiotics) | antibiotic prescribing in low- and middle-income countries | Other | Divers (e.g., Family medicine, pharmacy) | primary and secondary/tertiary care inpatient/outpatient |
| Yoshikawa 2021 | NR | Drug treatments (antibiotics) | antibiotic prescribing | Family medicine | NA | primary care and secondary/tertiary care inpatient/outpatient |
| **Drug treatments (other)** | | | | | | |
| Carnes 2022 | January, 2000 - January, 2021 | Drug treatments (other) | opioid prescriptions after surgery | Surgery | Urological surgery | secondary/tertiary care outpatient |
| Diep 2018 | 1966 - June 1, 2016 | Drug treatments (other) | Intravenous immunoglobulin (IVIG) used as replacement therapy for patients with immune deficiencies | Other | Transfusion medicine | tertiary care  inpatient |
| Gould 2014 | NR - October 6, 2012 | Drug treatments (other) | benzodiazepine use in older people | Other | Geriatrics, Psychiatry | community and care homes, secondary/tertiary care inpatient/outpatient |
| Kochling 2018 | January 1, 2005 - August 31, 2016 | Drug treatments (other) | antibiotic prescriptions for acute respiratory tract infections in primary care | Family medicine | NA | primary care outpatient |
| Loganathan 2011 | 1990 - April, 2010 | Drug treatments (other) | prescribing in care homes | Other | Geriatrics, Psychiatry, Family medicine | care homes inpatient |
| Wilson 2019 | inception - January 22, 2018 | Drug treatments (other) | opioid prescribing | Other | Various Health Care Providers | NR |
| **Laboratory tests** | | | | | | |
| Bindraban 2018 | inception - July, 2016 | Laboratory tests | laboratory testing in hospitals | Other | Hospital clinicians | tertiary care inpatient/outpatient: NR |
| Cadogan 2015 | inception - February 9, 2014 | Laboratory tests | laboratory tests in primary care | Family medicine | NA | primary care outpatient |
| Engel 2012 | January, 1975 - July, 2010 | Laboratory tests | C-reactive protein measurement in adults with suspected lower respiratory tract infection | Family medicine | NA | primary care outpatient |
| Huang 2013 | inception - June, 2013 | Laboratory tests | point-of-care CRP testing and antibiotic prescribing in respiratory tract infections | Family medicine | NA | primary care outpatient |
| Rubinstein 2018 | NR - January 10, 2016 | Laboratory tests | clinical laboratory tests | Other | Family medicine, Emergency medicine | primary and secondary/tertiary care inpatient/outpatient |
| Thomas 2015 | inception - NR | Laboratory tests | Various laboratory tests | Family medicine | NA | primary care inpatient/outpatient |
| **Medical devices** | | | | | | |
| Meddings 2014 | August, 2008 - October, 2012 (Update of a previous Review with search period beginning with 1950) | Medical devices | urinary catheter use | Other | Surgery, Intensive Care, Emergency medicine, "Medical" - not defined | tertiary care inpatient |
| Murphy 2014 | inception - July, 2011 | Medical devices | urinary catheter use | Other | Emergency medicine, Surgery, General Medical Units - not further defined | tertiary care inpatient |
| **Operative treatments** | | | | | | |
| Eslambolchi 2021 | NR - June 2, 2020 | Operative treatments | cesarean sections | Obstetrics and gynecology | NA | primary care and secondary/tertiary care inpatient/outpatient |
| Opiyo 2020 | inception - June, 2019 | Operative treatments | Caesarian sections | Obstetrics and gynecology | NA | secondary/tertiary care inpatient |
| **Other** | | | | | | |
| Alostaz 2022 | inception - 2021 | Other | nonpharmacologic physical restraints in adults in ICU settings | Other | Intensive care | tertiary care (ICU) inpatient |
| Bai 2020 | 1974 - 2020 | Other | diagnostic tests (pathology tests and medical imaging) | Other | Divers (e.g., Emergency medicine, Radiology) | primary and secondary/tertiary care inpatient/outpatient |
| Colla 2017 | NR - 2015 | Other | Low value care (medication, procedures, radiology, labs/pathology, cardiac testing) | Other | Radiology, Pathology, Internal medicine, Family medicine | primary and secondary/tertiary care inpatient/outpatient |
| Damiani 2010 | NR - July 31, 2008 | Other | Fresh Frozen Plasma (FFP) transfusion | Other | Anesthesia, Physicians not further specified | Tertiary care  inpatient |
| Derzon 2019 | 1990 - 2016 | Other | transfusion for surgical and nonsurgical adult patients | Other | Surgery (orthopedic and cardiac surgery), Internal medicine, Intensive care | secondary/tertiary care  inpatient |
| Derzon 2019 | 1990 - 2016 | Other | RBC transfusion | Surgery | NA | secondary/tertiary care inpatient |
| Hiscock 2018 | January 1, 1996, and April 29, 2017 | Other | unnecessary imaging or pathology tests among pediatric patients. | Pediatrics | NA | primary and secondary/tertiary care inpatient and outpatient |
| Mohandas 2011 | 1999 - 2010 | Other | transfusion during surgery (preoperative, intraoperative, postoperative) | Surgery | NA | tertiary care  inpatient |
| **Screening** | | | | | | |
| Alber 2018 | January, 1990 - May, 2016 | Screening | cervical cancer screening | Obstetrics and gynecology | NA | primary and secondary/tertiary care inpatient/outpatient: NR |
| AlishahiTabriz 2022 | January 1, 1990 – March 4, 2021 | Screening | low value cancer care: PSA screening for average-risk men, lung cancer screening for asymptomatic patients, axillary staging and post lumpectomy radiotherapy in women older than 70 y. with clinically node-negative, hormone receptor + breast cancer | Other | Urology, Internal medicine, Gynecology Oncology | primary and secondary/tertiary care inpatient/outpatient |
| Ivlev 2018 | NR - April 6, 2018 | Screening | prostate cancer screening | Other | medical specialty: NR; partici-pants from community or GP clinic | Community, primary care  outpatient |

# eTable 6: Assessment of the confidence in the results (according to AMSTAR 2), only critically low SRs

| **Ref-ID** | **Author Year** | **1. PICO?** | **2. Protocol?** | **3. STUDY DESIGN?** | **4. SEARCH?** | **5. DUPLIC. SELECTION?** | **6. DUPL. EXTRACTION?** | **7. LIST EXCL. STUDIES?** | **8. DESCRIPTION?** | **9.a. ROB RCTs?** | **9.b. ROB NRSI?** | **10. FUNDING?** | **11.a. MA RCTs?** | **11.b. MA NRSI?** | **12. IMPACT ROB MA?** | **13. IMPACT ROB?** | **14. HETEROGENEITY?** | **15. PUBLICATION BIAS?** | **16. CONFLICT?** | **Overall confidence** |
| --- | --- | --- | --- | --- | --- | --- | --- | --- | --- | --- | --- | --- | --- | --- | --- | --- | --- | --- | --- | --- |
| 731 | Alber 2018 | N | N | N | P-Y | Y | Y | N | P-Y | NRSI | N | N | N-MA | N-MA | N-MA | N | N | N-MA | Y | CL |
| 261 | AlishahiTabriz 2022 | Y | P-Y | N | N | Y | Y | N | P-Y | Y | P-Y | N | N-MA | N-MA | N-MA | N | N | N-MA | Y | CL |
| 284 | Alostaz 2022 | Y | N | N | P-Y | Y | Y | N | P-Y | NRSI | N | N | N-MA | N-MA | N-MA | N | N | N-MA | Y | CL |
| 477 | Bai 2020 | N | N | N | P-Y | N | Y | N | N | N | N | N | N-MA | N-MA | N-MA | N | Y | N-MA | Y | CL |
| 988 | Baysari 2016 | N | Y | N | P-Y | Y | N | N | N | N | N | N | N | N | Y | Y | N | Y | Y | CL |
| 6807 | Bbosa 2014 | Y | N | N | P-Y | Y | N | N | N | N | N | N | N-MA | N-MA | N-MA | N | N | N-MA | N | CL |
| 7814 | Bindraban 2018 | N | N | N | P-Y | N | N | N | N | N | N | N | N-MA | N-MA | N-MA | N | N | N-MA | Y | CL |
| 1275 | Boonacker 2010 | Y | N | N | P-Y | Y | N | N | P-Y | Y | N | N | N-MA | N-MA | N-MA | N | N | N-MA | Y | CL |
| 8682 | Cadogan 2015 | Y | N | N | P-Y | Y | N | N | P-Y | Y | RCT | Y | Y | N-MA | N | N | N | N | N | CL |
| 253 | Carnes 2022 | Y | P-Y | N | N | Y | N | N | N | NRSI | Y | N | N-MA | Y | N | N | Y | Y | N | CL |
| 652 | Carracedo-Martinez 2019 | Y | N | N | N | Y | Y | N | Y | NRSI | N | Y | N-MA | Y | Y | Y | Y | Y | Y | CL |
| 6537 | Charani 2011 | N | N | N | N | Y | N | N | P-Y | Y | Y | N | N-MA | N-MA | N-MA | N | N | N-MA | Y | CL |
| 1026 | Chaudhuri 2016 | N | N | N | N | Y | N | N | P-Y | Y | Y | N | Y | Y | Y | Y | Y | N | Y | CL |
| 928 | Colla 2017 | N | P-Y | N | P-Y | Y | N | P-Y | N | N | N | N | N-MA | N-MA | N-MA | N | N | N-MA | Y | CL |
| 1313 | Cox 2022 | Y | N | N | P-Y | Y | Y | N | P-Y | Y | RCT | N | N-MA | N-MA | N-MA | N | Y | N-MA | Y | CL |
| 383 | Cuevas 2021 | Y | Y | Y | P-Y | Y | N | N | N | N | N | N | N-MA | N-MA | N-MA | N | Y | N-MA | Y | CL |
| 1285 | Damiani 2010 | N | N | Y | P-Y | Y | Y | N | P-Y | NRSI | N | N | N-MA | N | Y | N | Y | Y | Y | CL |
| 435 | Daniels 2021 | N | N | N | N | N | N | N | P-Y | N | N | N | N-MA | N-MA | N-MA | N | N | N-MA | Y | CL |
| 793 | Deblois 2018 | Y | Y | N | N | Y | N | P-Y | P-Y | Y | N | N | N-MA | N-MA | N-MA | N | Y | N-MA | Y | CL |
| 617 | Derzon 2019 | Y | Y | N | N | Y | Y | N | N | N | N | N | N | N | N | N | Y | N | Y | CL |
| 688 | Derzon 2019 | Y | P-Y | N | N | Y | Y | N | N | N | P-Y | N | N-MA | Y | Y | Y | N | N | Y | CL |
| 798 | Desai 2018 | Y | Y | Y | P-Y | Y | Y | N | N | N | P-Y | N | Y | N | Y | Y | Y | Y | Y | CL |
| 779 | Diep 2018 | Y | N | N | N | Y | Y | N | Y | NRSI | Y | N | N-MA | Y | N | Y | Y | N | N | CL |
| 554 | Dona 2020 | N | N | N | P-Y | Y | N | N | N | N | N | N | N-MA | N-MA | N-MA | N | N | N-MA | Y | CL |
| 348 | Edwards 2021 | N | N | N | N | Y | Y | N | P-Y | NRSI | Y | N | N-MA | N-MA | N-MA | n/a | n/a | N-MA | Y | CL |
| 1238 | Engel 2012 | Y | N | N | P-Y | Y | Y | N | N | Y | P-Y | N | N-MA | N-MA | N-MA | Y | N | N-MA | Y | CL |
| 344 | Eslambolchi 2021 | N | N | N | P-Y | Y | N | N | N | N | N | N | N-MA | N-MA | N-MA | N | N | N-MA | Y | CL |
| 30 | Goldzweig 2015 | Y | P-Y | N | N | Y | Y | N | P-Y | N | N | N | N | N | N | N | Y | Y | Y | CL |
| 1076 | Goldzweig 2015 | Y | P-Y | N | N | Y | Y | N | P-Y | N | N | N | N | N | N | N | Y | Y | Y | CL |
| 6605 | Gould 2014 | Y | N | N | P-Y | Y | Y | N | P-Y | Y | RCT | N | Y | N-MA | N | N | Y | Y | Y | CL |
| 786 | Hiscock 2018 | Y | Y | Y | N | N | Y | N | N | Y | Y | N | N-MA | N-MA | N-MA | N | Y | N-MA | Y | CL |
| 1200 | Hu 2016 | Y | Y | N | P-Y | Y | N | N | Y | Y | N | N | Y | Y | Y | Y | Y | Y | Y | CL |
| 6546 | Huang 2013 | Y | N | N | P-Y | Y | N | N | P-Y | Y | Y | N | Y | Y | N | N | N | N | Y | CL |
| 1555 | IvaNvska 2013 | N | N | N | N | N | N | N | N | N | N | N | N-MA | N-MA | N-MA | N | N | N-MA | Y | CL |
| 8345 | Ivlev 2018 | Y | Y | N | P-Y | Y | Y | Y | P-Y | Y | P-Y | N | N | N-MA | N | N | Y | Y | Y | CL |
| 8700 | Kaki 2011 | N | N | N | P-Y | Y | Y | N | P-Y | Y | Y | N | N-MA | N-MA | N-MA | Y | Y | N-MA | Y | CL |
| 766 | Kochling 2018 | Y | N | N | P-Y | Y | Y | N | P-Y | Y | RCT | N | N-MA | N-MA | N-MA | N | N | N-MA | Y | CL |
| 6514 | Loganathan 2011 | N | N | N | N | Y | N | N | N | Y | Y | N | N-MA | N-MA | N-MA | N | Y | N-MA | Y | CL |
| 291 | Mahmood 2022 | Y | N | N | N | Y | Y | N | N | N | N | N | N | N | N | N | N | Y | Y | CL |
| 1150 | Meddings 2014 | Y | N | N | P-Y | Y | Y | N | P-Y | N | N | N | N-MA | N | N | N | N | N | Y | CL |
| 1256 | Mohandas 2011 | N | N | N | N | N | N | N | N | N | N | N | N-MA | N-MA | N-MA | N | N | N-MA | Y | CL |
| 1154 | Murphy 2014 | Y | N | Y | N | N | N | N | P-Y | Y | N | N | N-MA | N-MA | N-MA | Y | N | N-MA | Y | CL |
| 480 | Opiyo 2020 | N | N | N | P-Y | Y | Y | N | P-Y | NRSI | N | N | N-MA | N-MA | N-MA | Y | Y | N-MA | Y | CL |
| 106 | Rocha 2022 | Y | P-Y | N | N | Y | N | N | P-Y | N | N | N | N-MA | N-MA | N-MA | N | N | N-MA | Y | CL |
| 1111 | Roque 2014 | Y | N | N | N | N | N | N | N | N | N | N | N-MA | N-MA | N-MA | N | N | N-MA | Y | CL |
| 773 | Rubinstein 2018 | Y | P-Y | N | N | Y | Y | N | Y | N | P-Y | Y | Y | N | Y | Y | N | N | Y | CL |
| 45 | Sadeq 2022 | Y | P-Y | N | N | Y | N | N | N | Y | Y | N | N | N | N | N | N | Y | Y | CL |
| 1052 | Thomas 2015 | N | N | N | P-Y | Y | Y | N | P-Y | NRSI | Y | N | N-MA | N-MA | N-MA | N | N | N-MA | N | CL |
| 1563 | Thoolen 2012 | N | N | N | N | N | Y | N | N | N | N | N | N | N-MA | N | N | Y | N | Y | CL |
| 6518 | vanderVelden 2012 | N | N | N | N | Y | N | N | N | N | N | N | N | N | N | N | Y | N | Y | CL |
| 6591 | Wagner 2014 | N | N | N | N | N | Y | N | P-Y | Y | Y | N |  | N-MA | N-MA | Y | Y | N-MA | Y | CL |
| 939 | Wilkinson 2018 | Y | N | N | Y | Y | Y | N | N | N | N | N | N-MA | N-MA | N-MA | N | N | N-MA | N | CL |
| 639 | Wilson 2019 | Y | N | Y | P-Y | N | Y | N | N | NRSI | N | N | N-MA | N-MA | N-MA | N | N | N-MA | N | CL |
| 446 | Winchester 2021 | Y | P-Y | N | N | Y | N | N | N | N | Y | N | Y | N | N | Y | N | Y | Y | CL |
| 335 | Yoshikawa 2021 | Y | N | N | P-Y | N | N | N | Y | Y | Y | N | N-MA | N-MA | N-MA | Y | Y | N-MA | Y | CL |

# eTable 7: Description SRs not extracted

| **Author, Year**  **Category** | **Search Period** | **Medical Intervention** | **Medical Specialties Category** | **Medical Specialties** | **Setting** | **De-implementation Strategy** | **Outcomes Reported** | **Rationale for not extracting** |
| --- | --- | --- | --- | --- | --- | --- | --- | --- |
| Verbakel 2019  Laboratory tests | inception - March, 2017 | point-of-care CRP  (C-reactive protein) tests in patients in ambulatory care | Other | Family  medicine, Emergency medicine | primary care/tertiary care (emergency department)   outpatient | point-of-care CRP tests to guide antibiotic prescribing | **Utilization/ordering of LVC:** antibiotic prescribing rate at index consultation, antibiotic prescribing during follow-up, number of additional tests performed **Health Outcomes:** referral and admission to hospital,  reconsultation, clinical recovery, time to symptom resolution **Other:** patient satisfaction | other article provides more up-to-date information (Smedemark et al., 2022) |
| Jenkins 2015  Diagnostic imaging | inception - June 23, 1014 | imaging for low back pain (e.g., lumbar spine radiography, CT or MRI) | Other | Family  medicine, Emergency medicine | primary and one secondary/tertiary care  outpatient and one inpatient | clinical decision support and targeted reminders, audit and feedback, educational material, ongoing training | **Utilization/ordering of LVC:** imaging rate, number of imaging referrals **Other:** patient satisfaction and undefined health outcomes | all articles included in other article (Belavy et al., 2022) |
| Andrews 2012  Drug treatments (antibiotics) | inception – March, 2011 | consulting or antibiotic use for acute RTIs in children (birth to 18 years) | Other | Pediatrics, Family  medicine, Emergency medicine | primary care  outpatient/ inpatient | delayed or no prescribing (watchful waiting), educational material, ongoing training | **Utilization/ordering of LVC:** antibiotic use (proportion of children consuming antibiotics) **Safety Outcomes**: Adverse effects **Other:** parental consulting rate at GP/ED, parental knowledge or attitude related to antibiotic use, **Costs:** costs of interventions | other article provides more up-to-date information (Mortazhejri et al., 2020) |
| Richter 2012  Drug treatments (other) | inception - December 19, 2011 | antipsychotic  Zmedication | Other | Geriatrics, Psychiatry | nursing and care homes  inpatient | ongoing training, educational meetings, create new clinical teams | **Utilization/ordering of LVC:** use of regularly prescribed antipsychotic medication (proportion of residents on antipsychotic medication) **Health Outcomes**: cognitive status **Safety Outcomes:** adverse effects  **Other:** prescribing of regularly psychotropic medication other than antipsychotic medication (Benzodiazepines, Antidepressants, Hypnotics, Anxiolytics) | all articles included in other article (ThompsonCoon et al., 2014) |
| Zhang 2022  Drug treatments (antibiotics) | inception – January 16, 2021 | CRP tests in adult patients (> 18 y) diagnosed with ARI | NR | NR | NR | point-of-care CRP tests | **Utilization/ordering of LVC:** antibiotic prescribing rate at index consultation, antibiotic prescribing during 28 days follow-up **Health Outcomes:** clinical  recovery of patients within 7 days **Safety Outcomes:** adverse events **Other:** patient satisfaction | other article provides more up-to-date information (Smedemark et al., 2022) |
| Liu 2018  Diagnostic imaging | inception - May, 2016 | image ordering in adult patients (≥17 y) suffering from LBP | Emergency medicine | Emergency department (ED) or other acute care setting | primary care and secondary/tertiary care (ED)  inpatient/outpatient: NR | audit and feedback, ongoing training for physicians and patients, educational material, remind clinicians, | **Appropriateness of interventions:** clinically significant injuries **Utilization/ordering of LVC:** relative change in image ordering/referrals **Other:** repeated ED presentation, heatlh service utilization | other article provides more up-to-date information (Belavy et al., 2022) |
| Holstiege 2015  Drug treatments (antibiotics) | Inception - November, 2013 | clinical decision support systems (CDSS) for improving antibiotic prescribing | Family  medicine | NA | primary care  outpatient | remind clinicians, audit and feedback, education material for patients | **Appropriateness of interventions:** Differences of proportions of visits in which all guideline metrics were achieved, adequate dosing of antibiotic treatment **Utilization/ordering of LVC:** antibiotic prescribing behaviour (reduction of antibiotic prescriptions) | all articles except one included in other article (Nabovati., 2021) |
| Martinez-Gonzalez 2018  screening | Inception - March, 2015 | Shared decision-making (SDM) for men facing prostate cancer (PC) screening decisions | Urology | NA | primary care and secondary/ tertiary care  inpatient/outpatient | educational material for patients, remind clinicians, ongoing training | **Utilization/ordering of LVC:** patient participation in PSA testing (patient-reported PSA tests ordered, physician-reported PSA tests ordered) **Other:** Knowledge (correct estimate of lifetime risk), attitude towards testing, doctor's recommendations towards PSA screening, patient-reported satisfaction, decisional conflict | other article provides more up-to-date information (Baptista et al., 2018) |

# eTable 8: Characteristics of included SRs

| **Author Y Confidence Method** | **inclusion criteria for SR (according to PICO)** | **exclusion criteria for SR** | **search period (database search)** | **Range public-cation year incl. studies** | **number of incl. studies per study design as reported** | **RoB incl. studies** | **outcomes reported** |
| --- | --- | --- | --- | --- | --- | --- | --- |
| **Drug treatments (antibiotics)** | | | | | | | |
| Coxeter 2015 MODERATE meta-analysis | **Population:** clinicians , who provide primary care or patients who present with any combination of symptoms of acute (less than four weeks' duration) respiratory infection (or the parents of similarly affected children) **Intervention:** considered if explicitly stated that the intervention was aimed at facilitating shared decision making or if the intervention explicitly addressed more than one of the essential elemenes of shared decision making described by Makoul 2006 **Outcome:** prescription of antibiotics  **Study design:** RCT (individual level or cluster) | Design: quasi-RCT, quasi-experimental studies, controlled before - after, ITS | inception - end of November/December 2014 (depending on database) | 2004 - 2013 | Overall: 10 Cluster-RCT: 10 | RoB assessed, but overall assessment NR | **Utilization/ordering of LVC:** prescription of antibiotics  **Health Outcomes:** incidence of pneumonia, incidence of hospital admissions  **Safety:** mortality due to respiratory illness  **Other:** reconsultations of patients, patient and caregiver satisfaction |
| Davey 2017 HIGH meta-analysis | **Population:** healthcare professionals who prescribe antibiotics to hospital inpatients receiving acute care **Interventions:** audit and feedback defined as any summary of clinical performance of health care over a specified period of time; education through meetings or distribution of educational materials; educational outreach through academic detailing or review of individual patients with recommendation for change; reminders provided verbally, on paper, in the workplace environment (e.g. posters or messages printed on equipment) or on computer; structural: the influence on antibiotic prescribing of changing from paper to computerised records and of the introduction of new technology for rapid microbiology testing or measurement of inflammatory markers, selective reporting of laboratory susceptibilities; formulary restriction; requiring prior authorisation (expert approval) therapeutic substitution; and automatic stop orders **Comparison:** NR **Study design:** RCTs, NRTs, CBAs, ITS | patients in nursing homes or other long-term healthcare setting; studies that compared the effectiveness of antibiotic treatments | inception - January 22, 2015 | 1976 - 2015 | Overall: 221 RCT: 58 NRCT: 8 Cohort: 8 ITS: 139 CBA: 6 Case control: 1  Qualitative: 1 | **RCT:** Moderate: 22, High risk: 36 **NRCT:** High risk: 8 **Cohort:** Low: 5, Medium: 2, High: 1 **ITS:** Low risk: 50, Moderate: 70, High risk: 19 **CBA:** High risk: 6 **Case control:** High: 1 Qualitative: not assessed (information taken from table, differs from text) | **Appropriateness of interventions:** Proportion of participants who were treated according to antibiotic prescribing guidelines, compliance with antibiotic guidelines or policies **Utilization/ordering of LVC:** duration of antibiotic treatment **Health Outcomes:** length of stay, or other clinical outcomes **Safety Outcomes:** Mortality |
| deBont 2015 LOW narrative | **Population:** patients in general practices about to receive antibiotics **Intervention:** a written information tool was studied during general practice consultations **Comparison:** standard of care / none **Study design:** RCT and NRCT | **Intervention:** dental studies, and preventative leaflets | inception - April 2014 | 1981 - 2013 | Overall: 8 RCT: 7 NRCT: 1 | RoB assessed, but overall assessment NR | **Utilization/ordering of LVC:** antibiotic prescribing, antibiotic use **Health outcomes:** reconsultation rate, severity of symptoms, duration of symptoms |
| Doan 2014 HIGH meta-analysis | **Population:** healthy children aged 0 to 18 years old **Intervention:** rapid viral diagnosis from nasal pharyngeal aspirates or swabs by direct or indirect immunofluorescent antibody test, enzyme immunoassays, optical immunoassay, or molecular testing such as multiplex polymerase chain reaction **Comparison:** no rapid viral diagnostic test performed Outcome: antimicrobial prescription rate in the ED **Studies:** RCTs | **Population:** studies considering participants who are immunocompromised, chronic severe respiratory conditions or chronic heart conditions | December 2011 - July 2014 | 2003 - 2009 | Overall: 4 RCT: 4 | **RCT:** Some concerns: 1, High risk: 3 | **Utilization/ordering of LVC:** Antimicrobial prescription rate in the Emergency Department, Rate of ancillary tests (any blood tests, urine investigations or chest radiography) requested **Health Outcomes**: Rate of physician visit within two weeks after discharge from ED, Hospital admission rate **De-Implementation Outcome:** Acceptability of nasal specimen collection sampling for rapid viral testing |
| Fleming 2013 LOW narrative | **Population:** patients with UTI, RTI and skin and soft tissue infetction types in long-term care facilities (LTCF) **Intervention:** interventions aimed at reducing antibiotic prescribing in above mentioned patients, including educational interventions, audit and feedback, provision of written materials or guidelines, medication review services and case-conferencing **Comparison:** usual care or other interventions **Study design:** RCTs and cluster RCTs | **Intervention:** financial, regulatory and information and communication technology nature **Outcome:** reduction of medication errors | inception - August 2012 | 2001 - 2011 | Overall: 4 RCT and cRCT: 4 | **RCT and cRCT:** High risk: 2, Unclear risk: 2 | **Utilization/ordering of LVC:** antibiotic use at time of diagnosis, total antimicrobal use  **Safety Outcomes:** antibiotic resistances, mortality rate |
| Lane 2018 LOW narrative | **Population:** primary care clinicians management of common (respiratory, gastrointestinal, urinary and skin) infections in OECD member countries **Intervention:** including dissemination of real-time, population-based data on locally relevant microbes or syndromic presentations | **Intervention:** surveillance systems for conditions such as human immunodeficiency virus (HIV), tuberculosis and malaria were excluded | inception - April 2016 | 1999 - 2014 | Overall: 3 cRCT: 1 Cohort study: 2 | **cRCT:** Serious risk: 1 **Cohort study:** Serious risk: 1, High risk: 1 | **Utilization/ordering of LVC:** mean antibiotic prescribing rate, antibiotic prescribing rate, likelihood of antibiotic prescribing |
| Lim 2020 LOW narrative | **Population**: community **Intervention**: national- level and/or subnational-level responsible use initiatives **Comparison**: Before-After **Study design:** Cohort, time series, quasi-experimental | description of implementation without outcomes reported, antimicrobials other than antibiotics, intervention not related to antibiotic use (e.g. surveillance), animal studies | inception - May 2017 | 1999-2017 | Overall: 34 NRCT: 1 Prosp. Cohort: 30 Restrosp. Cohort: 3 | low risk: 6, moderate risk: 20, high risk: 8 | **Appropriateness of interventions:** inappropriate access **Utilization/ordering of LVC:** reduction in demand, reduction in antibiotic use |
| Martinez-Gonzalez 2020 HIGH meta-analysis | Population: patients with respiratory tract infections in primary care Intervention: Point-of-Care C-Reactive Protein Testing Comparison: standard care Study Design: RCT | excluded studies on patients with obstructive pulmunory disease and exacerbations and/or other pre-existing chronic pulmonary diseases, as well as RCTs from in-patient (e.g., hospitalised) settings | inception to May, 2019 | 1995-2018 | Overall: 13 RCT: 13 studies in 22 publications | RoB assessed, but overall assessment NR | **Utilization/ordering of LVC:** prescribing rate at index consultation **Health Outcomes:** Clinical Recovery and Resolution of Symptoms, Re-Consultations and Intention to Re-Consult, referrals to secondary care, Ordering of Investigations **Safety Outcomes:** Admissions to Hospital and Mortality **De-implementation Outcomes:** Visit Duration and Visits at Follow-Up  **Other:** patient satisfaction and enablement |
| Mortazhejri 2020 HIGH meta-analysis and narrative | **Population:** patients of all age groups with upper respiratory tract infections (URTI), seeking treatment in general practice setting  **Intervention**: any intervention directed to patients to reduce unnecessary antibiotics for URTIs **Comparison:** primary outcomes, precription for use of antibiotics for URTIs; secondary outcomes, satisfaction eith treatment/consultation, beliefs that antibiotics are effective for URTIs, re-consultation for the same illness in the next 2 weeks  **Study design:** RCT, quasi-RCT, CBA, ITS | **Population:** patients with lower respiratory tract infections (LRTI) and those with chronic obstructive lung conditions (COPD) **Intervention:** interventions directed to healthcare providers or clinical staff , interventions that target patients indirectly, patient decisions aids **Comparison:** other comparisons  Outcomes: studies that do not report the primary outcome | inception - October 23 - November 11, 2016 | 1997 - 2016 | Overall: 13 cRCT and RCT: 12 ITS: 1 | RoB assessed, but overall assessment NR | **Utilization/ordering of LVC:** reduction of antibiotic prescriptions, use of antibiotics, collection or filling of prescriptions **Health Outcomes:** re-consultation rates **Safety Outcomes:** mortality, adverse effects, antibiotic resistances **Costs:** economic burden due to antibiotic resistance  **Other:** patients satisfaction, patients' beliefs on the effectiveness of antibiotics to treat URTIs |
| Nabovati 2021 LOW narrative | **Population**: patients with respiratory tract infections **Intervention**: information technology (IT) **Comparison**: standard care **Study Design**: (cluster-)RCT | (a) studies conducted on diseases other than ARI, (b) studies using interventions other than IT for improving antibiotic prescribing, (c) studies which focused on the prescription of medications other than antibiotics, (d) studies which did not examine the outcomes related to antibiotic pre- scribing in intervention and control groups, and (e) review articles, letters to editors, protocols, conference papers, and theses/ dissertation | inception - 31 August 2020 | 2001-2020 | Overall: 18 (Cluster)-RCT: 18 | **(Cluster)-RCT:** Strong: 2, Moderate: 11, Weak: 5 | **Appropriateness of interventions:** adherence to guideline metrics **Utilization/ordering of LVC:** rate of antibiotic prescription, Reduced duration of therapy below the 10-day **Health Outcomes:** Revisits **Safety:** Serious bacterial complications, Comorbidity status of patients consulting with RTI, Strategy failure |
| Nair 2021 MODERATE narrative | **Population:** Healthcare providers (doctors, informal doctors, nurses, pharmacists, CHWs) in LMICs who prescribe or provide antibiotics in primary care, outpatient or community-based settings **Interventions:** bevavioural interventions such as persuasive (prescription audits and feedback advice), enabling (education or guidelines on anitbiotic use), restrictive (expert appoval prior to using certain antibiotics), structural (introduction of a new diagnostic test or clinical algorithm tu guide prescriptions) or bundle (mix of different interventions) **Outcome:** knowledge, attitudes and practices regarding antibiotic use following an intervention among prescribers **Study design:** RCTs, NRCT, ITS, UBA, qualitative studies **Setting:** outpatient or ambulatory care | **Population:** studies focusing on patients with malaria, HIV, malnutrition or other infectious diseases **Interventions:** interventions focusing on microbiological tests, or improving infection prevention and control **Setting:** hospital inpatients and nursing homes | 2001 - 2019 | 2002 - 2019 | Overall: 13 cRCT and RCT: 7  Controlled-non-inferiority trial: 1 UBA: 5 | RoB assessed, but overall assessment NR | **Appropriateness of interventions:** (number of) appropriate or inappropriate antibiotic prescription, antibiotic prescription rates **Safety Outcomes:** proportion of children checked for danger signs **Costs:** costs **Other**: Difference in improvement of knowledge/practice |
| Nguyen 2019 MODERATE narrative | **Population**: residents in care homes aged >65, Healthcare staff working in care homes or associated with care homes **Intervention**: any interventions that aimed to enhance antimicrobial stewardship **Comparison**: Usual care or other interventions **Study Design:** RCT or cRCT | non english publications | inception - November 30, 2018 | 2001-2014 | Overall: 5 cluster-RCT: 5 | RoB assessed, but overall assessment NR | **Appropriateness of interventions:** guideline adherence **Utilization/ordering of LVC:** prescription rate, use of antimicrobiotics **Health Outcomes:** severity of pneumonia **Safety Outcomes:** hospitalizations, 30-day mortality, admission to hospital **Other:** urinary tests ordered |
| O'Sullivan 2016 HIGH narrative | **Population:** Patients presenting acute URTI **Intervention:** Written information for patients including details about antibiotics for acute URTIs **Comparison:** no information for patients **Study design:** RCTs | **Intervention:** excluded studies that offered information after prescribing, for example, pharmacists providing education via package inserts, excluded interventions where only verbal information was given. | inception - July 6, 2016 | 2000 - 2009 | Overall:  RCT: 2 | Moderate risk: 1, High risk: 1 | **Utilization/ordering of LVC**: number of antibiotics used by patients, number of antibiotics prescribed **Health Outcomes:** reconsultation rate, resolution of symptoms **Savety Outcomes:** complications **Other:** knowledge, patient satisfaction |
| Raban 2023 LOW narrative | **Population:** Patients treated in primary care facilities, general and family practices **Intervention:** nudge interventions (i.e. any aspect of the choice architecture that alters people’s behaviour in a predictable way without forbidding any options or significantly changing their economic incentives); intervention must be easy and cheap to avoid **Study design:** RCTs, regression discontinuity studies | **Intervention:** education, providing physicians with access to guidelines, passive decision support tools the clinician had to actively decide to use, and audit and feedback interventions with no social norm comparison; multifaceted interventions that included a nudge strategy **Study design:** ITS, CBA, UBA | 1997 - April 23 2021 | 2006 - 2021 | Overall: 19 RCT: 17 regression discontinuity studies: 2 | **RCT:** low risk: 6, moderate risk: 7, high risk: 4 R**egression discontinuity studies:** moderate risk: 2 | **Utilization/ordering of LVC:** rates of antibiotic prescribing overall |
| Rajar 2020 MODERATE narrative | **Population**: infants born <34 weeks or <1500g birth weight **Intervention**: Antibiotic stewardship **Comparison**: before-after **Study Design**: Before-After | where antibiotic stewardship actions were directed toward specific microorganisms; and studies only reporting on antibiotic use but no actions | not specified - December 9, 2019 | 2010-2019 | Overall: 12 RCT: 1 Prosp. Cohort: 3 Restrosp. Cohort: 8 | **RCT:** 3 out of 5 points: 1 **cohort studies:** good quality: 11 | **Appropriateness of interventions:** innapropriate antibiotic days  **Utilization/ordering of LVC:** daily defined dose, days of therapy **Safety Outcomes:** mortality, sepsis incidence |
| Siachalinga 2022 LOW narrative | Population: hospital inpatients Intervention: Antimicrobial stewardship interventions (strategy, policy, guideline or tool aimed at improving antibiotic prescribing)  Study design: NR | **Population:** outpatients and communities **Study design:** descriptive studies, conference abstracts, reviews and protocols, antimicrobial resistance studies | January 1, 2010 - July 4, 2022 | 2011 - 2022 | Overall: 28 cRCT: 2 ITS: 1 UBA: 25 | **cRCT:** High risk: 2 **ITS:** High risk: 1 **UBA:** Good: 23, Fair: 2 | **Appropriateness of interventions:** rate of compliance with target practice **Utilization/ordering of LVC:** usage of antibiotics **Health outcomes:** length of hospital stay **Safety Outcomes:** healthcare-associated infections, mortality |
| Smedemark 2022 MODERATE meta-analysis | Population: patients of all ages with symptoms or diagnosis of an acute respiratory infection (ARI) in primary care Intervention: Point-of-Care tests (C-Reactive Protein, procalcitonin, white blood cell count) for infection biomarkers to guide antibiotic treatment  Comparison: standard care Outcome: Rate of antibiotics prescribed Study Design: RCT and cluster RCT's | specific diagnostic tests like the Strep A test or Monospot, electronic algorithm in combination with a specific point-of-care test, antigen testing, clinical decision support to guide antibiotic decisions | inception - June, 15, 2022 | 1995-2021 | Overall: 13 cRCT and RCT: 13 | RoB assessed, but overall assessment NR | **Utilization/ordering of LVC:** number of patients with antibiotic prescription **Health Outcomes:** number of patients with substantial improvement, 7 day recovery, reconsultation, duration of infection, recovery rate **Safety Outcomes:** total mortality, hospitalisations  **Other:** patient satisfaction |
| Spurling 2017 HIGH meta-analysis | **Population:** adults and children diagnosed with RTIs **Intervention:** delayed antibiotic use (i.e. advice to delay the use of antibiotics afte an anbiotic prescription by at least 48 hours after initial consultation) **Comparison:** immediate antibiotic use (given at initial consultation) or no antibiotics, **Study design:** randomized controlled trials | NR | inception - May 25, 2017 | 1987-2016 | Overall: 11 RCT: 11 | **RCT:** Low: 11 | **Utilization/ordering of LVC:** reduction of antibiotic use **Health Outcomes:** clinical outcomes for sore throat, acute otitis media, bronchitis and common cold **Safety Outcomes:** complications related to diseases mentioned above, adverse effects of antibiotics, antibiotic resistance, complication of disease  **Other:** patient satisfaction, reconsultation rates, use of other therapies |
| VanDijck 2018 MODERATE narrative | **Population:** hospitalized patients in low and middle-income countries (World Bank criteria) **Intervention:** antibiotic stewardship interventions (any intervention aiming to improve appropriate prescribing of an antibiotic) **Study design:** RCT, NRCT, ITS, CBA | **Population:** residents of long term healthcare or nursing facilities, malaria, human immunodeficiency virus, mycobacterial or fungal infections, Helicobacter pylori eradication, care pathways **Language:** other than English, Dutch, French, German, Portuguese, Spanish | inception - December 15, 2017 | 2001 - 2017 | Overall: 27 (Cluster)-RCT: 12 NRCT: 3 ITS: 12 | **(Cluster)- RCT:** Medium risk: 3, High risk: 9 **NRCT:** High risk: 3 **ITS:** Low risk: 3, Medium risk: 8, High risk: 1 | **Utilization of ordering LVC:** receiving antibiotics; % of operations with antibiotic prophylaxis; mean difference in treatment duration; difference in median no. of days of treatment; DDD per 1000 bed-days; % of caesarean sections with administration of antibiotic phrophylaxis within 1 hour of delivery;  **Appropriateness of interventions:** appropriate prescribing of antibiotics; % of operations with incorrect timing of antibiotic prophylaxis **Safety Outcomes:** in-hospital death **Other:** mean difference in hospital length of stay |
| Vodicka 2013 LOW narrative | **Population:** children (<18 years) with RTIs **Intervention:** primary care based intervention (educational and/or behavioural) to reduce antibiotic prescribin for children with RTIs **Comparison:** no-treatment or alternate treatment controls **Study design:** Controlled studies (randomised, cluster randomised, non-randomised or one-group pre- and post-test design) | inpatient-settings, evaluations of treatment guidelines, public health interventions, diagnostic tests; studies of children with chronic illnesses or serious comorbidities; or studies from countries not classified as high-income by the Organisation for  Economic Co-operation and Development | inception - June 2012 | 1992 - 2011 | Overall: 17 RCT: 12 NRCT: 2 UBA: 3 (one-group pre- and post-test design) | Minimum risk: 1, Low risk: 2, Moderate risk: 13, High risk: 1 (not individually reported for study designs) | **Appropriateness of interventions:** change  in ‘appropriate’ antibiotic prescribing **Utilization/ordering of LVC:** change in proportion of antibiotic prescriptions  issued for RTIs in children |
| **Drug treatments (opioids)** | | | | | | | |
| Badreldin 2023 LOW narrative | **Population**: postpartum patients less than 8 weeks following birth **Intervention:** including the use of lidocaine patches, postoperative abdominal binder, valdecoxib, and acupuncture only following birth  **Study design:** RCT, NRCT, Retrospective cohort, Implementation studies | **Interventions:** initiated during the antepartum and intrapartum period | inception - September 01, 2021 | 2006 - 2021 | Overall: 24 RCT: 8 UBA: 16 | **RCT:** Good: 3, Fair: 4, Poor: 1 **UBA:** Good: 5, Fair: 11 | **Utilization/ordering of LVC:** number of opioid/oxycodone tablets used on postoperative day, reduction in morphine equivalents |
| Daoust 2022 MODERATE meta-analysis and narrative | **Population:** adults ≥ 18 years old discharged from the emergency departement  **Intervention:** all interventions designed to reduce the opioid prescription rate and/or the quantity of opioids per pescription **Study design:** all study designs except the ones mentioned under exclusion criteria | **Population:** pediatric patients, patients with a substance use disorder  **Intervention:** opioids given during emergency departement stay, opioid use unrelated to pain  **Setting:** other than ED **Design:** not reported opioid prescribing rate or quantity | inception - March 6, 2021 | 2013 - 2021 | Overall: 63 RCT: 1 Restrosp. Cohort: 2 ITS: 21 UBA: 39 | **RCT:** Low risk: 1  **Retrosp. Cohort:** High risk: 2  **ITS:** Moderate risk: 10, Serious or critical risk: 11 **UBA:** Serious or critical risk: 39 | **Utilization/ordering of LVC:** prescription rate, prescribed opiod quantities  **Health Outcomes:** patients' level of pain relief, patients' need for additional opiod prescriptions  **Safety Outcomes:** long term dependence, opioid disorders, risk of overdose  **Other:** patient satisfaction with opiod prescriptions |
| Phinn 2023 MODERATE narrative | **Population:** admitted patients being discharged from hospital inpatient care or the ED **Intervention:** organizational interventions targeting the appropriate prewcribing of opioids for noncaner pain upon hospital discharge | **Population:** patients < 18 years old, patients in palliative care patients with oncology/cancer pain, opioid-substitution therapy **Interventions:** interventions primarily involving state laws and mandates **Study design**: case reports, case series, conference abstracts, expert opinion articles, literature reviews  **Language:** other than English | 2011 - March 23, 2021 | 2013 - 2021 | Overall: 43 RCT and cluster RCT: 3 Prosp. Cohort: 1 Restrosp. Cohort: 3 ITS: 2 UBA: 33 Case-control: 1 | **RCT:** Low risk: 2, Some concerns: 1 **Prosp. Cohort:** Serious risk: 1 **Retrosp. Cohort:** Serious risk: 3 **ITS:** Low risk: 1, Serious risk: 1 **UBA:** Low risk: 7, Moderate risk: 4, Serious risk: 21, Critical: 1 **Case-control:** Serious risk: 1 | **Utilization/ordering of LVC:** reduction of opioid prescriptions  **Health Outcomes:** patients self-reported pain levels, postoperative phone calls for uncontrolled pain **Safety Outcomes:** number of patients with return visits to ED **Other:** patient satisfaction with pain control after discharge |
| Zhang 2020 MODERATE narrative | **Population:** adults (≥ 18 years) undergoing any type of surgery **Intervention:** behavioural interventions implemented within a healthcare institution to decrease opioid prescribing at discharge after surgery **Study design:** RCT, NRCT, pre-post studies, ITS, cohort studies, case-control studies, historically controlled studies, cross-sectional studies | **Population:** pediatric patients  **Intervention:** effect of specific surgical techniques or perioperative anesthetic interventions on postoperative opioid consumption; evaluation on the effect of policy-level interventions | inception - December 14, 2018 | 2013 - 2019 | Overall: 24 RCT: 1 Cohort: 2 ITS: 2 UBA: 19 | **RCT:** High risk: 1  **Cohort:** 6/9 stars: 2  **ITS:** Medium risk: 2 **UBA:** Medium risk: 12, High risk: 7 | **Utilization/ordering of LVC:** number of opioid/oxycodone tablets, morphine equivalents; patients discharged with opioids **Health outcomes:** pain control **Safety Outcomes:** unintended harms through unsafe storage or disposal, opioid dependence |
| **Drug treatments (antipsychotics, benzodiazepines)** | | | | | | | |
| Birkenhäger-Gillesse 2018 LOW meta-analysis | **Population:** individuals diagnosed with dementia living in long-term care facilities **Intervention:** Psychosocial interventions directed at staff or the environment **Comparison:** usual care **Study design:** RCTs | **Intervention:** interventions that solely focused on the prescription behavior of the physician **Study design:** solely focusing on the prescription behaviour | NR - June 21, 2017 | 1992 -2016 | Overall: 11 RCT: 11 | **RCT:** strong: 8, low to moderate: 3 | **Utilization/ordering of LVC:** proportion of participants using psychotropic medication, such as antipsychotics, antidepressants, anxiolytics, and/or hypnotics including benzodiazepines |
| Mokhar 2018 LOW narrative | **Population:** middle-aged adult (45 years and older) users of BZD or z-drugs and/or health care professionals (HCPs) involved in the care process **Intervention:** promote patient-centered treatments for inappropriate BZD and z-drug use **Study design**: any study design with a control | **Population:** children or on chronically or seriously mentally ill patients; if the use of BZDs was indicated (e.g., for severe psychiatric disorders such as schizophrenia) **Study design:** case series, review papers, meta-analyses, double publications, experimental research, protocols and animal research | NR - October 2014 | 1992 - 2014 | Overall: 20 RCT: 14 NRCT: 6 | RoB assessed, but overall assessment NR | **Utilization/ordering of LVC:** reduction in BZD use |
| ThompsonCoon 2014 MODERATE narrative | Population: individuals with dementia  intervention: aimed to reduce inappropriate prescription of antipsychotic medications   setting: community residential care settings | NR | inception - November 2013 | 1987 - 2013 | Overall: 22 RCT: 6 CCT: 5 UBA: 11 | **RCT:** strong: 2, moderate: 2, weak: 2 **CCT:** strong: 1, weak: 4 **UBA:** moderate: 3, weak: 8 | **Utilization/ordering of LVC:** medication use |
| **Laboratory tests** | | | | | | | |
| Dunn 2021 LOW narrative | **Population**: adult patients with CDI (Clostridioides difficile infection), physicians ordering tests for CDI diagnostics  **Intervention**: Clinical Decision Support Alerts for  CDI diagnosis and CDI testing volume | Review articles, editorials, conference abstracts, studies evaluating clinical decision tools related to CDI treatment and studies conducted exclusively in pediatric units, as diagnostic and management  strategies differ between older children/adults and children < 3 years old | inception - May, 13th 2019 | 2013 - 2019 | Overall: 11, unclear  study designs | Moderate risk: 4, Serious risk: 7 | **Appropriateness of interventions:** number of appropriate tests **Utilization/ordering of LVC:** number of tests ordered **Health Outcomes:** CDI rate |
| Kobewka 2015 MODERATE narrative | **Population:** physicians  **Intervention:** modification of test utilization (educational, audit and feedback, system based, incentive and penalty interventions aimed at reducing test utilization) **Comparison:** standard care or no intervention **Study design:** all | NR | inception - September , 2013 | 1974 - 2013 | Overall: 109 RCT: 14 NRCT: 2 UBA: 93 | RoB assessed, but overall assessment NR, EPOC guidlines used | **Utilization/ordering of LVC:** relative reduction in laboratory test utilization  **Costs:** costs |
| Yeshoua 2023 LOW narrative | **Population:** adult patients in inpatient departments **Intervention:** interventions aimed to reduce daily CBCs, comprehensive metabolic panel and BMPs (basic metabolic panel) **Comparison:** NR **Study design:** all studies meeting inclusion criteria | population: paediatric and intensive care unit; intervention: other tests such as coagulation or liver function tests, as per Choosing Wisely recommendations; Cross-sectional studies without interventions | inception - May 06, 2021 | 1981 - 2021 | Overall: 41 RCT: 2 NRCT: 4 Prosp. Cohort: 35 | **RCT:** good: 2 **NRCT:** good: 4 C**ohort:** good: 35 | **Utilization/ordering of LVC:** ordering daily complete blood count, complete metabolic panel and basic metabolic panel labs |
| Zare 2021 LOW narrative | **Population**: **laboratory tests, physicians ordering laboratory tests, or patients for whom laboratory tests were ordered** **Intervention**: clinical decision support systems (CDSS; health information technology system designed to provide assistance to physicians at the time of decision making) to improve laboratory test ordering as the primary aim **Comparison:** NR **Study design**: RCTs, non-randomized controlled clinical trials, prospective observational studies, before-after, ITS | published in any languages rather than English, conducted in outpatient or primary care settings, used as interventions rather than CDSS, conducted in an unreal clinical environment or based on a scenario, retrospective studies | inception - January 21, 2020 | 1995 - 2018 | Overall: 16 RCT: 1 ITS: 8 UBA: 7 | **RCT:** good: 1 **ITS:** good: 2, fair: 6 **UBA:** good: 2, fair: 5 | **Appropriateness of interventions:** diagnostic yield and diagnostic detection rate **Utilization/ordering of LVC:** number of ordered tests proportion of tests, test interval, number of STAT request **Safety Outcomes:** patient complication, mortality rate, length of stay **De-implementation Outcomes:** guideline adherence, orders cancellation after the reminders **Costs:** cost of tests **Other:** physicians knowledge and attitude |
| Zhelev 2016 MODERATE narrative | **Population:** NR **Intervention:** targeted to reduce the number of inappropriately ordered TFT (thyroid function tests)  **Study design:** RCTs, NRCTs, UBA | studies that targeted TFTs along with other tests and only reported the average effect | NR - May 1, 2015 | 1979 - 2014 | Overall: 27 RCT: 4 NRCT: 5  ITS: 2 UBA: 16 | **RCT:** Strong quality: 4 **NRCT:** Moderate quality: 2, Weak quality: 3 **ITS:** Strong quality: 1, Moderate quality: 1 **UBA:** Strong quality: 1, Moderate quality: 3, Weak quality: 12 | **Utilization/ordering of LVC:** change in the total number or rate of TFTs **Appropriateness of interventions:** variation of TFT ordering, rate of inappropriate test ordered  **Other:** test-related expenditure or health benefits to individual patients (eg, the number of unnecessary tests or treatments avoided) |
| **Diagnostic imaging** | | | | | | | |
| Belavy 2022 MODERATE meta-analysis | **Population:** adults (>=18 years) with low back pain with or without leg pain (without specific spinal pathologies) **Intervention:** professional, financial, organisational, patient-oriented, structural, or regulatory interventions to improve imaging referrals **Comparison:** no intervention control group, standard practice control group and/or untargeted activity **Study design:** Randomised controlled/clinical trials | persons < 18 years, patients with specific spinal pathologys (i.e., vertebral fracture, malignancy, …), Studies that were prospective interventional trials but not randomised were not included in the main analysis but for extraction to address a secondary goal. | inception - June 14, 2021 | 2000 - 2021 | Overall: 8  Cluster-RCT: 8 | **Cluster-RCT:** Low risk: 6, Some concerns: 2 | **Utilization/ordering of LVC:** proportion or percentage of imaging referrals or imaging usage, percentage change in imaging referrals |
| Dunne 2022 MODERATE narrative | **Population:** adult and pediatric ED patients undergoing diagnostic CT **Intervention:** ED-based intervention to reduce CT utilization **Comparison:** concurrent or historic groups receiving no intervention **Study design:** randomized or nonrandomized intervention studies | **Population:** patients from other departments | inception - December 31, 2020 | 1997-2021 | Overall: 149 RCT: 14 NRCT: 7 UBA: 127 NR: 1 | **RCT:** Low rik: 3, Some concerns: 9, High rik: 2 **NRSI and UBA:** Moderate risk: 34, Serious risk: 73, Critical: 28 | **Utilization/ordering of LVC:** Reduction in CT Scan Usage **Safety Outcomes:** ED revisit or readmission, missed or delayed diagnosis, complications, adverse events |
| Kjelle 2021 LOW narrative | **Intervention:** interventions to reduce the use of low-value diagnostic imaging examinations **Study design:** empirical study | studies on animals, mass screening, and unnecessary care; published before 2010 or after 2020; dental imaging, optical imaging, thermal imaging, microscopic imaging; study-design: SR & MA, patient case reports, letter, comment | 2010 - October 13th, 2020 | 2010-2020 | Overall: 95 RCT: 9 NRCT: 2 Prosp. Cohort: 3 Restrosp. Cohort: 5  ITS: 5 UBA: 14 Other: 57 | Low Risk: 46 (full score MMAT appraisal), Some concerns: 49 (one criterion unfullfilled) | **Utilization/ordering of LVC:** number or rate of imaging exmination |
| Zare 2022 LOW meta-analysis and narrative | **Population**: imaging procedures of CNS, physicians ordering CNS imaging, or the patients for whom CNS imaging procedures were ordered in any clinical setting, including primary, emergency, and specialist care **Intervention**: CDSS interventions to improve appropriate image ordering for CNS injuries (Any electronic decision rule provided to physicians either standalone or integrated into electronic health record (EHR) or computerized physician order entry (CPOE) was considered as a CDSS) **Comparison**: NR **Study type**: : Experimental and quasiexperimental study designs (randomized controlled trials (RCT), quasiexperimental, nonrandomized controlled clinical trials (NRCT), prospective observational studies, cohort, and interrupted time series (ITS)) | (1) published in any language rather than English; (2) examined feasibility, validity, accuracy, and usability; (3) described a CDSS; (4) used interventions rather than computerized CDSS; (5) conducted based on a scenario or in an unreal clinical environment, (6) descriptive studies; and/or (7) presented as a congress abstract.) | inception - August 11, 2020 | 2006 - 2020 | Overall: 11  NRCT: 7 Cohort: 1  ITS: 1 UBA: 2 | Moderate quality: 4, Poor quality: 7 | **Appropriateness of interventions:** diagnostic yield and diagnostic detection rate, guideline adherence for imaging ordering  **Utilization/ordering of LVC:** proportion and number of imaging **Health Outcomes:** patients length of stay (LOS)  **Safety Outcomes:** patients’ complications or undetected fractures, readmission, patients’ disposition, and mortality rate  **Costs:** cost of imaging **De-Implementation Outcomes:** adherence or order cancellation after the reminders of CDSS (or overriding the reminders) **Other:** physicians’ knowledge and attitude |
| **Other (imaging, laboratory tests, physiological tests)** | | | | | | | |
| Foster 2020 LOW narrative | **Population:** healthcare professionals ordering laboratory tests or blood transfusion components for patients in an intensive care unit **Interventions:** audit and feedback intervention  **Comparison:** usual care (no intervention: historical or condurrent), or any other single or multifaceted behavioural intervention that did not involve audit and feedback **Outcome:** number of laboratory tests ordered Study design: RCT, NRCT, CBA, UBA, ITS | studies not reporting intensive care unit specific data were excluded | inception - October 28th, 2016 | 1988-2016 | Overall: 16 studies (17 publications) RCT: 1 NRCT: 1 UBA: 13 CBA: 1 | RoB assessed, but overall assessment NR | **Appropriateness of interventions:** total number/proportion of inappropriate tests before/after the intervention; total number/proportion of inappropriate transfusion before/after the intervention  **Utilization/ordering of LVC:** total number/proportion of tests before/after the intervention; total number/proportion of transfusion before/after the intervention  **Health Outcomes:** length of stay  **Safety Outcomes:** mortality **Costs:** estimated total gross direct cost savings; estimated net cost savings |
| Takada 2020 LOW narrative | **Population:** patients in primary care **Intervention:** low-value medical tests **Outcome:** usage of low-value medical tests **Study design:** RCT **Language:** English, German, French or Dutch | NR | 1990 - Nov 12th, 2019 | 1990-2017 | Overall: 16 RCT: 16 | **RCT:** High risk: 12, Low risk: 4 | **Appropriateness of interventions:** total number/proportion of low-value tests before/after the intervention **Utilization/ordering of LVC:** total number/proportion of tests before/after the intervention **Health Outcomes:** patient-reported outcomes measures **Safety Outcomes:** consequences of healthcare use (delay of diagnosis, treatment or referaral, frequency of complication/death), revisit to primary care/emergency room, frequency of unexpected admission  **Costs:** costs for medical tests **Other:** use of other tests |
| Xie 2022 MODERATE narrative | **Population:** clinicians or patients, any health condition **Interventions:** clinical dashboard interventions used as Clinical Decision Support Systems (CDSS) or A&F, clinical dashboards providing a visual summary of deciscion-related information displayed in graphs, charts or interactive tables **Control:** any type considered  **Outcome:** medication use, including the rate of medication prescribed/administered and medication intake adherence; test ordering, imaging referrals and the count of routine laboratory test orders **Setting:** any primary care or hospital setting **Design:** RCTs | **Population:** healthy populations or healthcare students | inception - August 2021 | 2010 - 2021 | Overall: 11 RCT: 11 | **RCT:** Low risk: 5, Some concerns: 4, High risk: 2 | **Utilization/ordering of LVC:** medication prescription, unnecessary referrals for diagnostic imaging or laboratory tests  **Other:** medication adherence |
| **Other interventions** | | | | | | | |
| Baptista 2018 LOW meta-analysis | **Population:** men who had not been previously diagnosed with prostate cancer and considering prostate cancer screening decision **Intervention:** web-based decision aids (corresponding to any program accessed over a network connection using HTTP or through a Web-based app) **Comparison**: no intervention/usual care; alternative decision aids formats  **Outcome:** at least one quality of decision-making outcome (e.g. knowledge, decisional conflict, and involvement in decision making, actual screening behavior) **Study design:** RCT | For interventions to be considered Web-based, materials such as CD-ROMs or DVDs, although computer-based, were not considered Web-based and studies with such interventions were excluded | NR - November 2016 | 2003 - 2013 | Overall: 7 RCT: 7 | RoB assessed, but overall assessment NR | **Utilization/ordering of LVC:** actual screening behaviour (choice of PSA screening) **Other**: Knowledge, Decisional Conflict, Participation in decision making |
| Chen 2018 HIGH narrative | **Population:** Pregnant women seeking maternity care during pregnancy, labour and delivery; families of pregnant women, healthcare providers who work with pregnant women (nurses, midwives, physicians); healthcare facilities that provide maternity care to pregnant women **Intervention:** targeted at women, the community or the general public (e.g. birth preparation classes); targeted at healthcare professionals (e.g. implementation of clinical practice guidelines); targeted at healthcare organisations or facilities (e.g. different payment systems for caesarean section) **Comparison:** no intervention, usual care or practice in accordance with local protocols or another intervention, as reported in the studies **Study design:** RCT, NRCT, CBA, ITS | NR | inception - March 7, 2018 | 1991 - 2016 | Overall: 29 RCT: 19 CBA: 1 ITS: 9 | **RCT:** assessed, but not classified: 19 **CBA:** assessed, but not classified: 1 **ITS:** assessed, but not classified: 9 | **Utilization/ordering of LVC:** Caesarean section rate emergency, caesarean section rate elective, spontaneous vaginal birth, instrumental vaginal birth **Health Outcomes:** maternal mortality and morbidity, neonatal mortality and morbidity (only one study) **Other:** maternal birth experience, healthcare resource utilisation |
| Ralston 2014 LOW narrative | **Population**: inpatients with acute bronchiolitis (child aged 0–17 years) **Intervention:** active quality improvement (QI) intervention (educational campaigns, creation or active dissemination of clinical pathways or guidelines, implementation of an evidence-based order set, and/or adoption of a respiratory score to clinically manage patient care) aimed at improving or standardizing care of inpatients with acute bronchiolitis **Comparison:** usual care **Study design:** cluster randomized trials, before-and-after study studies, cohorts, and QI reports | NR | inception - October 2013 | 1998 - 2014 | Overall: 14 Restrosp. Cohort: 1 ITS: 11 UBA: 1  case-control: 1 | RoB assessed, but overall assessment NR | **Utilization/ordering of LVC:** bronchodilator (Beta-Agonist) use, systemic corticosteroid use, chest radiography, antibiotic use **Safety Outcomes:** readmission rate, length of stay  **Costs:** costs |
| Rietbergen 2020 MODERATE meta-analysis for restraint use, narrative | **Population:** Nurses and/or nurse practitioners working in hospitals, nursing homes, long-term care facilities, and community settings **Interventions:** reducing low value care in nursing, may include: educational interventions, audit and feedback, local opinion leaders, patient-medicated interventions, public release of performance data, financial intervention **Comparison:** All control groups (e.g. usual care, other active intervention active interventions aiming at reducing the overuse of low value medical imaging, or pre-intervention comparisons) **Study design:** all studies that use a reference group | **Study design:** Case studies of individual patients, letters, and editorials | inception - January 2020 | 1997 - 2018 | Overall: 27 cRCT and RCT: 10 UBA: 12 CBA: 5 | **CBA, cRCTS and RCT:** RoB assessed, but overall assessment NR **UBA**: Poor: 12 | **Utilization/ordering of LVC:** percentage of restraint use, percentage of catheter utilization, rate of antibiotic prescribing, rate of liver function tests, rate of antipsychotic prescribing |
| Sypes 2020 LOW meta-analysis and narrative | **Population:** use of low-value care with or without intervention **Intervention:** engaged patients in their aim to reduce the use of low-value care (clinical intervention that lacks efficacy, has risks that outweigh benefits, or is not cost-effective) **Study design:** experimental (e.g. RCT, NRCT) or quasi-experimental (e.g. controlled before-after) studies | **Intervention:** intervention to reduce low-value care that solely targeted clinicians, low-value practice of interest was not a medical test or treatment (e.g., bed rest, use of physical restraints) | inception - November 2019 | 2001 - 2018 | Overall: 22 RCT: 9 NRCT: 2 UBA: 4 CBA: 2 prospective observational: 2 quality improvement project: 3 | **RCT:** low: 5, unclear: 3, high: 1 **NRCT:** lower quality: 2  **UBA:** average quality: 1, lower quality: 2 **CBA:** higher quality: 2  **prospective observational:** higher quality: 2 **quality improvement project:** higher quality: 1, average quality: 1, lower quality: 1 | **Utilization/ordering of LVC:** use of low-value care **Cost:** costs of overuse of low-value practices |
| Xiong 2018 MODERATE narrative | **Population**: adult patients (age ≥ 18 years **Intervention**: interventions aimed at improving appropriateness, awareness of device presence, or prompt removal of central venous catheter (CVC) to prevent central-line–associated bloodstream infections (CLABSIs) **Study design:** experimental and observational studies with or without a control group | NR | inception - 2018 | 2010 - 2018 | Overall: 14 RCT: 1 QES: 6 (5 UBA) Cohort: 4  ITS: 3 | **RCT:** medium risk: 1 **QES** (UBA): B (medium quality): 5, C (low quality): 1  **Cohort:** low risk: 4 **ITS:** A (high quality): 3 | **Utilization/ordering of LVC:** central venous catheter use, peripherally inserted central venous catheters use, Total central venous catheter days **Safety Outcomes:** central-line–associated bloodstream infection (CLABSI) rate, peripherally inserted central venous catheters-related complications, hospital-related outcomes (LOS and mortality) **De-implementation Outcomes:** Inappropriate CVC reduction rate, compliance **Costs:** cost analysis |
| Xu 2021 LOW narrative | **Population:** patients in intensive care units who were critically ill or a short stay for observation **Intervention:** pharmacist-led intervention (shared care approach or primary decision makers) to impact on the use of stress ulcer prophylaxis in patients **Comparison/study design:** any study design with a comparator group of usual care or other healthcare's interventions | **Population:** no separate results for ICU reported | inception - March 12, 2020 | 2005-2019 | Overall: 8 (9 articles) Prosp. Cohort: 2 Restrosp. Cohort: 6 | Fair: 6 Poor: 2 | **Appropriateness of interventions:** inappropriate use of stress ulcer prophylaxis pharmacotherapy **Safety Outcomes:** incidence of Clostridioides difficile-associated disease, pneumonia or hospital-acquired pneumonia, gastrointestinal bleeding, and thrombocytopenia **Costs:** cost of medications |

Notes: ARI: acute respiratory infection; BMPs: basic metabolic panel; BZD: Benzodiazepines; CBA: controlled before-after study; CBCs: complete blood counts; CCT: controlled clinical trial; CDI: Clostridioides difficile infection; CD-ROMS: Compact Disc Read-Only Memory; CDSS: Clinical Decision Support Systems; CHWs: Community Health Workers; CLABSIs: central-line–associated bloodstream infections; CNS: central nervous system; COPD: chronic obstructive lung conditions; CPOE: computerized physician order entry; cRCT: cluster-randomized controlled trial; CT: computed tomography; CVC: central venous catheter; DDD: defined daily doses; DVDs: digital video disc; ED: emergency department ; e.g.: exempli gratia; EHR: electronic health record; EPOC: Effective Practice and Organization of Care; HCPs: health care professionals; HIV: human immunodeficiency viruses; HTTP: Hypertext Transfer Protocol; ICU: intensive care unit; i.e.: id est;  IT: information technology; ITS: interrupted time series; LMICs: lower-middle-income countries; LOS: length of stay; LRTI: lower respiratory tract infections; LTCF: long-term care facilities; LVC: low value care; MA: meta-analyses; MMAT: Mixed Methods Appraisal Tool; NR: not reported; NRCT: non-randomized controlled trial; NRSI: nonrandomized intervention studies; NRTs: non-randomized trials; OECD: Organisation for Economic Co-operation and Development; PICO: components (population, intervention, comparison, outcome) to define research questions; PSA: Prostate-Specific Antigen; QES: quasi-experimental study; QI: quality improvement; RCT: randomized controlled trial; RoB: risk of bias; RTIs: ; SR: systematic review; STAT: Short Turn Around Testing; TFT: thyroid function tests; UBA: uncontrolled before-after study; UTI: urinary tract infection; URTI: upper respiratory tract infection; Y: year

# eTable 9: Setting and healthcare practice targeted

| **Author Y,  Confidence, Method** | **Healthcare practice** | **Medical specialties** | **Setting** | **Rationale category** | **specify, guideline mentioned** | **4 R's  (remove, replace, reduce, restrict)** | **Location, included studies** |
| --- | --- | --- | --- | --- | --- | --- | --- |
| Drug treatments (antibiotics) | | | | | | | |
| Coxeter 2015 MODERATE meta-analysis | antibiotic prescribing for respirator infections | Family medicine | primary care  outpatient | 3 | NR | reduce | NoA: 2  Eur: 8 |
| Davey 2017 HIGH meta-analysis | antibiotic prescribing for hospital inpatients | Emergency medicine, various specialists in hospitals | tertiary care  inpatient | 3 | Antimicrobial Resistance Strategy (UK 2013 - 2018) | reduce | NoA: 96 SoA: 8 Eur: 87 As: 22 Au: 8 |
| deBont 2015 LOW narrative | antibiotic prescribing | Family medicine | primary care outpatient | 3 | none | reduce | Eur: 5 NR: 3 |
| Doan 2014 HIGH meta-analysis | rapid viral diagnostic imaging | Pediatrics | primary care  outpatient | 1 | NR | replace | NoA: 4 |
| Fleming 2013 LOW narrative | antibiotic prescribing in long-term-care facilities (LTCF) for UTI, RTI and skin diseases | Family medicine | long-term care  inpatient | 3 | NR | reduce | NoA: 3  Eur: 1 |
| Lane 2018 LOW narrative | antimicrobial prescribing in common (respiratory, gastrointestinal, urinary and skin) infections | Family medicine | primary care  outpatient | 3 | NR | reduce | NoA: 3 |
| Lim 2020 LOW narrative | antibiotic prescribing | Evaluate across multiple disciplines, country-level | primary and secondary/ tertiary care inpatient/ outpatient | 3 | WHO’s Global Action Plan on Antimicrobial Resistance (2015) | reduce | NoA: 4 SoA: 2 Eur: 16 As: 10 Au: 2 |
| Martinez-Gonzalez 2020 HIGH meta-analysis | antibiotic prescribing in upper respiratory tract infections | Family medicine | primary care  inpatient and outpatient | 3 | international clinical practice guidelines (NICE guidance) | reduce | NoA: 1  Eur: 9 As: 3 |
| Mortazhejri 2020 HIGH meta-analysis and narrative | antibiotic prescribing in upper respiratory tract infections | Family medicine | primary care  outpatient | 3 | NR | reduce | NoA: 2  Eur: 8 As: 2 Au: 1 |
| Nabovati 2021 LOW narrative | antibiotic prescribing in primary care | Family medicine | primary care and secondary care outpatient | 3 | none | reduce | NoA: 11  Eur: 5 As: 2 |
| Nair 2021 MODERATE narrative | antibiotic prescribing in outpatient or ambulatory care in LMICs | Family medicine, pharmacies | primary care  outpatient | 3 | NR | reduce | As: 7 Af: 6 |
| Nguyen 2019 MODERATE narrative | antibiotic prescribing in care homes | Gerontology | nursing homes inpatients | 3 | NR | reduce | NoA: 3 Eur: 2 |
| O'Sullivan 2016 HIGH narrative | antibiotic prescribing in upper respiratory tract infections | Family medicine | primary care  outpatient | 3 | recommendations by Centers for Disease Control and Prevention (CDC) and the American Society for Microbiology | reduce | NoA: 1 Eur: 1 |
| Raban 2023 LOW narrative | antibiotic prescribing | Family and internal medicine | primary care outpatient | 3 | NR | reduce | NoA: 6 Eur: 8 As: 3 Au: 2 |
| Rajar 2020 MODERATE narrative | antibiotic prescribing in neonates | Neonatology | tertiary care inpatient | 3 | NR | reduce | NoA: 6 Eur: 2 As: 4 |
| Siachalinga 2022 LOW narrative | antibiotic prescribing in hospital inpatients | not specified | primary care and secondary/ tertiary care inpatient | 3 | AMR-NAP | reduce | Af: 28 |
| Smedemark 2022 MODERATE meta-analysis | Point-of-Care tests (C-Reactive Protein, procalcitonin, white blood cell count) for infection biomarkers to guide antibiotic treatment | Family medicine | primary care outpatient | 3 | different national guidlines for c-reactive-protein measurement, NICE clinical guidelines | reduce | Eur: 10  As: 3 (1 in Russia) |
| Spurling 2017 HIGH meta-analysis | delayed antibiotics use, immediate antibiotic use and no antibiotics for RTI | Family medicine, emergency medicine, paediatrics | primary and secondary care inpatient/ outpatient: NR | 2 | NR | reduce | NoA: 4 Eur: 5 As: 1 Au: 1 |
| VanDijck 2018 MODERATE narrative | antibiotic prescribing in low- and middle-income countries | Emergency medicine, Anasthesia, Internal medicine, Pediatrics, Surgery | secondary/ tertiary care  inpatient | 3 | NR | reduce | SoA: 7  Eur: 3 As: 15 Af: 3 (one study both SoA and As) |
| Vodicka 2013 LOW narrative | antibiotic prescribing for acute RTIs in children | Family medicine, Emergency, paediatric primary care | primary care outpatient | 3 | NR | Reduce | NoA: 10  Eur: 3 As: 3 Au: 1 |
| Drug treatments (opiods) | | | | | | | |
| Badreldin 2023 LOW narrative | opioid prescribing for postpartum patients | Obstetrics and gynecology | secondary care  inpatient/ outpatient | 3 | CDC Guideline for prescribing opioids for chronic pain; American College of Obstretricians and Gynecologists' Committee on Clinicla Consensus-Obstetricts | reduce | NoA: 24 |
| Daoust 2022 MODERATE meta-analysis and narrative | opioid prescribing at point of discharge from the ED | Emergency medicine | primary or secondary/ tertiary care inpatient/ outpatient: NR | 3 | multiple hospital or nation-wide guidelines mentioned, including CDC | reduce | NoA: 58 Au: 5 |
| Phinn 2023 MODERATE narrative | opioid prescribing for noncancer pain upon hospital discharge | Emergency department, surgery, gynaecology and other | secondary/ tertiary care  inpatient and outpatient | 3 | NR | reduce | NoA: 34 Eur: 1 Au: 8 |
| Zhang 2020 MODERATE narrative | opioid prescribing | Surgery | secondary/ tertiary care  inpatient | 1 | Canadian Guideline for Opioids for Chronic Non-Cancer Pain, CDC Guidelines on Opioid Prescribing | reduce | NoA: 23 Au: 1 |
| Drug treatments (antipsychotics, benzodiazepines) | | | | | | | |
| Birkenhäger-Gillesse 2018 LOW meta-analysis | antipsychotics, antidepressants, anxiolytics, and/or hypnotics including benzodiazepines | Psychiatry | secondary care/ long-term care  inpatient | 2 | practice guidelines for NPS | replace | NoA: 2 Eur: 8 Au: 1 |
| Mokhar 2018 LOW narrative | BZD and z-drug | geriatrics, family medicine | primary care/ tertiary care (long term care centers) inpatient/ outpatient | 3 | Beers Criteria Update Expert Panel | reduce | NoA: 4 Eur: 12 Au: 4 |
| ThompsonCoon 2014 MODERATE narrative | antipsychotic prescribing in patients with dementia | Psychiatry | tertiary care inpatient | 2 | NR | reduce | NoA: 13  Eur: 7 Au: 2 |
| Laboratory tests | | | | | | | |
| Dunn 2021 LOW narrative | tests for CDI diagnosis (nucleic acid amplification tests (NAATs) for toxin B genes) | NR | secondary/tertiary care inpatient | 3 | Infectious Diseases Society of America and Society for Healthcare Epidemiology of America guidelines (e.g., Clinical practice guidelines for Clostridium difficile infection  in adults and children: 2017 update) | Reduce | NoA: 11 |
| Kobewka 2015 MODERATE narrative | various tests | Laboratory diagnostics, various medical specialities | primary and secondary/ tertiary care inpatient and outpatient | 1 | NR | reduce | NoA: 61 Eur: 27 As: 1 Au: 10 Other: 10 |
| Yeshoua 2023 LOW narrative | complete blood counts, comprehensive metabolic panel and BMPs (basic metabolic panel) | Internal medicine | secondary care inpatient | 3 | choosing wisely | reduce | NR |
| Zare 2021 LOW narrative | diverse (HB antigen tests, anti-PF4/ heparin antibody  enzyme-linked  immunosorbent  assay testing, freeT3/T4, anti-PF4/ Erythrocyte Sedimentation Rate, clostridium difficile testing) | NR | secondary/ tertiary care inpatient | 3 | NR | reduce | NoA: 13 Eur: 3 |
| Zhelev 2016 MODERATE narrative | thyroid function tests (TSH, FT4, FT3, TPOAb, TRAb) and other tests (diverse, e.g. complete blood count) | Family medicine, Internal Medicine | primary and secondary/ tertiary care inpatient and outpatient | 1 | national guidelines for the use of TFTs in the UK | reduce | NoA: 12  Eur: 11 Au: 4 |
| Diagnostic imaging | | | | | | | |
| Belavy 2022 MODERATE meta-analysis | X-Ray, CT, MRI | NR | primary and secondary/tertiary care inpatient and outpatient | 3 | Primary care guidelines from Canada and Finland | reduce | NR |
| Dunne 2022 MODERATE narrative | computed tomography usage in the ED | Emergency medicine | secondary/ tertiary care inpatient/ outpatient: NR | 3 | Choosing Wisely, Academic Emergency Medicine consensus conference | reduce | NoA: 126  Eur: 8 As: 8 Au: 5 Af: 2 |
| Kjelle 2021 LOW narrative | musculoskeletal, neurological and vascular imaging | Diagnostic radiology | primary and secondary/ tertiary care inpatient and outpatient | 4 | iRefer, iGuide, NICE "Do-not-do list", Choosing Wisely | Reduce | NoA: 85  Eur: 5 As: 1 Au: 4 |
| Zare 2022 LOW meta-analysis and narrative | imaging for CNS (central nervous system) injuries | including primary, emergency, and specialist care | secondary/ tertiary care inpatient and outpatient | 3 | CCSR, NEXUS, New Orleans Criteria, Pediatric Emergency Care Applied Research Network (PECARN), Canadian CT Head Rule (CCTHR), and CT in Head Injury Patients Prediction Rule | Reduce | NoA: 9  Eur: 1 Au: 1 |
| Other (imaging, laboratory tests, physiological tests) | | | | | | | |
| Foster 2020 LOW narrative | laboratory tests, red blood cell transfusions | Anesthesiology | secondary/ tertiary care  inpatient/ outpatient: NR | 1 | Choosing Wisely; Critical Care Societies Collaborative within their “Five Things Physicians and Patients Should Question” list | reduce | NoA: 11 SoA:  Eur: 4 As: 1 |
| Takada 2020 LOW narrative | laboratory tests, imaging and physiological tests | Family medicine | primary care outpatient | 3 | USPSTF Sceening for breast cancer; Choosing Wisely - promoting conversations between patients and clinicians | reduce | NoA: 3 Eur: 11 Au: 2 |
| Xie 2022 MODERATE narrative | diagnostic imaging and laboratory tests | not specified | primary and secondary/tertiary care inpatient/ outpatient: NR | 6 | NR | reduce | NoA: 4  Eur: 3 As: 2 Au: 2 |
| Other interventions | | | | | | | |
| Baptista 2018 LOW meta-analysis | prostate cancer screening | Urology | primary care and secondary/ tertiary care inpatient/ outpatient: NR | 3 | Guidelines issued by the European Association of Urology, American Cancer Society, American College of Physicians | restrict | NoA: 5  Eur: 1 Au: 1 |
| Chen 2018 HIGH narrative | unnecessary caesarean section | Obstetrics and gynecology | primary and secondary/ tertiary care inpatient and outpatient | 3 | different national clinical societies | reduce | NoA: 9 SoA: 2 Eur: 6 As: 10 Au: 2 |
| Ralston 2014 LOW narrative | bronchodilator, corticosteroid or antibiotic prescribing for bronchiolitis in children, chest ray | Pediatrics | secondary/ tertiary care inpatient | 3 | American Academy of Pediatrics (AAP) bronchiolitis guideline | reduce | NoA: 11 NR: 3 |
| Rietbergen 2020 MODERATE meta-analysis for restraint use, narrative | low value nursing procedures such as physical restraints, antibiotic/antipsychotic prescribing, urinary catheters, liver function tests | gerontology, intensive care, emergency, pediatrics, neurology, orthopedics, psychiatry, NR (5) | primary and secondary/ tertiary care inpatient and outpatient | 3 | Choosing Wisely | reduce | NoA: 11 Eur: 11 As: 3 NR: 2 |
| Sypes 2020 LOW meta-analysis and narrative | drug treatment (antibiotics, other), screening, operative treatments, laboratory tests, diagnostic imaging | Emergency medicine, pediatrics, obstetrics and gynecology, geriatrics, family medicine | primary and secondary/ tertiary care inpatient/ outpatient | 3 | NR | reduce | NoA: 15 Eur: 4 As: 2 Au: 1 |
| Xiong 2018 MODERATE narrative | central venous catheter (CVC) | emergency medicine, internal medicine, intensive care, general medicine | secondary/ tertiary care inpatient | 3 | Michigan Appropriateness Guide for Intravenous Catheters (MAGIC) | reduce | NoA: 12  Eur: 1 As: 1 |
| Xu 2021 LOW narrative | stress ulcer prophylaxis pharmacotherapy | Internal medicine | secondary/ tertiary care inpatient | 3 | NR | reduce | NoA: 6 As: 1 Au: 1 |

Notes: AAP: American Academy of Pediatrics; Af: Africa; AMR-NAP: Antimicrobial Resistance National Plan; Anti-PF4: anti-platelet factor 4; As: Asia; Au: Australia; BMPs: basic metabolic panel; BZD: Benzodiazepines; CCSR: Canadian C-Spine Rule; CCTHR: Canadian CT Head Rule; CDC: Centers for Disease Control and Prevention; CDI: Clostridioides difficile infection; CNS: central nervous system; CT: computed tomography; CVC: central venous catheter; ED: emergency department; e.g.: exempli gratia; Eur: Europe; FT3: free tri-iodothyronine; FT4: free thyroxine; HB: hepatitis B; LMICs: lower-middle-income countries; LTCF: long-term-care facilities; MAGIC: Michigan Appropriateness Guide for Intravenous Catheters; MRI: Magnetic resonance imaging; NAATs: nucleic acid amplification tests; NEXUS: National Emergency X-Radiography Utilization Study; NICE: National Institute for Health and Care Excellence; NoA: North America; NPS: neuropsychiatric symptoms; NR: not reported; PECARN: Pediatric Emergency Care Applied Research Network; RTI: respiratory tract infection; SoA: South America; T3: triiodothyronine; T4: thyroxine; TFTs: thyroid function tests; TPOAb: thyroid peroxidase antibody; TRAb: thyrotropin receptor antibody, TSH: thyroid stimulating hormone; UK: United Kingdom; USPSTF: United States Preventive Services Taskforce; UTI: urinary tract infection; WHO: World Health Organization; Y: year; z-drug: Zopiclone, eszopiclone, zaleplon and zolpidem

# eTable 10: De-implementation strategies reported

| **Author Y, Confidence, Method** | **Higher level strategies** | **de-implementation strategies short (mentioned at least once)** | **taxonomy** | **single/multiple strategies** | **actor of de-implementation** |
| --- | --- | --- | --- | --- | --- |
| Drug treatments (antibiotics) | | | | | |
| Coxeter 2015 MODERATE meta-analysis | train and educate stakeholders, support clinicians, use evaluative and iterative strategies, engage consumers | interventions to implement or foster shared-decision making including:  conduct educational outreach visits, distribute educational material for patients, communication tool (educational material for patient), conduct ongoing training, make training dynamic, clinical practice guidelines, educational material for clinicians, remind clinicians, audit and provide feedback, prepare patients/consumers to be active participants | no taxonomy, definitions via inclusion criteria defined | single: 2 multiple: 8 | general practitioners, clinicians, parents |
| Davey 2017 HIGH meta-analysis | train and educate stakeholders, develop stakeholder relationships, utilize financial strategies, use evaluative and iterative strategies, support clinicians | intervention:  distribute educational materials, conduct educational meetings,  audit and provide feedback, educational outreach visits , remind clinicians , **change physical structure and equipment (restriction by removal),** accountability tool (review and make change, restriction by expert approval, restriction by removal), dissemination of educational materials, policy and regulation, alter incentives/ allowance structures (financial incentives), conduct local consensus discussions,  Intervention functions:  education,  enablement,  environmental restructuring, persuasion,  restriction | intervention (EPOC) and intervention function (Michie 2011) | single: 77 multiple: 144 | multidisciplinary team, specialist physician (infectious diseases or microbiology), department physicians (emergency or critical care), pharmacists |
| deBont 2015 LOW narrative | train and educate stakeholders, utilize financial strategies | educational material for patients, assess and redesign workflow (delayed antibiotic prescribing),  change physical structure and equipment (eye swab), | no taxonomy, definitions via inclusion criteria defined | single: 5 multiple: 3 | GPs, nurses |
| Doan 2014 HIGH meta-analysis | change infrastructure and workflow | change physical structure and equipment | no taxonomy | single: 4 | physicians |
| Fleming 2013 LOW narrative | train and educate stakeholders, develop stakeholder relationships, support clinicians | policy and regulations,  audit and provide feedback,  conduct educational meetings,  distribute educational material,  make training dynamic,  conduct educational outreach visits,  remind clinicians | no taxonomy, definitions via inclusion criteria defined | single: 1 multiple: 3 | doctors, physicians, nurses, medical officers, pharmacist prescribers |
| Lane 2018 LOW narrative | train and educate stakeholders, support clinicians | facilitate relay of clinical data to providers,  conduct ongoing meetings,  distribute educational materials,  remind clinicians | no taxonomy, definitions via inclusion criteria defined | single: 3  (only reported for 3 studies) | clinicians, GPs |
| Lim 2020 LOW narrative | use evaluative and iterative strategies, train and educate stakeholders, utilize financial strategies, support clinicians | use advisory boards and workgroups, conduct ongoing training, develop and organize quality monitoring systems, distribute educational materials policy and regulations, use other payment schemes,  policy and regulation conduct educational meetings, use other payment schemes, distribute educational material, conduct educational meetings, audit and provide feedback | self-developed, definitions provided | single: 25 multiple: 9 | countries (health professionals, institutions, campagners) |
| Martinez-Gonzalez 2020 HIGH meta-analysis | train and educate stakeholders, support clinicians, change infrastructure and workflow | change physical structure and equipment,  conduct ongoing training, distribute educational materials, remind clinicians, make training dynamic, conduct educational meetings | no taxonomy, definitions via inclusion criteria defined | single: 2 multiple: 11 | physicians, nurses |
| Mortazhejri 2020 HIGH meta-analysis and narrative | train and educate stakeholders, engage consumers, change infrastructure and workflow | assess and redesign workflow,  use mass media, distribute educational materials for patients, educational meetings, communication tool | self-developed, definitions not provided | single: 12 multiple: 1 | physicians |
| Nabovati 2021 LOW narrative | train and educate stakeholders, support clinicians, use evaluative and iterative strategies | remind clinicians (CDSS), conduct ongoing training, audit and provide feedback, conduct cyclical small tests of change | no taxonomy, definitions via inclusion criteria defined | single: 11 multiple: 7 | physicians |
| Nair 2021 MODERATE narrative | train and educate stakeholders, support clinicians, change infrastructure and workflow, use evaluative and iterative strategies, develop stakeholder interrelationships | remind clinicians, conduct ongoing training, change physical structure and equipment, conduct educational meetings, develop and organize quality monitoring systems, assess and redesign workflow, audit and provide feedback, conduct local consensus discussions, distribute educational materials, use advisory boards and workgroups | intervention (EPOC) and intervention function (Michie 2011) | single: 4 multiple: 9 | prescribers (doctors, informal doctors, nurses, pharmacists, CHWs) |
| Nguyen 2019 MODERATE narrative | train and educate stakeholders, support clinicians, use evaluative and iterative strategies | conduct educational meetings, remind clinicians, conduct ongoing training, distribute educational materials, conduct educational outreach visits, audit and provide feedback, provide ongoing consultation | no taxonomy | multiple: 5 | staff, physician, nurses, home managers |
| O'Sullivan 2016 HIGH narrative | train and educate stakeholders, engage consumers, use evaluative and iterative strategies | distribute educational materials for patients, prepare patients/consumers to be active participants, communication tool, audit and provide feedback | self-developed, definitions provided | single: 1  multiple: 1 | physicians |
| Raban 2023 LOW narrative | use evaluative and iterative strategies, train and educate stakeholders, change infrastructure and workflow | audit and provide feedback, distribute educational materials, distribute educational materials for patients, assess and redesign workflow, accountability tools, public reporting | taxonomy of choice architecture techniques | single: 17 multiple: 2 | physicians |
| Rajar 2020 MODERATE narrative | use evaluative and iterative strategies, train and educate stakeholders, change infrastructure and workflow | distribute educational material, change physical structure and equipment,  conduct ongoing training, conduct educational meetings, audit and provide feedback, develop and organize quality monitoring systems | no taxonomy, definitions via inclusion criteria defined | single: 5 multiple: 7 | staff |
| Siachalinga 2022 LOW narrative | use evaluative and iterative strategies, train and educate stakeholders, support clinicians, develop stakeholder interrelationships, | policy and regulations,  audit and provide feedback, remind clinicians, conduct ongoing training, use advisory boards and workgroups, develop and organize quality monitoring systems, develop and implement tools for quality monitoring, | intervention (EPOC) and intervention function (Michie 2011) | single: 23 multiple: 5 | physicians, nurses, clinical pharmacists, clinical microbiologists, multi-disciplinary team |
| Smedemark 2022 MODERATE meta-analysis | support clinicians, change infrastructure and workflow, train and educate stakeholders | remind clinicians, change physicial structure and equipment, conduct ongoing trainings, provide educational materials for patients, prepare patients/consumers to be active participants, communication tool | no taxonomy, definitions via inclusion criteria defined | single: 8 multiple: 5 | clinicians |
| Spurling 2017 HIGH meta-analysis | change infrastructure and workflow | assess and redesign workflow | no taxonomy, definitions via inclusion criteria defined | single: 11 | physicians, patients |
| VanDijck 2018 MODERATE narrative | support clinicians, change infrastructure and workflow, train and educate stakeholders, use evaluative and iterative strategies, engage consumers | policy and regulations, change physical structure and equipment, audit and provide feedback, develop and implement tools for quality monitoring, use mass media, remind clinicians, distribute educational materials, conduct ongoing treatments, develop and organize quality monitoring systems, distribute educational materials for patients | intervention function (Michie 2011) | single: 19 multiple: 8 | physicians, nurses, pharmacists, prescribers, theater operating staff, intensive care unit staff, paediatricians, surgeons, clinical and medical operators, interns, students, midwives, all prescribers |
| Vodicka 2013 LOW narrative | train and educate stakeholders, support clinicians, develop stakeholder interrelationships, use evaluative and iterative strategies, engage consumers | distribute educational material, distribute educational material for patients, conduct educational outreach visits,  assess for readiness and identify barriers and facilitators, policy and regulation, conduct educational meetings, audit and provide feedback, prepare patients/consumers to be active participants, communication tool, conduct local consensus discussions, remind clinicians | no taxonomy | single: 4 multiple: 13 | clinicians |
| Drug treatments (opioids) | | | | | |
| Badreldin 2023 LOW narrative | train and educate stakeholders, support clinicians, change infrastructure and workflow, change in scope and nature of benefits and services | prepare patients/consumers to be active participants, distribute educational materials, communication tools, policy and regulations, conduct educational meetings, conduct ongoing training, assess and redesign workflow, remind clinicians, change physical structure and equipment, change in scope and nature of benefits and services | no taxonomy | multiple: 24 | physicians |
| Daoust 2022 MODERATE meta-analysis and narrative | support clinicians,train and educate stakeholders, change infrastructure and workflow, use evaluative and iterative strategies, develop stakeholder interrelationships, change in scope and nature of services | policy and regulations, use advisory boards and workgroups, conduct ongoing training, assess and redesign workflow, remind clinicians, develop and implement tools for quality monitoring, develop and organize quality monitoring systems, audit and provide feedback, change record systems, change in scope and nature of services | EPOC | single: 58 multiple: 5 | NR |
| Phinn 2023 MODERATE narrative | change infrastructure and workflow, train and educate stakeholders, use evaluative and iterative strategies, support clinicians, engage paients, develop stakeholder interrelationships | changes in electronic health record, distribute educational materials, distribute educational material to patients, assess and redesign workflow, develop and organize quality monitoring systems, develop and implement tools for quality monitoring, conduct educational outreach visits, communication tools, conduct ongoing training, policy and regulations, audit and provide feedback, accountability tool, use advisory boards and workgroups, remind clinicians, work with educational institutions, conduct local consensus discussion | self-developed, definitions provided | single: 27 multiple: 16 | physician, pharmacist, nurse, |
| Zhang 2020 MODERATE narrative | develp stakeholder interrelationships, support clinicians, train and educate stakeholders, change infrastructure and workflow, support clinicians, use evaluative and iterative strategies | conduct local consensus discussions, policy and regulations, conduct educational meetings, prepare patients/consumers to be active participants, develop and implement tools for quality monitoring, develop and organize quality monitoring systems, assess and redesign workflows, conduct ongoing training, communication tools, remind clinicians, change record systems, work with educational institutions, accountability tools | EPOC | single: 11  multiple: 13 | physicians, pharmacists |
| Drug treatments (antipsychotics, benzodiazepines) | | | | | |
| Birkenhäger-Gillesse 2018 LOW meta-analysis | educate and train stakeholders, change infrastructure and workflow, use evaluative and iterative strategies, support clinicians, change in scope and nature of benefits and services | conduct ongoing training, conduct educational outreach visitis, distribute educational materials, conduct educational meetings,  assess and redesign workflows, change in scope and nature of benefits and services, develop and organize quality monitoring systems, provide ongoing consultation, policy and regulations | self-developed, definitions provided | single: 3 multiple: 7 NR: 1 | Physicians, Nurses, Nursing assistants, All caregivers, All care staff, Non-care staff, psychiatric specialists, clinical pharamcists, occupational therapists, educator, project coordinator |
| Mokhar 2018 LOW narrative | train and educate stakeholders, support clinicians, provide interactive assistance | provide educational materials, develop and organize quality monitoring systems, conduct educational outreach visitis, provide clinical supervision | no taxonomy | single: 3 multiple: 2 | physicians, nurses |
| ThompsonCoon 2014 MODERATE narrative | train and educate stakeholders, use iterative and evaluative strategies, change infrastructure and workflow, support clinicians, develop stakeholder interrelationships, change in scope and nature of benefits and services | distribute educational material, conduct educational meetings, conduct educational outreach visits, inform local opinion leaders, audit and provide feedback, remind clinicians, create new clinical teams (skill mix changes), assess and redesign workflows (continuity of care), change service sites, change physical structure and equipment, change in scope and nature of benefits and services, develop and organize quality monitoring systems, change record systems, policy and regulations | EPOC and self-developed | single: 17 multiple: 5 | Educators, psychologist, occupational therapist or nurse supported by research team, old-age psychiatrist, trained nurse educator, Home management, specialist, clinical pharmacist, trained study pharmacist, trained registered nurses, nursing home management specialist, trained physician counselor, trained in-house nurse, researchers, community pharmacists, full-time psychiatric nurse supervised by a consultant old-age psychiatrist, clinical psychologist, psychiatrists and in-reach mental health nurses, doctors, community psychiatric nurses  NR: 1 |
| Laboratory tests | | | | | |
| Dunn 2021 LOW narrative | support clinicians, use evaluative and iterative strategies | remind clinicians, develop and organize quality monitoring systems | no taxonomy, definitions via inclusion criteria defined | single: 3 multiple: 8 | physicians |
| Kobewka 2015 MODERATE narrative | use evaluative and iterative strategies, train and educate stakeholders, change infrastructure and workflow, develop stakeholder interrelationships, support clinicians, utilize financial strategies, provide interactive assistance | develop and implement tools for quality monitoring, audit and provide feedback, conduct educational meetings, conduct ongoing trainings, changes in physical structure and equipment, provide clinical supervision, conduct local consensus discussions, policy and regulations, provide educational materials, change record systems, distribute educational materials, asess and redesign workflows, accountability tool, work with educational institutions, alter incentive/allowance structures, use advisory boards and workgroups | self-developed, definitions provided | single: 77 multiple: 32 | physicians |
| Yeshoua 2023 LOW narrative | support clinicians, train and educate stakeholders, use evaluative and iterative strategies, provide interactive assistance, change infrastructure and workflow, develop stakeholder interrelationships | conduct educational meetings, remind clinicians, conduct cyclical small tests of change, work with educational institutions, develop and organize quality monitoring systems, change record systems, audit and provide feedback, conduct ongoing training, change physical structure and equipment, accountability tool, assess and redesign workflows, use advisory boards and workgroups, policy and regulationx, conduct educational outreach visits, provide clinical supervision | self-developed, definitions provided | single: 14 multiple: 27 | physicians, medical staff |
| Zare 2021 LOW narrative | support clinicians, use evaluative and iterative strategies, develop stakeholder interrelationship, train and educate stakeholders | remind physicians, conduct local consensus discussions, accountability tool, change record systems, develop and organize quality monitoring systems, distribute educational materials, conduct educational meetings | no taxonomy, definitions via inclusion criteria defined | single: 16 | physicians |
| Zhelev 2016 MODERATE narrative | train and educate stakeholders, suppport clinicians, use evaluative and iterative strategies, utilize financial strategies | distribute educational materials, policy and guidelines, conduct educational meetings, conduct ongoing training, audit and provide feedback, develop and organize quality monitoring systems, remind clinicians, alter incentive/allowance structure | Oxman et al.'s taxonomy of interventions to improve professional practice | single: 14 multiple: 16  (information was extracted from textual description and does not correspond to number of included studies and table) | Physicians, GPs, nurse practicioners, medical staff, laboratory technicians, house officers, physician assistants, nurses |
| Diagnostic imaging | | | | | |
| Belavy 2022 MODERATE meta-analysis | change infrastructure and workflow, train and educate stakeholders, use evaluative and iterative strategies, develop stakeholder interrelationships, engage consumers, support clinicians, change in scope and nature of benefits and services | conduct educational meetings, distribute educational materials, assess and redesign workflows, change in scope and nature of benefits and services, audit and provide feedback, conduct educational outreach visits, remind clinicians, use advisory boards and workgroups, prepare patients/consumers to be active participants, obtain and use patients/consumers and family feedback, policy and regulations | EPOC | single: 2 multiple: 6 | Clinicians, Medical Residents |
| Dunne 2022 MODERATE narrative | change infrastructure and workflow, support clinicians, use evaluative and iterative strategies, adapt and tailor to context, train and educate stakeholders, engage consumers, change in scope and nature of benefits and services | change physical structure and equipment, accountability tool, assess and redesign workflows, policy and regulations, develop and organize quality monitoring systems, **staffing models and staff increase,** remind clinicians, use data warehouse techniques, audit and provide feedback, conduct educational meetings, conduct ongoing training, conduct local consensus discussions, distribute educational materials, communication tools, prepare patients/consumers to be active participants, create new clinical teams, change in scope and nature of benefits and services | self-developed, definitions not provided | single: 94 multiple: 55 | emergency medicine, multidisciplinary |
| Kjelle 2021 LOW narrative | train and educate stakeholders, suppport clinicians, use evaluative and iterative strategies, change infrastructure and workflow, utilize financial strategies | conduct ongoing training, conduct educational meetings, remind clinicians, audit and provide feedback, use data warehouse techniques, accountability tool, guideline and policy, develop and organize quality monitoring systems, alter incentive/allowance structure, assess and redesign workflows, prepare patients/consumers to be active participants, distribute educational materials, communication tool, change physical structure and equipment, create online learning communities, assess readiness and identify barriers and facilitators | self-developed, definitions not provided | single: 61 multiple: 34 | mostly referring physicians (n=90), imaging staff, patients and/or family members |
| Zare 2022 LOW meta-analysis and narrative | support clinicians,train and educate stakeholders, engage consumers, chagne infrastructure, | remind clinicians, policy and regulations, conduct educational meetings, inform local opinion leaders, communiation tool, prepare patients/consumers to be active participants, assess and redesign workflows, distribute educational materials, mandate change | no taxonomy, definitions via inclusion criteria defined | single: 4 multiple: 7 | physicians |
| Other (imaging, laboratory tests, physiological tests) | | | | | |
| Foster 2020 LOW narrative | use evaluative and iterative strategies, train and educate stakeholders, support clinicians, utilize financial strategies, change infrastructure and workflow, develop stakeholder interrelationships | audit and provide feedback, conduct ongoing training, policy and regulations, provide educational materials, remind clinicians, assess and redesign workflows, inform local opinion leader, mandate change, accuntability tool, provide clinical supervision, change physical structure and equipment, conduct local consensus discussion, create new clinical teams, alter incentive/allowance structures | EPOC | single: 0 multiple: 16 | leaders of the surgical and medical attending staff; all providers, physicians, physicians in-training, nurses, nurses in-training; nurses and unclear (medical staff, healthcare staff and members), unclear (heald of all the wards, senior medical staff), physician assistants, |
| Takada 2020 LOW narrative | train and educate stakeholders, support clinicians, use evaluative and iterative strategies, engage consumers, change infrastructure and workflow | distribute educational materials, conduct educational meetings, remind clinicians, audit and provide feedback, prepare patients/consumers to be active participants, change infrastrucutre | EPOC | single: 6 multiple: 10 | provider, organization, system |
| Xie 2022 MODERATE narrative | use evaluative and iterative strategies, adapt and tailor to context, support clinicians, change infrastructure and workflow | audit and provide feedback, remind clinicians, change record systems, use dataware house techniques | no taxonomy, definitions via inclusion criteria defined | single: 8 multiple: 3 | clinicians |
| Other interventions | | | | | |
| Baptista 2018 LOW meta-analysis | engage consumers | communication tools, prepare patients/consumers to be active participants | no taxonomy, definitions via inclusion criteria defined | single: 7 | physicians, patients |
| Chen 2018 HIGH narrative | train and educate stakeholders, engage consumers, provide interactive assistance, use evaluative and iterative strategies, utilize financial strategies, develop stakeholder interrelationships, support clinicians change infrastructure and workflow | conduct educational meetings for patients, prepare patients/consumers to be active participants, communication tools, distribute educational materials, make training dynamic, provide clinical supervision, audit and provide feedback, inform local opinion leaders, alter incentive/allowance structures, **staffing models and staff increase,** use mass media | self-developed, definitions provided | single: 20 multiple: 9 | physicians, nurses, midwifes, psychologists |
| Ralston 2014 LOW narrative | train and educate stakeholders, engage consumers, support clinicians, use evaluative and iterative strategies | distribute educational materials, assess and redesign workflows, prepare patients/consumers to be active participants, accountability tool, policy and regulations, remind clinicians, develop and organize quality monitoring systems | self-developed, definitions provided | single: 8 multiple: 6 | Physicians, nurses, respiratory therapists |
| Rietbergen 2020 MODERATE meta-analysis for restraint use, narrative | train and educate stakeholders, provide interactive assistance, support clinicians, change infrastructure and workflow, develop stakeholder interrelationships, change in scope and nature of benefits and services | conduct educational outreach visits, distribute educational materials, conduct educational meetings, provide clinical supervision, policy and regulations, conduct local consesus process, change physical structure and equipment, change in scope and nature of benefits and services, use advisory boards and workgroups | EchwPOC | single: 5 multiple: 20 single and multiple: 2 | clinical leader, local vendor, charge nurses, clinical nurse specialist, masters-prepared gerontologic nurse, nursing consultation, nurse specialist, registered nurse, ICU nurses, nurse practitioner, psychiatric nurses, pediatric intensive nurses, orthopaedic nurses, occupational therapist, registered health professionals, research team |
| Sypes 2020 LOW meta-analysis and narrative | train and educate stakeholders, support clinicians, engage consumers | distribute educational material for patients, prepare patients/consumers to be active participants, conduct edcuational meetings, communication tools, use mass media | no taxonomy | single: 19 multiple: 3 | patients, caregiver, physicians, pharmacists |
| Xiong 2018 MODERATE narrative | support clinicians, develop stakeholder interrelationships, use evaluative and iterative strategies, change infrastructure and workflow, train and educate stakeholders | remind clinicians, create new clinical teams, use advisory boards and workgroups, develop and organize quality monitoring systems, change physical structure and equipment, conduct educational meetings | no taxonomy | single: 8 multiple: 6 | nurses, physicians interdisciplinary team incl. management NR: 10 |
| Xu 2021 LOW narrative | use evaluative and iterative strategies, train and educate stakeholders, support clinicians, change infrastructure and workflow, develop stakeholder interrelationships | conduct local consensus discussions, policy and regulations, develop and organize quality monitoring systems, change record systems, distribute educational materials, conduct ongoing training | no taxonomy | single: 1 multiple: 6 NR: 1 | pharmacist, physician |

Notes: EPOC: Effective Practice and Organisation of Care; GPs: general practitioner; ICU: intensive care unit; n: number; NR: not reported; Y: year

# eTable 11: Details of de-implementation strategies reported

| **Details de-implementation strategies** | | | | | |
| --- | --- | --- | --- | --- | --- |
| **Author Y, Confidence, Method** | **Temporality reported (when is the target used), y/n** | **dosage reported (specify dosage of implementation strategy), y/n** | **theories, models, frameworks for de-implementation** | **barriers/facil. considered for selecting de-impl. strategies** | **targets patients, y/n** |
| Drug treatments (antibiotics) | | | | | |
| Coxeter 2015 MODERATE meta-analysis | n | n | n | n | y |
| Davey 2017 HIGH meta-analysis | n | n | n | n | n |
| deBont 2015 LOW narrative | y | n | n | n | y |
| Doan 2014 HIGH meta-analysis | n | n | n | n | n |
| Fleming 2013 LOW narrative | n | n | n | n | n |
| Lane 2018 LOW narrative | n | n | not reported in included studies | n | n |
| Lim 2020 LOW narrative | n | y | n | n | n |
| Martinez-Gonzalez 2020 HIGH meta-analysis | y | n | n | n | n |
| Mortazhejri 2020 HIGH meta-analysis and narrative | y | y | n | n | y |
| Nabovati 2021 LOW narrative | n | n | n | n | n |
| Nair 2021 MODERATE narrative | n | n | n | n | n |
| Nguyen 2019 MODERATE narrative | n | n | n | n | n |
| O'Sullivan 2016 HIGH narrative | y | y | n | n | y |
| Raban 2023 LOW narrative | n | n | n | n | n |
| Rajar 2020 MODERATE narrative | n | y | guidelines for design and implementation of a NICU-specific ASP developed by Patel and Saiman (1 study)  3 of the 12-steps program from Centers for Disease Control and Prevention, adjusted for the NICU population (1 study) | n | n |
| Siachalinga 2022 LOW narrative | n | n | n | n | n |
| Smedemark 2022 MODERATE meta-analysis | y | NR | n | n | n |
| Spurling 2017 HIGH meta-analysis | n | n | n | n | y |
| VanDijck 2018 MODERATE narrative | n | n | n | n | n |
| Vodicka 2013 LOW narrative | y | n | n | n | n (parents of children) |
| Drug treatments (opioids) | | | | | |
| Badreldin 2023 LOW narrative | n | n | n | n | n |
| Daoust 2022 MODERATE meta-analysis and narrative | n | n | n | n | n |
| Phinn 2023 MODERATE narrative | n | n | n | n | y |
| Zhang 2020 MODERATE narrative | n | n | n | n | n |
| Drug treatments (antipsychotics, benzodiazepines) | | | | | |
| Birkenhäger-Gillesse 2018 LOW meta-analysis | n | y | n | n | n |
| Mokhar 2018 LOW narrative | n | n | n | n | n |
| ThompsonCoon 2014 MODERATE narrative | n | y | n | n | n |
| Laboratory tests | | | | | |
| Dunn 2021 LOW narrative | y | n | n | n | n |
| Kobewka 2015 MODERATE narrative | n | n | n | n | n |
| Yeshoua 2023 LOW narrative | n | n | n | n | n |
| Zare 2021 LOW narrative | n | n | n | n | n |
| Zhelev 2016 MODERATE narrative | n | n | n | n | n |
| Diagnostic imaging | | | | | |
| Belavy 2022 MODERATE meta-analysis | n | n | n | n | y |
| Dunne 2022 MODERATE narrative | n | n | n | n | y (only one study) |
| Kjelle 2021 LOW narrative | n | n | n | n | n |
| Zare 2022 LOW meta-analysis and narrative | y | n | n | n | n |
| Other (imaging, laboratory tests, physiological tests) | | | | | |
| Foster 2020 LOW narrative | y | y | n | n | n |
| Takada 2020 LOW narrative | n | n | n | n | y |
| Xie 2022 MODERATE narrative | y | n | n | n | y |
| Other interventions | | | | | |
| Baptista 2018 LOW meta-analysis | n | n | n | n | y |
| Chen 2018 HIGH narrative | n | n | n | n | y |
| Ralston 2014 LOW narrative | n | n | n | n | n |
| Rietbergen 2020 MODERATE meta-analysis for restraint use, narrative | n | n | n | n | n |
| Sypes 2020 LOW meta-analysis and narrative | n | n | n | n | y |
| Xiong 2018 MODERATE narrative | n | n | n | n | n |
| Xu 2021 LOW narrative | n | n | n | n | n |

Notes: ASP: antibiotic stewardship program; n: no; NICU: neonatal intensive care unit; NR: not reported; Y: year; y: yes

# eTable 12: Study population in included SR

| **Author Y,  Confidence, Method** | **n overall  (intervention and control) HP, Center, Patient** | **single/multicentre** | **age Range** | **gender, female, range in %** | **observation period in months, Range** | **long-term outcomes (reported >1 year) (y/n; if yes: period in months)** |
| --- | --- | --- | --- | --- | --- | --- |
| Drug treatments (antibiotics) | | | | | | |
| Coxeter 2015 MODERATE meta-analysis | HP: 174 (NR: 7) Center: 433 (NR: 4) Patient: 491760 | single: 0 multi: 10 | HP: NR Patient: NR | HP: NR Patient: NR | 0.5 - 12 mo | y, > 12 months |
| Davey 2017 HIGH meta-analysis | HP: NR Center: NR Patient: NR | single: 178 multi: 43 | HP: NR Patient: NR | HP: NR Patient: NR | NR | n |
| deBont 2015 LOW narrative | HP: 197 (NR: 2) Center: 103 (NR: 2) Patient: 3522 (although 3407 stated in the text) | NR | HP: NR Patient: NR | HP: NR Patient: NR | 4 days - 1 mo | n |
| Doan 2014 HIGH meta-analysis | HP: NR Center: 4 Patient: 1595 | single: 4 | HP: NR Patient: 2mo - 21 years old | HP: NR Patient: NR | NR | n |
| Fleming 2013 LOW narrative | HP: 15 (only 1 study reported) Center: 88 Patient: 11271 (NR: 1) (data from the text) | multi: 4 | HP: NR Patient: 83.4 - 83.9 (NR: 2) | HP: NR Patient: NR | 3 - 17 mo | n |
| Lane 2018 LOW narrative | HP: 83 (NR: 1) Center: 53 (NR: 1)  Patient (#I): 28301 (NR: 2) | multi: 2  NR: 1 | HP: NR Patient: NR | HP: NR Patient: NR | 3 we - 36 mo (1 study NR) | n |
| Lim 2020 LOW narrative | NR | NR | HP: NR Patient: NR | HP: NR Patient: NR | 4 mo -17 y | y |
| Martinez-Gonzalez 2020 HIGH meta-analysis | HP: 173 (4 studies) Center: 226 (4 studies) Patient: 9844 | single: 2 multi: 11 | HP: NR Patients: 0 - 90 | HP: NR Patient: 56.1 % | NR | n |
| Mortazhejri 2020 HIGH meta-analysis and narrative | HP: NR Center: NR Patient: 7415 (NR: 1) | single: 1  multi: 6 NR: 6 | HP: NR Patient: 3 months - >18 years | HP: NR Patient: NR | NR | n |
| Nabovati 2021 LOW narrative | HP: NR Center: 539 Patient: NR | single: 2 multi: 16 | HP: NR Patient: NR | HP: NR Patient: NR | NR | n |
| Nair 2021 MODERATE narrative | HP: NR  Center: 303 (NR: 4) Patient: NR | multi: 12 NR: 1 | HP: NR Patient: NR | HP: NR Patient: NR | 1 - 48 mo | NR |
| Nguyen 2019 MODERATE narrative | HP: NR Nursing home: 132 Resident: 10550 (NR: 1) | multi: 5 | HP: NR Patient: ≥ 65 years, range NR | HP: NR Patient: NR | 15 - 20 mo | y, 15-20 months (study duration) |
| O'Sullivan 2016 HIGH narrative | HP: NR Center: NR Patient: 34350 | multi: 2 | HP: NR Patient: 6 mo < 18 years | HP: NR Patient: NR | NR | n |
| Raban 2023 LOW narrative | HP: 13140 (NR: 6) Center: 10484 (NR: 12) Patient: NR | NR | HP: NR Patient: NR | HP: NR Patient: NR | 3 - 24 mo (NR: 12) | n |
| Rajar 2020 MODERATE narrative | HP: NR Center: NR Patient: 29 997 | NR | HP: NR Patient: <34 weeks gestational age | HP: NR Patient: NR | NR | n |
| Siachalinga 2022 LOW narrative | HP: NR Center: 201 Patient: 203329 Patient (# I): 27849 | single: 19 multi: 9 | HP: NR Patient: NR | HP: NR Patient: NR | 3 mo - 5 y | n |
| Smedemark 2022 MODERATE meta-analysis | HP: NR (only 1 study reported) Center: 703 Patient: 10535 | multi: 13 | HP: NR Patient: 3mo - 65 years | HP: NR Patient: NR | 0.25 - 1 mo | n |
| Spurling 2017 HIGH meta-analysis | HP: 181 (NR: 5) Center: 141 Patient: 3497 | single: 5 multi: 6 | HP: NR Patient: <3 - 48 | HP: NR Patient: 43% - 65% | 3 - 60 mo | n |
| VanDijck 2018 MODERATE narrative | HP: NR Center: 188 Patient: 4844 (one study reported 1000 to 1022 patients per hospital for 22 hospitals included) | single: 18 multi: 9 | HP: NR Patient: NR | HP: NR Patient: NR | 9 - 283 we (5.4 years) | n |
| Vodicka 2013 LOW narrative | HP: 1975 (NR: 4) Center: 85 (NR: 14) Parent: 499 (NR: 16) Patient: 123064 (NR: 9) | multi: 3  NR: 14 | HP: NR  Patient: < 18 years old | HP: NR Patient: NR | 1 we - 29 mo | n |
| Drug treatments (opioids) | | | | | | |
| Badreldin 2023 LOW narrative | HP: NR Center: NR Patient: 60378 | NR | HP: NR Patient: NR | HP: NR Patient: 100% | NR | n |
| Daoust 2022 MODERATE meta-analysis and narrative | NR | NR | HP: NR Patient: NR | HP: NR Patient: NR | 6 - 12 mo | y; 12 months |
| Phinn 2023 MODERATE narrative | HP: 23 (only 1 study reported) Center: NR Patient: 739627 (NR: 4) | NR | HP: NR Patient: NR | HP: NR Patient: NR | 3 we - 7 y | n |
| Zhang 2020 MODERATE narrative | HP: NR Center: 24 Patient: 21204 | single: 22 multi: 2 | HP: NR Patient: NR | HP: NR Patient: NR | 4 - 47 mo (2 studies NR) | n |
| Drug treatments (antipsychotics, benzodiazepines) | | | | | | |
| Birkenhäger-Gillesse 2018 LOW meta-analysis | HP: NR Centre: 52 Patient: 716.642 (NR: 3) | single: 1 multi: 10 | HP: NR Patient: NR | HP: NR Patient: NR | 5 - 20 mo | n |
| Mokhar 2018 LOW narrative | HP: 631 Center: 68 Patient: 4079 | multi: 5 | HP: NR Patient: NR | HP: NR Patient: NR | 4 - 12 mo | n |
| ThompsonCoon 2014 MODERATE narrative | HP: 1470 (NR: 17) Center: 199 Patient: 19300 | single: 6 multi: 15 NR: 1 | HP: NR Patient: NR | HP: NR Patient: NR | 4 - 12 mo (2 studies NR) | n |
| Laboratory tests | | | | | | |
| Dunn 2021 LOW narrative | HP: NR Center: 18 (NR: 2) Patient (beds): 8190 (NR: 3) | single: 6 multi: 3 NR: 2 | HP: NR Patient: NR | HP: NR Patient: NR | 2 - 43 mo | n |
| Kobewka 2015 MODERATE narrative | HP: 376 (only 4 studies reported) Center: 153 (NR: 12) Patient: NR | single: 96 multi: 13 | HP: NR Patient: NR | HP: NR Patient: NR | NR | n |
| Yeshoua 2023 LOW narrative | NR | NR | HP: NR Patient: NR | HP: NR Patient: NR | 1 - 60 mo | y, 17/41 studies were conducted over more than 1 year, average length was 28.9 months |
| Zare 2021 LOW narrative | HP: NR Center: 20 Patient: 181678 (NR: 7) | single: 13 multi: 3 | HP: NR Patient: NR | HP: NR Patient: NR | 4 - 60 mo | n |
| Zhelev 2016 MODERATE narrative | HP: unclear reported Center: unclear reported Patient: unclear reported | single: 15 multi: 12 | HP: NR Patient: NR | HP: NR Patient: NR | 2 mo - 2 y | n |
| Diagnostic imaging | | | | | | |
| Belavy 2022 MODERATE meta-analysis | HP: NR Center: 382 (NR: 1) Patient: 168.460 (NR: 3) | multi: 8 | HP: NR Patient: NR | HP: NR Patient: NR | 5 - 12mo | n |
| Dunne 2022 MODERATE narrative | HP: NR Center: NR Patient: 2,529,855 | single: 111 multi: 38 | HP: NR Patient: NR | HP: NR Patient: NR | NR | n |
| Kjelle 2021 LOW narrative | HP: 5687 (NR: 85) Center: NR Patient: 2025308 (NR: 43) Patient (#I): 967874 (n=17) | single: 81 multi: 14 | HP: NR Patient: NR | HP: NR Patient: NR | NR | n |
| Zare 2022 LOW meta-analysis and narrative | HP: 8924 (NR: 6) Center: 52  Patient: 235494 (NR: 6) Patient (#I): 481148 (NR: 3) | single: 6 multi: 5 | HP: NR Patient: NR | HP: NR Patient: NR | 2 - 31 mo | n |
| Other (imaging, laboratory tests, physiological tests) | | | | | | |
| Foster 2020 LOW narrative | HP: NR Center: NR Patient: NR | single centre, single ICU: 9 single centre, multi-ICU: 4 multicentre: 2 single centre, # ICUs unclear: 1 | HP: NR Patient: NR | HP: NR Patient: NR | NR | unclear (differences between time 1 and time 3 reported, but time frame unclear) |
| Takada 2020 LOW narrative | HP: NR Center: NR Patient: NR | single: 2 multi: 14 | HP: NR Patient: NR | HP: NR Patient: NR | NR | y (but not NR, only sustainability of effect mentioned) |
| Xie 2022 MODERATE narrative | HP: 6393 (NR: 4) Center: 952 (NR: 2) Patient (and #I): 66953 (NR: 4) | single: 3 multi: 7 NR: 1 | HP: NR Patient: NR | HP: NR Patient: NR | 2 - 24 mo | n |
| Other interventions | | | | | | |
| Baptista 2018 LOW meta-analysis | HP: NR Center: NR Patient: 4714 | single: NR multi: NR | HP: NR Patient: 45-75 years | HP: NR Patient: 0% | NR | n |
| Chen 2018 HIGH narrative | HP: NR Center: NR Patient: NR | single: 3 multi: 6 NR: 20 | HP: NR Patient: NR | HP: NR Patient: NR | 4 - 84 mo, (3 studies NR) | n |
| Ralston 2014 LOW narrative | HP: NR Center: 23 Partient: 12519 | single: 12 multi: 2 | HP: NR Patient: NR | HP: NR Patient: NR | 3 - 21 mo | n |
| Rietbergen 2020 MODERATE meta-analysis for restraint use, narrative | NR | single: 12 multi: 15 | HP: NR Patient: NR | HP: NR Patient: NR | 1 - 14 mo | n |
| Sypes 2020 LOW meta-analysis and narrative | HP: NR Center: NR Patient: 102383 (one study estimated at ~ 20000; NR: 2) | NR | HP: NR Patient: NR | HP: NR Patient: NR | NR | n |
| Xiong 2018 MODERATE narrative | HP: NR Center: NR Patient: 415669 (NR: 5) | NR | HP: NR Patient: NR | HP: NR Patient: NR | 1 - 60 mo | n |
| Xu 2021 LOW narrative | HP: NR Centers: NR Patient: 3526 | single: 7 multi: 1 | HP: NR Patient: 18 - 90 | HP: NR Patient: 37% - 48% | 0.5 - 6 mo | n |

Notes: HP: health personnel; ICU: intensive care unit; mo: month(s); n: no; NR: not reported; we: week(s); Y: year; y: yes; # I: number of healthcare interventions

# eFigure 1: Frequency of higher-level ERIC de-implementation categories within the medical intervention categories


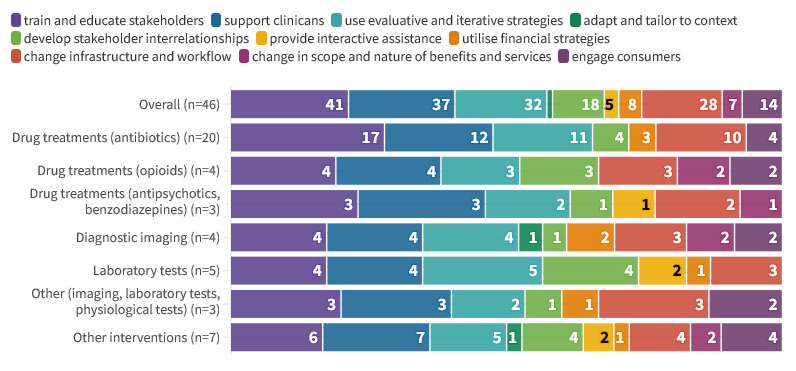

Supplement: Supplementary file 1 — Additional file 1. Including several tables and figures [file 13012_2024_1384_MOESM1_ESM.docx]
